# The catalytic subunit of DNA-PK regulates transcription and splicing of AR in advanced prostate cancer

**DOI:** 10.1172/JCI169200

**Published:** 2023-11-15

**Authors:** Beth Adamson, Nicholas Brittain, Laura Walker, Ruaridh Duncan, Sara Luzzi, Pasquale Rescigno, Graham Smith, Suzanne McGill, Richard J.S. Burchmore, Elaine Willmore, Ian Hickson, Craig N. Robson, Denisa Bogdan, Juan M. Jimenez-Vacas, Alec Paschalis, Jonathan Welti, Wei Yuan, Stuart R. McCracken, Rakesh Heer, Adam Sharp, Johann S. de Bono, Luke Gaughan

**Affiliations:** 1 Newcastle University Centre for Cancer, Paul O’Gorman Building, Newcastle Upon Tyne, United Kingdom.; 2 Newcastle University Biosciences Institute, International Centre for Life, Newcastle Upon Tyne, United Kingdom.; 3 Newcastle University Bioinformatics Support Unit, Medical School, Newcastle Upon Tyne, United Kingdom.; 4 Glasgow Polyomics, Wolfson Wohl Cancer Research Centre, College of Medical, Veterinary & Life Sciences, University of Glasgow, Glasgow, United Kingdom.; 5 The Institute for Cancer Research, London, United Kingdom.; 6 The Royal Marsden NHS Foundation Trust, London, United Kingdom.; 7 Division of Surgery, Imperial College London, London, United Kingdom.

**Keywords:** Endocrinology, Oncology, Prostate cancer

## Abstract

Aberrant androgen receptor (AR) signaling drives prostate cancer (PC), and it is a key therapeutic target. Although initially effective, the generation of alternatively spliced AR variants (AR-Vs) compromises efficacy of treatments. In contrast to full-length AR (AR-FL), AR-Vs constitutively activate androgenic signaling and are refractory to the current repertoire of AR-targeting therapies, which together drive disease progression. There is an unmet clinical need, therefore, to develop more durable PC therapies that can attenuate AR-V function. Exploiting the requirement of coregulatory proteins for AR-V function has the capacity to furnish tractable routes for attenuating persistent oncogenic AR signaling in advanced PC. DNA-PKcs regulates AR-FL transcriptional activity and is upregulated in both early and advanced PC. We hypothesized that DNA-PKcs is critical for AR-V function. Using a proximity biotinylation approach, we demonstrated that the DNA-PK holoenzyme is part of the AR-V7 interactome and is a key regulator of AR-V–mediated transcription and cell growth in models of advanced PC. Crucially, we provide evidence that DNA-PKcs controls global splicing and, via RBMX, regulates the maturation of AR-V and AR-FL transcripts. Ultimately, our data indicate that targeting DNA-PKcs attenuates AR-V signaling and provide evidence that DNA-PKcs blockade is an effective therapeutic option in advanced AR-V–positive patients with PC.

## Graphical abstract

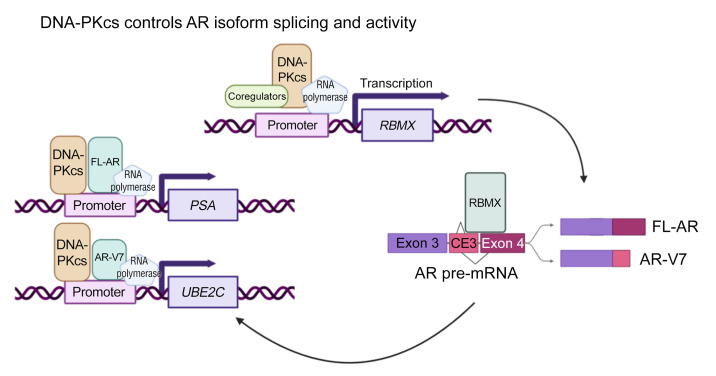

## Introduction

Prostate cancer (PC) is the third most common malignancy worldwide. The androgen receptor (AR) is a member of the nuclear hormone receptor family of transcription factors that regulates a canonical gene expression program involved in prostate homeostasis and, upon deregulation, cancer development (1, 2). The AR comprises an N-terminal transactivation domain (NTD), a DNA-binding domain (DBD), and a C-terminal ligand-binding domain (LBD), with a hinge region separating the DBD and LBD (3, 4). Binding of testosterone, or the more active derivative dihydrotestosterone (DHT), to the AR LBD activates nuclear translocation and subsequent transcription of proproliferation and -survival genes. Therefore, current treatments act to attenuate the AR signaling axis via the use of hormonal treatments such as androgen deprivation therapy and AR inhibitors (5, 6). Although the response to these treatments was initially successful, patients inevitably became resistant and progressed to the more advanced castration-resistant PC (CRPC), which, critically, in the majority of cases, remains dependent on the AR signaling axis for growth (3, 7).

Several resistance mechanisms contribute to the progression to CRPC, including mutations and amplification of the *AR* gene (8–11) and the generation of alternatively spliced forms of the full-length-AR (AR-FL), termed AR variants (AR-Vs) (12–15). In contrast to the AR-FL, AR-Vs lack the LBD but retain the transcriptionally potent NTD and the DBD (14). As such, they demonstrate constitutive transcriptional activity in castrate conditions and enhance expression of an androgenic signaling program, somewhat similar to the AR-FL (16–18). AR-V expression is more prevalent in advanced stages of the disease, and elevated levels of AR-Vs have been detected in upward of 80% of hormone therapy-treated patients, with AR-V7 and AR-V3 being the most commonly detected (19, 20). Critically, AR-Vs are refractory to the current repertoire of AR-targeting therapies and, hence, are able to support PC growth during hormone therapy (15, 21, 22). Although advances have been made toward the development of both NTD- and DBD-targeting agents, which have shown promise in preclinical models of PC (23–25), all clinically approved therapies targeting the AR have limited/no activity against AR-V function. Given this clinical unmet need, there is a major drive to develop treatments that can inhibit these aberrantly functioning receptors. Targeting AR-V coregulatory proteins that are required to facilitate AR-V function represents a tractable means for inactivating AR-Vs in advanced disease (26).

The DNA-dependent protein kinase (DNA-PK) is a serine/threonine protein kinase complex that consists of a Ku heterodimer (Ku70/Ku80) and a catalytic subunit (DNA-PKcs). It has a critical role in the DNA damage response (DDR) through facilitation of double-strand break repair via the nonhomologous end joining pathway (27–29). Outside of its direct role in the DDR, DNA-PKcs has pleiotropic cellular functions, including regulating cell cycle (30, 31), telomere maintenance (32, 33), metabolomics (34, 35), and transcription (36); the latter is evidenced by DNA-PKcs being required for SP1 transcriptional activity (36) and as a RNA polymerase II coregulator (37). In PC, DNA-PKcs has been shown to play a multifaceted role in driving disease progression: the enzyme (a) binds *cis*-regulatory elements of AR-target genes and enhances canonical AR-FL signaling (38); (b) supports metastatic spread in vitro and in vivo by upregulating a focal adhesion gene expression signature (38); and (c) engages with key glycolytic pathway enzymes, including pyruvate kinase M2 and phosphoglycerate kinase 1, to enable adaptation to the elevated energy demands of a hyperproliferative phenotype (34). Given that DNA-PKcs expression is also elevated in PC, the enzyme therefore represents a very promising therapeutic target in AR-FL–expressing PC.

In contrast to our understanding of the functional relationship between AR-FL and DNA-PKcs, interplay between AR-Vs and DNA-PKcs remains poorly characterized. Outside of the demonstration that the AR isoforms AR-V7 and ARv567es interacted with DNA-PKcs in CWR22Rv1 (38) and R1-D567 cells (39), respectively, major knowledge gaps remain in our understanding of whether the DNA-PK holoenzyme interacts with and controls AR-Vs, including the most clinically abundant AR-V7. We, therefore, reasoned that an unbiased interactome analysis of AR-V7 in CWR22Rv1-AR-EK cells, a CWR22Rv1 derivative expressing only AR-Vs (16), would validate AR-V7-DNA-PK interactions and support downstream analysis of DNA-PK–mediated regulation of AR-Vs in advanced PC. Moreover, in direct response to evidence that AR-Vs have been found to interact directly with DNA lesions (39), examining dynamic flux to the AR-V7 interactome in response to DNA damage may offer an improved insight into AR-V function during the DDR.

To that end, we developed and utilized an APEX2 peroxidase-mediated biotinylation pipeline (40), which has provided what we believe to be the first of its kind proximal protein interactome of AR-V7 in steady-state and in response to ionizing radiation (IR) in PC. We confirmed that AR-V7 interacts with DNA-PKcs, as well as Ku70 and Ku80, in the presence and absence of DNA damage, implicating a role of the DNA-PKcs holoenzyme in controlling AR-V7 function in steady-state conditions. Consistent with DNA-PKcs regulating AR-FL, we showed that DNA-PKcs was recruited to AR-V target genes and facilitated AR-V–mediated transcription in multiple PC cell lines. Furthermore, our data uncover an additional layer of complexity to DNA-PKcs cellular function by demonstrating it is a key node of splicing regulation, which, via RBMX, controls AR transcript maturation. Ultimately, our data provide a strong rationale for expediting DNA-PKcs blockade in advanced AR-V–positive patients with PC.

## Results

### Developing a proximity biotinylation assay to study the AR-V7 interactome.

**Figure 1:**
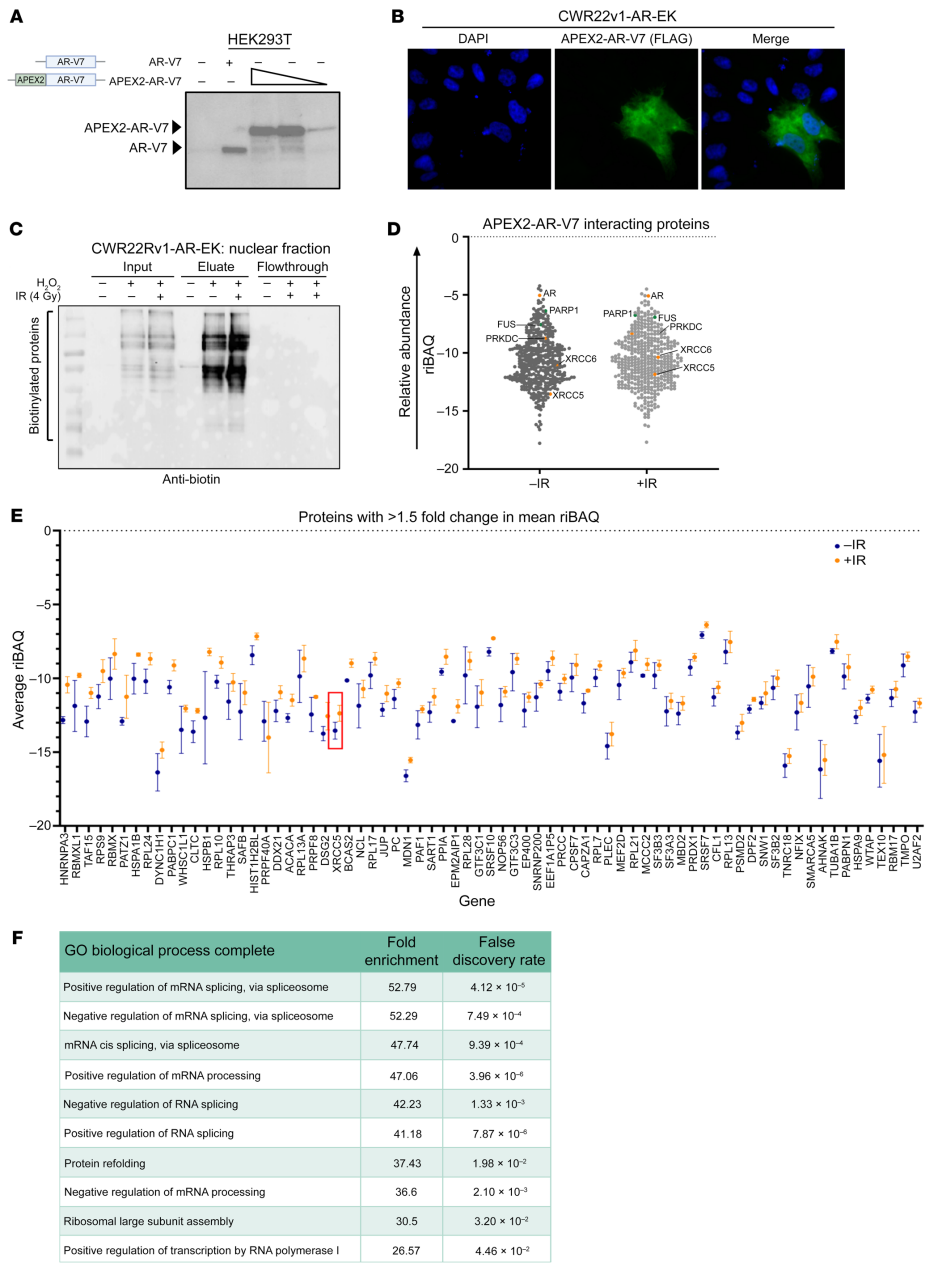
AR-V7 proximal biotinylation experiments identify known AR-V7 interactors and the DNA-PKcs holoenzyme. (**A**) Diagrammatic representation of APEX2-AR-V7 construct and anti-AR Western blot of HEK293T cells transiently transfected with either AR-V7 or increasing quantities of FLAG-APEX2-AR-V7 constructs. (**B**) 1 × 10^5^ CWR22Rv1-AR-EK cells were transfected with 2 μg of a FLAG-tagged APEX2-AR-V7 construct for 48 hours and again for an additional 24 hours prior to immunofluorescence using an anti-FLAG antibody. Magnification ×40. (**C**) 5 × 10^6^ CWR22Rv1-AR-EK cells were transfected with 10 μg pLV-FLAG-APEX2-AR-V7 and again 48 hours later prior to treatment with biotin-phenol and with or without IR (4 Gy) for 2 hours. In the –IR and +IR arms, H_2_O_2_ was added to cells to induce the labeling reaction. Cells were then quenched and harvested, and the cytoplasmic and nuclear fractions were isolated and quantified. 10 μg resultant nuclear lysate was analyzed by Western blotting using an HRP-linked anti-biotin antibody. Corresponding Ponceau Red stain is shown to indicate equal sample loading. (**D**) Plot of mean riBAQ scores of all APEX2-AR-V7–interacting proteins identified by mass spectrometry. AR and components of the DNA-PK holoenzyme are highlighted in orange, and two known AR interactors, PARP1 and FUS, are highlighted in green. (**E**) APEX2-AR-V7–interacting proteins that have a riBAQ score >1.5-fold in response to irradiation. Ku80 (XRCC5) is highlighted in a red box. Data points represent the mean of 2–3 replicates (depending on if the protein is identified in 2 or 3 replicates) ± SEM. (**F**) Top 10 biological processes that are enriched in the list of proteins that are more abundant AR-V7 interactors in response to irradiation.

Previous studies utilizing conventional immunoprecipitation and rapid immunoprecipitation mass spectrometry of endogenous proteins (RIME) have helped define the interactome of the AR variant ARv567es in R1-D567 PC cells in steady-state and in response to DNA damage (39, 41). To the best of our knowledge, however, there remain no interactome data for the most clinically abundant splice variant, AR-V7, in the presence and absence of IR. This is a key knowledge gap that could help identify tractable routes for AR-V blockade in advanced PC and define the function of AR-Vs during the DDR. We therefore developed a pipeline to identify AR-V7 interactors in PC cells using APEX2 peroxidase-mediated proximity biotinylation, an approach that is highlighted as offering improved sensitivity and specificity over RIME by enabling detection of transient interacting partners without the need for cross-linking, which can increase detection of nonspecific proteins (42). First, we generated a FLAG-tagged APEX2-AR-V7 fusion whose ectopic expression was nuclear (Figure 1, A and B) and enriched at *cis-*regulatory elements of canonical AR-target genes in CWR22Rv1-AR-EK and CWR22Rv1 cells to levels equivalent to untagged endogenous AR-V7 (Supplemental Figure 1, A and B; supplemental material available online with this article; https://doi.org/10.1172/JCI169200DS1) (16). To test the APEX2 peroxidase-mediated protein biotinylation capacity of the fusion, we subjected APEX2-AR-V7–expressing HEK293T and CWR22Rv1-AR-EK cells to no IR (–IR) or 4 Gy IR (+IR) and either 1- or 2-hour incubation with biotin-phenol prior to activation of the intracellular biotin-labeling reaction for 2 minutes with H_2_O_2_ (with the exception of the control, as outlined in Supplemental Figure 2, A and B). Cells were then subjected to nuclear-cytoplasmic fractionation prior to anti-biotin immunoblotting. Reassuringly, we found that APEX2-AR-V7 selectively biotinylated nuclear proteins only in the presence of biotin-phenol and the APEX2 peroxidase activator H_2_O_2_ (Figure 1C), the magnitude of which was more pronounced after 2-hour biotin-phenol treatment (Supplemental Figure 2C). Importantly, the levels of protein biotinylation were not affected by 4 Gy IR treatment. Given that we detected several contaminating endogenously biotinylated proteins in the cytoplasmic fractions of CWR22Rv1-AR-EK cells, but not in the nuclear compartment (Supplemental Figure 2D), we chose to focus our study on nuclear AR-V7 interactions.

### Proximity biotinylation detects multiple steady-state and post-IR AR-V7–interacting proteins.

Having established that APEX2-AR-V7 was nuclear, enriched at canonical target genes, and peroxidase active, we conducted 3 independent FLAG-APEX2-AR-V7 biotin-labeling experiments in CWR22Rv1-AR-EK cells and enriched nuclear biotinylated proteins using streptavidin immunoprecipitation from nuclear extracts prior to mass spectrometry analysis. The resultant Thermo RAW files were analyzed using MaxQuant to identify proteins and provide an intensity-based absolute quantification (iBAQ) value that represents individual protein abundance. The protein lists were then processed to omit common contaminants identified in the control experiments and to enrich for proteins that were identified by 2 or more unique peptides. As expected, we identified considerably more proteins in the –IR and +IR experimental arms (+H_2_O_2_) compared with the control (–H_2_O_2_) (Supplemental Figure 3A), and both heatmap- and principal component analysis–based clustering of the data showed robust separation of the control and –IR/+IR samples (Supplemental Figure 3, B and C). Proteins that had ≥2 unique peptides and were identified in ≥2 replicates and were plotted using their mean relative iBAQ (riBAQ) values (Figure 1D and Supplemental Table 4 for raw proteomics data), with 435 and 467 proteins detected in –IR and +IR treatments, respectively, and an 88% overlap between the 2 experimental arms (Supplemental Figure 3D; see Supplemental Table 5 for protein lists).

Reassuringly, AR was the fourth most abundant protein in both the –IR and +IR arms, and known AR interacting proteins were identified, including PARP1 (43) and FUS (44). Importantly, we detected DNA-PKcs as an AR-V7–interacting protein both in steady-state and after irradiation, which is consistent with what was previously observed for ARv567es. Furthermore, Ku70 (XRCC6) and Ku80 (XRCC5) were also identified as AR-V7–interacting proteins in both treatment conditions, suggesting that the DNA-PK holoenzyme regulates AR-V7, and other AR-Vs, irrespective of cell state. Gene ontology analysis of the –IR and +IR AR-V7–interacting protein lists indicated largely similar biological processes involving RNA splicing, metabolic processing, and transcription, which was not surprising given the degree of overlap between the 2 interactomes (Supplemental Tables 6 and 7). We next compared mean riBAQ values between –IR and +IR experimental arms to investigate flux to the AR-V7 interactome upon DNA damage. We identified 73 proteins whose abundance increased by 1.5-fold, including Ku80 (IR-Up; Figure 1E and Supplemental Table 8), and 62 proteins demonstrating reduced abundance in response to IR (IR-Down; Supplemental Table 8). To rule out potential IR-induced transcriptional changes contributing to the differences in AR-V7 interactomes between –IR and +IR, gene expression of a number of AR-V targets and interactors were analyzed by qRT-PCR. Reassuringly, no significant difference in gene expression was observed between the 2 experimental arms (Supplemental Figure 3E). As before, gene ontology analysis of IR-Up and IR-Down protein lists indicated involvement in splicing, nucleic acid binding, and transcriptional coregulation (Figure 1F and Supplemental Table 9). Finally, we compared our total list of AR-V interacting proteins with AR-FL, ARv567es, and the recently published DNA-PKcs interactomes (34, 41, 45). We detected 21% and 12% overlaps, respectively, between individual FL-AR and ARv567es interactomes with our list of AR-V7–interacting proteins, which included transcriptional coregulators NCOR1, TLE3, and MBD2 (Supplemental Figure 4, A and B); the low number likely reflected differences in experimental approach and cell lines used. Interestingly, a 37% overlap between proteins that interacted with DNA-PKcs and those binding AR-V7 was observed, with RNA metabolism and splicing being key gene ontology processes for the shared interacting proteins (Supplemental Figure 4C).

### DNA-PK inhibitors diminish growth of AR-V–positive PC.

**Figure 2:**
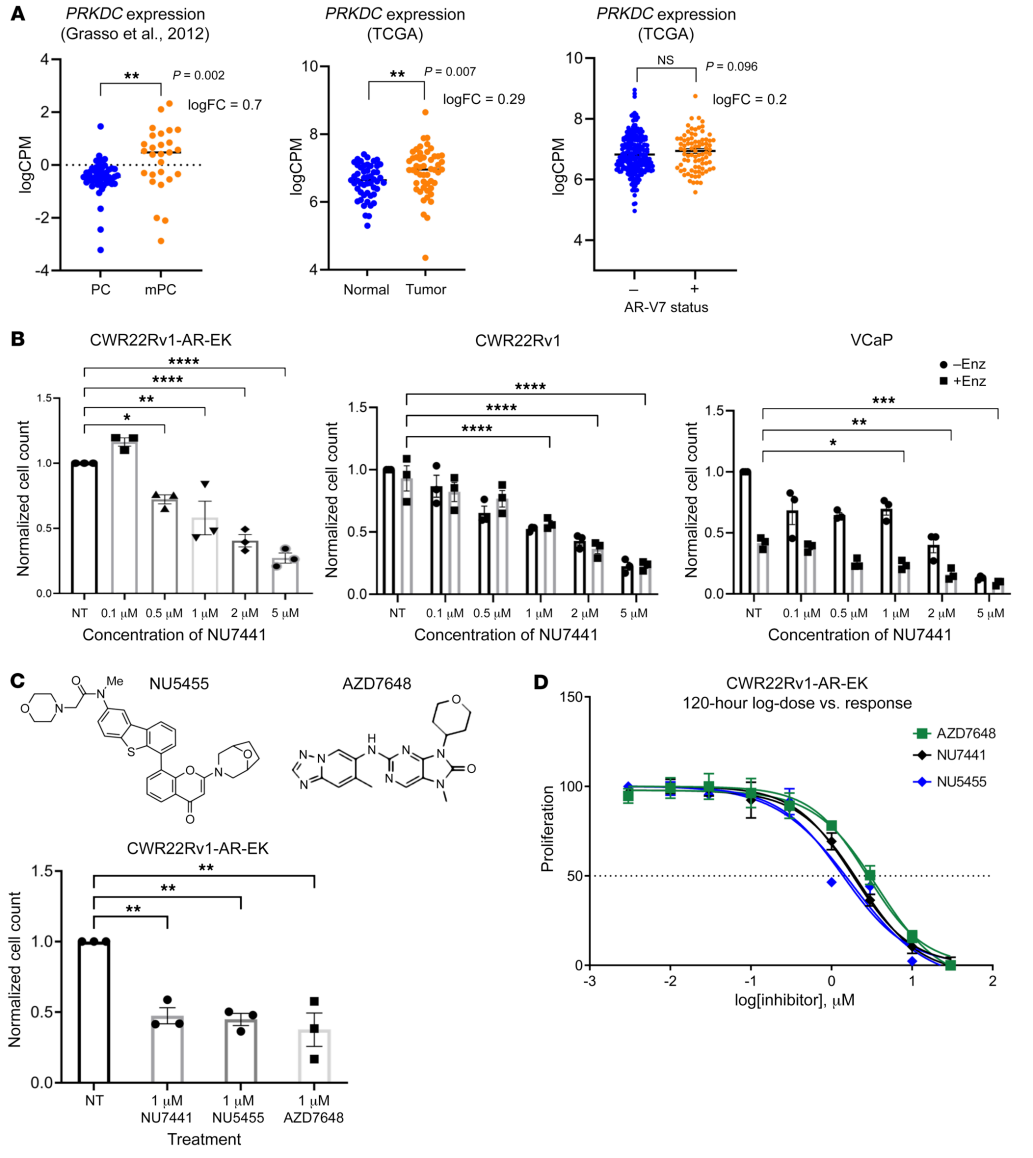
DNA-PKcs inhibition represses growth of AR-V–expressing PC cell lines. (**A**) The TCGA data set was analyzed to compare *PRKDC* expression in matched normal and tumor samples (*n* = 51) and in localized (*n* = 49) and metastatic (*n* = 27) PC from a publicly available microarray data set (Grasso et al., ref. 10). **P < 0.01. (**B**) CWR22Rv1-AR-EK cells grown in serum-containing media and CWR22Rv1 and VCaP cells grown in steroid-depleted media supplemented with 10 M enzalutamide (Enz) were treated with increasing concentrations of NU7441 for 96 hours prior to cell count. Data were normalized to the untreated (NT) control arm (–Enz/–DHT group) and are representative of 3 independent repeats ± SEM. One-way ANOVA using Bonferroni’s post hoc analysis was used to determine the statistical significance for CWR22Rv1-AR-EK and 2-way ANOVA was used for CWR22Rv1, LNCaP, and VCaP cells. *P < 0.05, **P < 0.01, ***P < 0.001. (**C**) Representative structures of DNA-PKcs inhibitors NU5455 and AZD7648 are shown adjacent to cell count data from CWR22Rv1-AR-EK cells treated with 1 mM NU7441, NU5455, and AZD7648 for 24 hours. Data represent an average of 3 repeats ± SEM (*P < 0.05, **P < 0.01). (**D**) CWR22Rv1-AR-EK cells were treated with increasing concentrations of AZD7648, NU7441, and NU5455 for 120 hours before harvesting for an SRB proliferation assay. Data are shown as the mean ± SEM across 3 independent repeats that included 3 technical replicates for each experimental arm.

Having demonstrated that AR-V7 interacts with DNA-PKcs, which is consistent with previous reports of an ARv567es-DNA-PKcs interaction (39), and that both proteins have common binding partners, we hypothesized that DNA-PKcs is a key regulator of AR-V activity in advanced PC. Upregulated *PRKDC* (DNA-PKcs) expression in primary and metastatic disease has been previously reported, which we confirmed by analyzing 2 additional data sets from the The Cancer Genome Atlas (TCGA) (46) and Grasso et al. (10) (Figure 2A). Given that AR-V expression is elevated in advanced PC, we investigated whether *PRKDC* expression was enhanced in AR-V7–positive patients compared with their AR-V7–negative counterparts. Although a trend of elevated DNA-PKcs mRNA was observed in AR-V7–expressing patients, no statistical difference between variant-positive and -negative patients was found (Figure 2A). Importantly, however, treatment of the AR-FL–negative, AR-V–positive CWR22Rv1-AR-EK cell line with the first-generation DNA-PKcs inhibitor (DNA-PKI), NU7441, markedly diminished cell proliferation (Figure 2B and Supplemental Figure 5A) to levels equivalent to AR-V depletion (16), suggesting that DNA-PKcs blockade reduces growth of this cell line, in part, by attenuating AR-V signaling. These findings were mirrored in NU7441-treated FL-AR and AR-V–positive cell lines CWR22Rv1 and VCaP grown in the presence and absence of enzalutamide (Figure 2B and Supplemental Figure 5A), further supporting the concept that compromised AR signaling in response to DNA-PKcs inhibition contributes to diminished cell growth, consistent with previous reports (47). Two additional, more selective DNA-PKIs, NU5455 and AZD7648, also caused a significant reduction in CWR22Rv1-AR-EK proliferation (Figure 2C and Supplemental Figure 5B), with similar GI_50_ doses defined for the 3 compounds (Figure 2D and Supplemental Figure 5C). Subsequent cell cycle analysis indicated that both NU7441 and NU5455 modestly elevated the apoptotic sub-G_1_ population at the expense of S phase (Supplemental Figure 6).

### DNA-PKcs is a bona fide AR-V coregulator.

**Figure 3:**
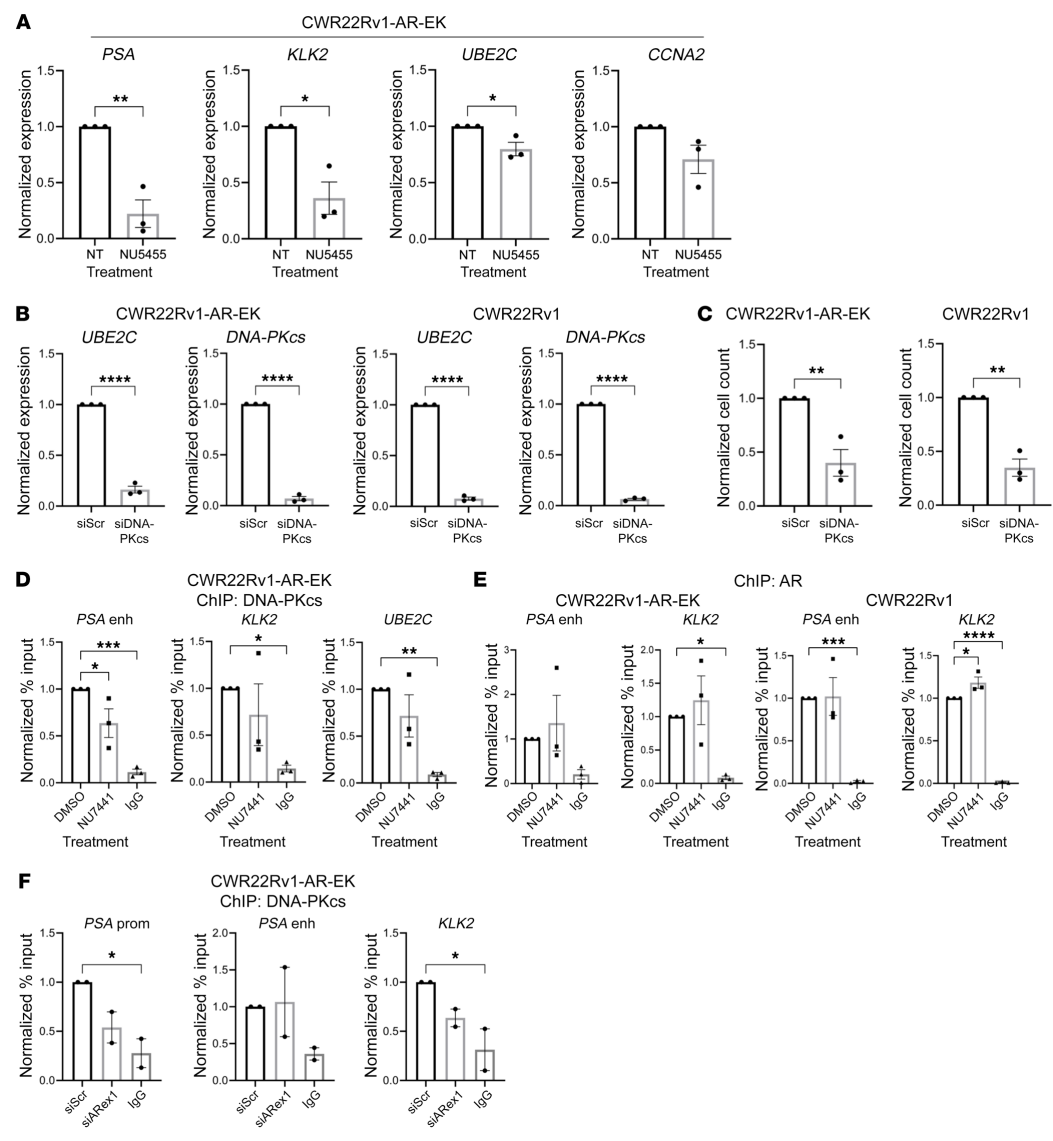
DNA-PKcs is a transcriptional coregulator of AR-Vs. (**A**) CWR22Rv1-AR-EK cells were cultured in serum-containing media for 48 hours and then treated with 1 μM NU5455 for 24 hours prior to qRT-PCR. Data were normalized to the DMSO treatment arm for each target gene and are representative of 3 independent repeats ± SEM. One-way ANOVA using Bonferroni’s post hoc analysis was used to determine the statistical significance. *P < 0.05, **P < 0.01. (**B**) CWR22Rv1-AR-EK and CWR22Rv1 cells were transfected with either siScr or siDNA-PK for 72 hours prior to qRT-PCR. Data represent the mean of 3 repeats ± SEM. An unpaired 2-tailed *t* test was used to determine statistical significance. ***P < 0.001, ****P < 0.0001. (**C**) CWR22Rv1-AR-EK cells cultured in serum-containing media and CWR22Rv1 cells cultured in steroid-depleted conditions were transfected with either siScr or siDNA-PKcs for 96 hours prior to cell count. Data are representative of 3 independent repeats ± SEM. An unpaired 2-tailed *t* test was used to determine the statistical significance. **P < 0.01. (**D** and **E**) CWR22Rv1-AR-EK cells grown in serum-containing media were treated with 1 μM NU7441 prior to ChIP using (**D**) anti-DNA-PKcs, (**E**) anti-AR, and isotype control (IgG) antibodies. ChIP-qPCR readouts represent the normalized percentage input to the control of 3 independent experiments incorporating 1-way ANOVA using Bonferroni’s post hoc analysis to determine the statistical significance. *P < 0.05, **P < 0.01, ***P < 0.001, ****P < 0.0001. (**F**) CWR22Rv1-AR-EK cells grown in serum-containing media were transfected with either scrambled control (siScr) or AR exon 1-targeting (siARex1) siRNAs and incubated for 72 hours before ChIP using DNA-PKcs and isotype control (IgG) antibodies. Data shown represent the normalized percentage input to the control and represents 2 independent repeats. One-way ANOVA using Bonferroni’s post hoc analysis was used to determine the statistical significance. *P < 0.05.

To establish if the antiproliferative effects of DNA-PKIs were a consequence of compromised AR signaling, CWR22Rv1 derivative and VCaP cell lines were treated with 1 mM NU5455, AZD7648, or a dose range of NU7441 for 24 hours prior to qRT-PCR analysis of canonical AR/AR-V target genes. All 3 DNA-PKIs selectively diminished expression of *PSA*, *KLK2*, *UBE2C*, and *CCNA2* in CWR22Rv1-AR-EK cells (Figure 3A and Supplemental Figure 7, A and B), which are directly bound by AR-Vs (16), an effect largely consistent in CWR22Rv1 and VCaP cells treated with NU7441 and NU5455 (Supplemental Figure 8). We next assessed whether DNA-PKcs depletion using a commercially available 4-siRNA pool (Dharmacon SmartPool; DNA-PKcs-SP) would mimic the effect of DNA-PKcs blockade on AR-V activity and PC cell growth. Surprisingly, DNA-PKcs knockdown failed to effect AR-target gene expression (Supplemental Figure 9) and growth of CWR22Rv1-AR-EK and CWR22Rv1 cells (Supplemental Figure 10), even though depletion of DNA-PKcs was evident. We subsequently tested the effect of the deconvoluted siRNA pool (individual siDNA-PK1-4) on PC cell growth and expression of canonical AR-V target genes *CCNA2* and *UBE2C* to assess if potential off-target effects of one or more DNA-PKcs–targeting oligonucleotides caused the inconsistencies between our DNA-PKI and knockdown readouts. As shown in Supplemental Figures 11 and 12, contrary to individual siDNA-PKs 2-4, siDNA-PK 1 failed to reduce both growth of CWR22Rv1 derivatives and AR-V–target gene expression, suggesting that efficacy of the SmartPool may have been compromised by siDNA-PK 1. Therefore, using a custom siRNA pool consisting of siDNA-PK 2-4 (siDNA-PKcs) to effectively deplete DNA-PKcs, we subsequently showed robust depletion of *UBE2C* (Figure 3B), *CCNA2*, *TMPRSS2*, *FKBP5*, and *CDC20* (Supplemental Figure 13) and PC cell growth (Figure 3, B and C) as a consequence of G_1_ arrest (Supplemental Figure 14A). Furthermore, quantification of H2AX after 1–24 hours of DNA-PKcs inhibition revealed no significant elevation of steady-state DNA damage, supporting the concept that compromised DNA-PKcs activity diminishes AR-V activity that, in part, compromises cell cycle progression, as evidenced by depleted AR target gene expression, as opposed to checkpoint activation in response to DNA-PKcs blockade (Supplemental Figure 14B). Together, our findings support the hypothesis that DNA-PKcs is a transcriptional coregulator of AR-Vs in addition to its characterized role in FL-AR coregulation (38).

To explore this further, we investigated whether DNA-PKcs was recruited to AR-V target genes using ChIP. Consistent with findings from C4-2 cells showing that DNA-PKcs was recruited to AR-FL–regulated genes (38), we found robust enrichment of the kinase on *cis*-regulatory elements of a number of AR-V–target genes, including *KLK3*/*PSA*, *KLK2*, *UBE2C*, and *TMPRSS2*, in CWR22Rv1-AR-EK and VCaP cells (Figure 3D and Supplemental Figure 15A), that was refractory to DNA-PKI NU7441 (Figure 3D). Of interest was the finding that topoisomerase I (TOP1) was part of the AR-V interactome (Supplemental Table 4), suggesting it may be involved in recruiting components of the DDR, including DNA-PKcs, during the process of AR-V–mediated transcription akin to that observed for AR-FL (48). However, siRNA depletion of TOP1 did not effect enrichment of DNA-PKcs to canonical AR target genes in CWR22Rv1 cells, indicating that kinase recruitment is independent of TOP1 activity (Supplemental Figure 15, B and C). Interestingly, while DNA-PKcs blockade or knockdown failed to affect AR enrichment at the sites tested in CWR22Rv1 derivatives (Figure 3E) and VCaP cells (Supplemental Figure 15A), AR-V knockdown in CWR22Rv1-AR-EK cells modestly reduced DNA-PKcs chromatin binding at the *KLK3*/*PSA* and *KLK2* genes (Figure 3F), suggesting that AR *cis*-regulatory element binding is independent of DNA-PKcs catalytic activity but is required to facilitate DNA-PKcs chromatin recruitment.

### Comparing global impact of DNA-PKcs blockade and knockdown in AR-V–positive PC.

**Figure 4:**
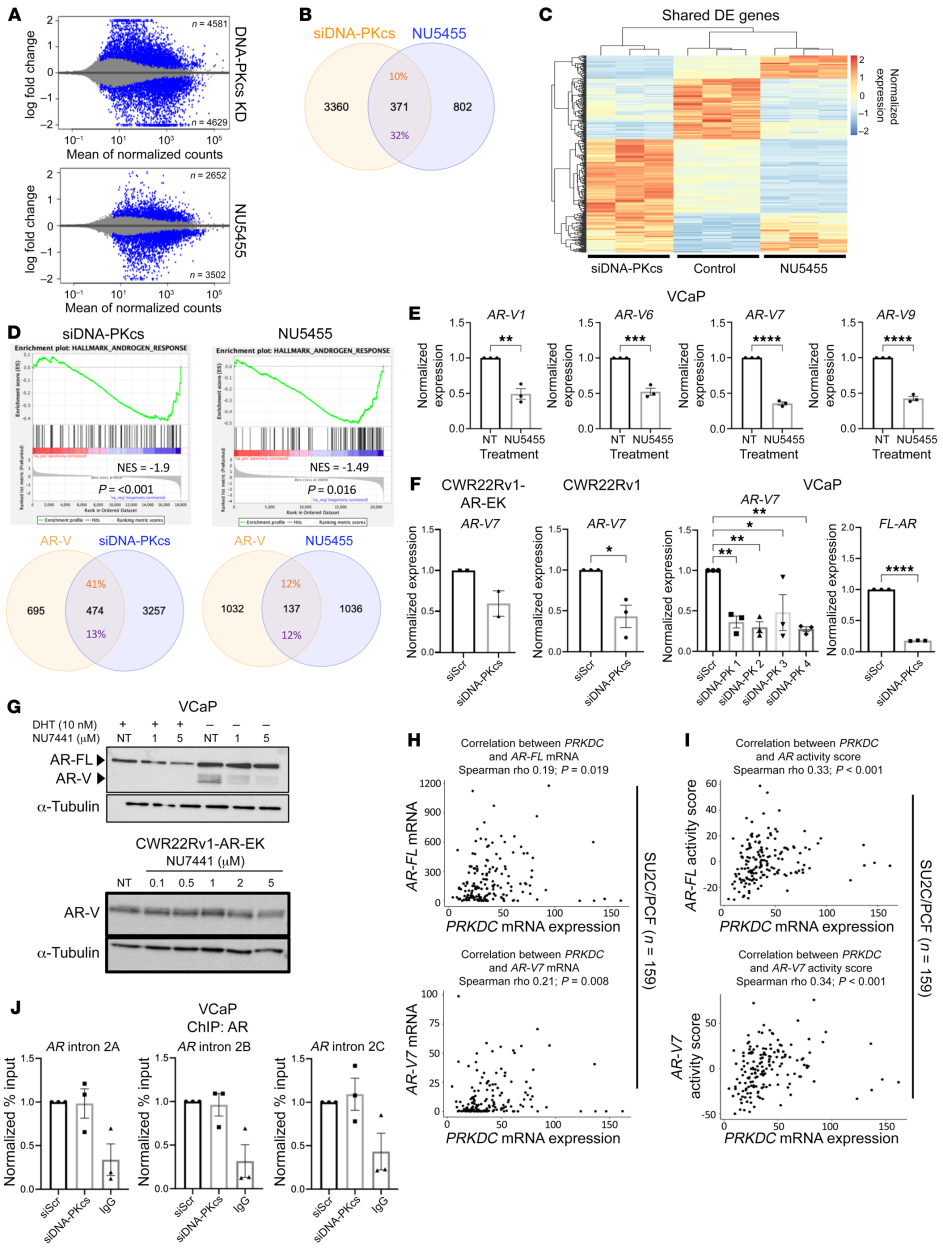
DNA-PKcs blockade and knockdown markedly affects the AR-V transcriptome. (**A**) MA plot (log fold change [M] versus mean of normalised counts [A]) showing the number of up- and downregulated genes in response to DNA-PKcs knockdown and inhibition with NU5455 (blue represents statistically significant differentially expressed genes [DEGs], *P* adjusted < 0.05). (**B**) Venn diagram indicating the percentage overlap of DEGs (*P* < 0.05, fold change ± 1.5) between DNA-PKcs depletion (siDNA-PKcs) and inhibition (NU5455). (**C**) Heatmap of overlapping DEGs between DNA-PKcs knockdown and inhibition compared with control. (**D**) Unfiltered DEG lists from NU5455 and siDNA-PKcs treatment were compared with the “androgen response hallmark” gene lists using GSEA. Venn diagrams show the percentage overlap between AR-V transcriptome (Kounatidou et al., ref. 16) and DNA-PKcs knockdown or inhibition DEGs. (**E**) VCaP cells were treated for 24 hours with 1 μM NU5455 with and without DHT before RT-qPCR analysis. Data represent the mean of 3 repeats ± SEM. An unpaired 2-tailed *t* test was used to determine the statistical significance. *P < 0.05, **P < 0.01, ***P < 0.001, ****P < 0.0001. (**F**) Cells were transfected with oligonucleotides targeting DNA-PKcs or a scrambled (siScr) control for 72 hours prior to RT-QPCR, as in **E**. (**G**) AR immunoblotting of VCaP and CWR22Rv1-AR-EK cells grown in increasing doses of NU7441 or vehicle control (NT) for 24 hours. (**H** and **I**) Association of *DNA-PKcs* (*PRKDC*) mRNA levels with (**H**) *AR* and *AR-V7* mRNA levels and (**I**) AR and AR-V7 activity scores in SU2C/PCF (*n* = 159) CRPC transcriptomes. *r* and *P* values were calculated using Spearman’s correlation. (**J**) VCaP cells grown in steroid-depleted media were transfected with DNA-PKcs–targeting (siDNA-PKcs) or control scrambled siRNA (siScr) for 72 hours prior ChIP using AR or isotype control (IgG) antibodies. Data shown represent the normalized fold enrichment to siScr control and are the mean of 3 independent repeats.

To study the global transcriptional involvement of DNA-PKcs in AR-V–positive PC, RNA-Seq was performed in CWR22Rv1-AR-EK cells either depleted of DNA-PKcs for 72 hours (using our bespoke siDNA-PKcs pool) or treated with vehicle, 1 μM NU7441, NU5455, or AZD7648 for 24 hours (Supplemental Figure 16A). Validation of our samples before and after sequencing, respectively, confirmed selective downregulation of DNA-PKcs in the siDNA-PKcs samples (Supplemental Figure 16B) and clear overlaps between the biological replicates and separation of each experimental arm according to treatment (Supplemental Figure 16C). Considerable variation in the number of significantly differentially expressed genes (DEGs; >1.5-fold cut-off) was observed, with 44, 27, 1,195, and 3,827 altered in response to AZD7648, NU7441, NU5455, and DNA-PKcs knockdown, respectively (Figure 4A and Supplemental Figure 17, A and B; see Supplemental Tables 10–13). The more selective NU5455 compound demonstrated the highest effect on global gene expression and had 89% and 34% overlaps with NU7441 and AZD7648 DEGs, respectively (Supplemental Figure 16C). *TWIST1* and *PDK4* were identified as the only commonly altered genes among the 3 DNA-PKIs. Interestingly, *PDK4* was upregulated and *TWIST1* was downregulated across the 3 data sets, providing confidence that these are genuine DNA-PKcs–regulated genes in CWR22Rv1-AR-EK cells. Gene set enrichment analysis (GSEA) identified 3 shared DNA-PKI–altered pathways among NU7441, NU5455, and AZD7648 gene sets, “p53,” “cell cycle,” and “DNA replication” (Supplemental Figure 17D). We next compared DEGs from our NU7441-treated CWR22Rv1-AR-EK data set to those obtained from 2 separate studies examining the effect of NU7441 treatment on C4-2 cells (38, 47). Surprisingly, we found only 1 common gene between the DNA-PKI–treated CWR22Rv1-AR-EK and C4-2 cells and only 2 overlapping genes between the 2 C4-2 studies using our analysis pipeline. These findings likely reflect the effect of NU7441 in 2 distinct cell backgrounds and variation in utilizing microarray (38) and RNA-Seq (47) for transcriptomics analyses (Supplemental Figure 18A).

Given that NU5455 resulted in the greatest number of DEGs of the 3 tested DNA-PKIs, we next compared global transcriptional effects of NU5455 and DNA-PKcs depletion in our AR-V–expressing only CWR22Rv1-AR-EK cell line to define catalytic versus noncatalytic dependencies of DNA-PKcs in transcriptional regulation. As shown in Figure 4B, 371 overlapping DEGs were identified between DNA-PKcs blockade and knockdown. Distinct clustering of up- and downregulated genes was evident when compared with control (Figure 4C and Supplemental Table 14A for shared gene list). Consistent with our data describing DNA-PKcs as a regulator of AR-Vs, GSEA of NU5455 and DNA-PKcs knockdown gene lists indicated robust negative enrichment of “hallmark androgen response” (Figure 4D), confirming DNA-PKcs–mediated regulation of AR-Vs. Interestingly, while NU5455 DEGs showed only a 12% overlap with our previously defined AR-V transcriptome (16), which is similar to that of AZD7648- and NU7441-treated samples (Supplemental Figure 18B and Supplemental Table 14B), we found a markedly elevated 41% overlap between the DNA-PKcs knockdown and AR-V–regulated gene sets (Figure 4D, Supplemental Figure 19, and Supplemental Table 14C). Furthermore, we found DNA-PKcs inhibition and depletion also diminished abundance of multiple AR isoform transcripts, including *AR-V7*, as well as *AR-V1*, -*V6* and -*V9* and *FL-AR* in VCaP, CWR22Rv1-AR-EK and CWR22Rv1 cells (Figure 4, E and F) which translated to reduced AR-V and FL-AR protein in the tested PC cell lines (Figure 4G). Next, to evaluate the clinical relevance of these findings, we interrogated 2 independent CRPC patient transcriptome cohorts (Stand Up to Cancer/Prostate Cancer Foundation [SU2C/PCF], *n* = 159; ICR/Royal Marsden Hospital [ICR/RMH], *n* = 95) to determine whether DNA-PKcs (PRKDC) mRNA expression was associated with *AR/AR-V7* mRNA and AR/AR-V7 activity scores (Supplemental Figure 20A) (20, 49, 50). These analyses demonstrated that *PRKDC* mRNA expression significantly positively associated with *AR* mRNA expression in both cohorts (SU2C/PCF, *r* = 0.19, *P* = 0.019; ICR/RMH, *r* = 0.31, *P* = 0.003) and with *AR-V7* mRNA expression in the SU2C/PCF cohort (*r* = 0.21, *P* = 0.008) but not the ICR/RMH cohort (*r* = 0.19, *P* = 0.067) (Figure 4H and Supplemental Figure 20B). Interestingly, *PRKDC* mRNA expression demonstrated a stronger, and more significant, positive association with AR (SU2C/PCF, *r* = 0.33, *P* < 0.001; ICR/RMH, *r* = 0.46, *P* < 0.001) and AR-V7 (SU2C/PCF, *r* = 0.34, *P* < 0.001; ICR/RMH, *r* = 0.49, *P* < 0.001) activity scores in both cohorts studied (Figure 4I and Supplemental Figure 20C). Together, these data demonstrate that, for the first time to our knowledge, DNA-PKcs control AR-V signaling potentially at multiple levels involving regulation of *AR-V* mRNA production and by conventional coregulation of chromatin-bound AR-V transcriptional activity.

### DNA-PKcs regulates AR-V splicing in PC cells.

Having demonstrated that inhibition and knockdown of DNA-PKcs reduced *AR-V* (and *FL-AR*) mRNA levels in PC cells, we next sought to define the mechanism(s) of DNA-PKcs–mediated regulation of *AR-V* expression. First, we examined whether DNA-PKcs controlled deposition of AR at the downstream repressive element (DRE) in intron 2 of the *AR* gene, which has been shown to negatively regulate AR expression in PC cells (51). We hypothesized that DNA-PKcs depletion would enhance AR enrichment at this locus and thus diminish *AR-V* expression. In contrast, however, we found that AR binding to the intron 2 DRE in VCaP cells was unaffected by DNA-PKcs knockdown (Figure 4J); hence, the reduction in AR-V levels upon kinase knockdown and inhibition is independent of AR-DRE transactions.

**Figure 5:**
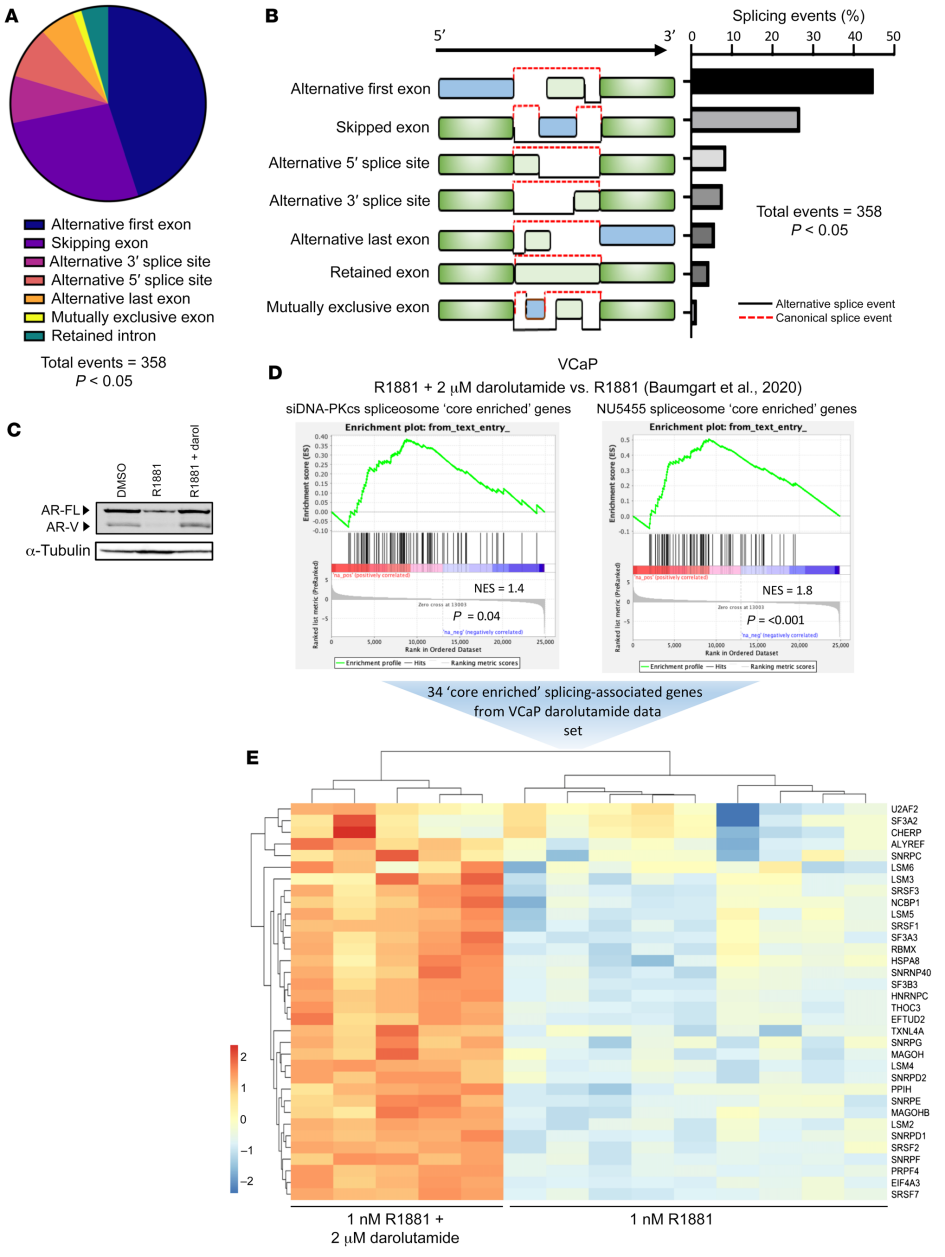
DNA-PKcs regulates a splicing-associated gene signature. (**A**) RNA-Seq data derived from CWR22Rv1-AR-EK cells depleted of DNA-PKcs was analyzed for differential splicing activity using SUPPA2 inbuilt statistical test (ref. 65). Events that passed a *P* value cut off of <0.05 were plotted in the pie chart. (**B**) Diagrammatic representation and quantification of the statistically significant splicing alterations detected in response to DNA-PKcs depletion, as determined using SUPPA2 inbuilt statistical test (ref 65). (**C**) Upregulation of AR-V7 in response to darolutamide was validated by Western blotting using an anti-AR-V7 antibody. (**D**) Differentially expressed splicing-associated genes from DNA-PKcs depleted and NU5455-treated cells were analyzed by GSEA using a darolutamide-responsive gene set (Baumgart et al., ref. 54) to identify splicing factor expression correlating with *AR-V7* synthesis. (**E**) 34 splicing-associated genes were found to be upregulated in response to darolutamide and are shown in the heatmap.

Interestingly, inspection of GSEA data from DNA-PKcs knockdown and NU5455-/NU7441-treated cells indicated significant negative enrichment of the “spliceosome” gene set, suggesting that splicing activity is perturbed in cells with compromised DNA-PKcs activity (Supplemental Figure 21, A and B; see Supplemental Tables 15–17 for altered splicing-related genes). This was confirmed by evaluating global splicing changes upon DNA-PKcs depletion; we detected over 11,000 alternative splicing events, 358 of which were statistically significant, with alternative first exon and exon skipping being the most predominant changes (Figure 5, A and B, and Supplemental Figure 22A). Interestingly, several of the spliceosome genes common to DNA-PKcs knockdown (Supplemental Figure 22B) and NU5455 treatment (Supplemental Table 18), such as *SF3B3*, *U2AF2* and *SRSF1*, have been previously implicated in AR gene splicing (52, 53), suggesting that DNA-PKcs knockdown and inactivation reduce AR-V levels by potentially preventing splicing of nascent *AR* transcripts into mature *AR-V*–encoding mRNA.

To explore this further, we cross-referenced our negatively enriched spliceosome gene sets from both NU5455 and siDNA-PKcs treatments with DEGs upregulated in response to the next-generation anti-androgen darolutamide in VCaP cells (54), a treatment that upregulates production of FL-AR and AR-Vs (Figure 5C). We reasoned that genes from the spliceosome gene set that are upregulated in response to darolutamide (in which AR/AR-V expression is high), and show concurrent downregulation in response to DNA-PKcs inhibition/knockdown, may be important for DNA-PKcs–regulated *AR* isoform splicing. As shown in Figure 5D, GSEA demonstrated a positive and statistically significant enrichment of darolutamide-responsive genes in the negatively enriched spliceosome gene sets from NU5455 (normalized enrichment score [NES] = 1.4, *P* = 0.04) and siDNA-PKcs (NES = 1.8, *P* = <0.001) treatment (Supplemental Table 19). From this, we identified 34 common splicing-associated genes, including *PRPF4* and *LSM5*, whose expression was reduced by compromised DNA-PKcs activity and correlated with *AR-V* expression in darolutamide-treated PC cells (Figure 5E and Supplemental Figure 22, C and D). Of these splicing-associated genes, 10 demonstrated significantly elevated expression in TCGA PC samples compared with normal samples (Supplemental Figure 22E) and, for *RBMX*, upregulated transcript levels in higher grade disease (Supplemental Figure 22, F and G).

### RBMX is a DNA-PKcs regulated gene critical for AR-V splicing.

**Figure 6:**
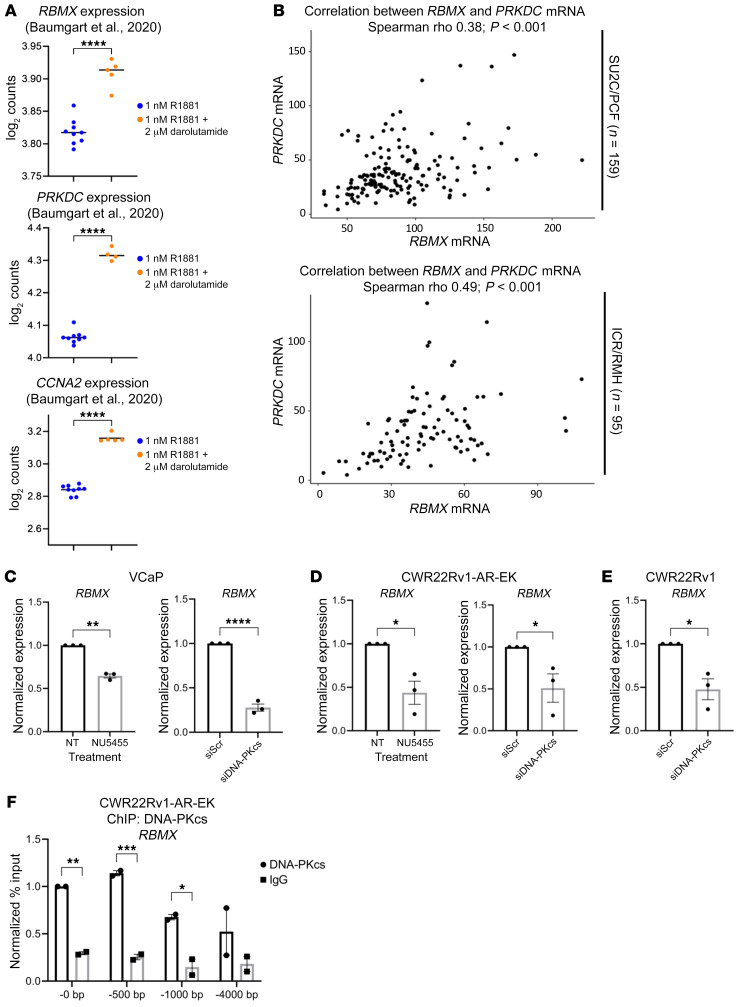
RBMX is a bona fide DNA-PKcs–regulated gene. (**A**) Graphical representations of *RBMX*, *DNA-PKcs* (*PRKDC*), and *CCNA2* expression from Baumgart et al. (54). Statistics were determined with limma and GEO2R. ****P < 0.0001. (**B**) Association of *DNA-PKcs* (*PRKDC*) mRNA levels with *RBMX* mRNA levels in SU2C/PCF (*n* = 159) and ICR/RMH (*n* = 95) CRPC transcriptomes. *r* and *P* values are shown and were calculated using Spearman’s correlation. (**C**) VCaP, (**D**) CWR22Rv1-AR-EK, and (**E**) CWR22Rv1 cells were either reverse transfected with siScr/siDNA-PKcs for 72 hours or treated for 24 hours with 1 μM DNA-PKcs inhibitors (NU5455) prior to RT-qPCR analysis of RBMX expression. An unpaired 2-tailed *t* test was used to determine the statistical significance from 3 independent experiments. *P < 0.05, **P < 0.01, ****P < 0.0001. (**F**) CWR22Rv1-AR-EK cells grown in serum-containing media were subject to ChIP using either DNA-PKcs or isotype control (IgG) antibodies prior to qPCR analysis to assess DNA-PKcs enrichment at and upstream of the RBMX transcriptional start site (0, –500, –1,000, and –4,000 bp) Data shown represent the normalized percentage input to the DNA-PKcs ChIP at the –0 bp site and represent 2 independent repeats. Two-way ANOVA using Šídák’s multiple comparisons test was used to determine the statistical significance. *P < 0.05, **P < 0.01, ***P < 0.001.

Further interrogation of the Baumgart et al. transcriptomics data (54) indicated a correlation between *DNA-PKcs* and *RBMX* mRNA that was coincident with elevated expression of AR target genes, including *CCNA2* (Figure 6A). Interestingly, *DNA-PKcs* and *RBMX* transcripts were elevated in VCaP cells in response to antiproliferative doses of darolutamide, indicating that expression of *DNA-PKcs* and *RBMX* mRNAs is independent of cell proliferation in this model (54). This correlation was also evident across several clinical studies, including SU2C/PCF (49) and ICR/RMH (Figure 6B) as well as MSKCC (55) and TCGA (Supplemental Figure 23), supporting the concept that *RBMX* is a DNA-PKcs–regulated gene. To test this further, we subjected PC cells to either DNA-PKcs blockade or knockdown for 24 and 72 hours prior to *RBMX* transcript profiling, respectively. As shown in Figure 6, C–E, NU5455 and siDNA-PKcs treatment significantly diminished *RBMX* expression. Furthermore, we detected significant enrichment of DNA-PKcs at *cis*-regulatory elements proximal to the transcriptional start-site of the *RBMX* gene, but not at a site 4 kb upstream, confirming that *RBMX* is a bona fide DNA-PKcs–regulated gene (Figure 6F).

**Figure 7:**
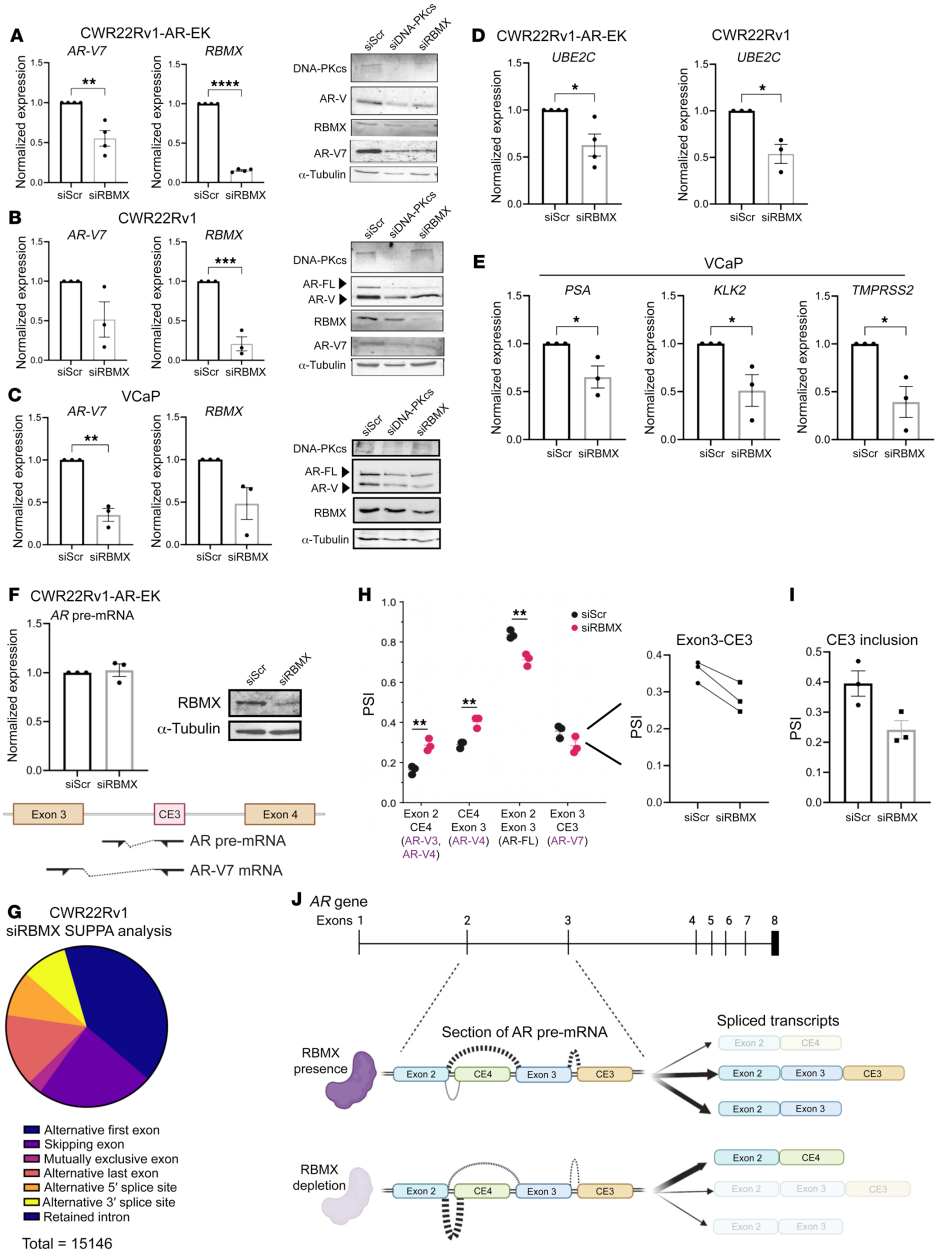
RBMX regulates AR-V synthesis in prostate cancer. (**A**) CWR22Rv1-AR-EK, (**B**) CWR22Rv1, and (**C**) VCaP cells grown in serum-containing and steroid-depleted media, respectively, were transfected with RBMX (siRBMX) or scrambled control (siScr) siRNAs for 72 hours prior to *AR-V7* and *RBMX* transcript analysis using RT-qPCR. Data represent the mean of 3 repeats ± SEM. An unpaired 2-tailed *t* test was used to determine the statistical significance. *P < 0.05, **P < 0.01, ***P < 0.001, ****P < 0.0001. In parallel, AR, AR-V7, DNA-PKcs, and RBMX protein levels were analyzed by Western blot in cells depleted of DNA-PKcs and RBMX for 72 hours. (**D**) CWR22Rv1-AR-EK and CWR22Rv1 and (**E**) VCaP cells were depleted of RBMX, as in **A**–**C**, and canonical AR-V–target gene expression was analyzed by qRT-PCR. Data represent the mean of 3 repeats ± SEM. An unpaired 2-tailed *t* test was used to determine the statistical significance. *P < 0.05. (**F**) CWR22Rv1-AR-EK cells depleted of RBMX for 72 hours were subject to qRT-PCR analysis to assess unspliced, pre-mRNA *AR* transcript abundance compared with scrambled siRNA (siScr) control. Representative Western analysis is shown to demonstrate successful RBMX knockdown. (**G**) RNA-Seq data derived from CWR22Rv1 cells depleted of RBMX was analyzed for differential splicing activity using SUPPA2. Events that passed a *P* value cut off of < 0.05 were plotted in the pie chart. (**H** and **I**) Altered exon composition of distinct AR transcripts as calculated by investigating relative exon inclusion (PSI) for all junctions measured using (**H**) hisat2 and (**I**) SUPPA2. (**J**) Diagrammatic representation of exon inclusion dynamics across exon 2 to cryptic exon 3 (CE3) in steady-state and in response to RBMX knockdown.

We next depleted CWR22Rv1 derivative and VCaP cell lines of either DNA-PKcs or RBMX and assessed *AR* transcript and protein abundance by qRT-PCR and Western analyses, respectively. In all PC cell lines tested, we observed downregulated *AR-FL* and *AR-V* mRNAs, including *AR-V7*, *-V1*, *-V6*, *-V9*, which translated to reduced AR-FL and AR-V protein in response to individual knockdown of DNA-PKcs and RBMX (Figure 7, A–C, and Supplemental Figure 24A). Subsequent independent orthogonal analysis of a previously performed siRNA screen of 315 genes related to the spliceosome in CWR22Rv1 cells (56) validated that RBMX knockdown reduced AR and AR-V7 protein expression and was top hit in this assay (Supplemental Figure 24B). As such, expression of AR-V target genes *UBE2C*, and canonical androgen-regulated genes *KLK3*/*PSA*, *KLK2*, and *TMPRSS2*, was significantly reduced upon RBMX depletion (Figure 7, D and E). Crucially, we found that loss of *AR-V1*, *AR-V7*, and *AR-V9* transcripts upon DNA-PKcs blockade can be partially rescued by ectopic RBMX expression, supporting the concept that DNA-PKcs regulates *AR* transcript synthesis in an RBMX-dependent manner (Supplemental Figure 25A).

Given its role in splicing, we next sought to comprehensively define how RBMX controls *AR* transcript metabolism by monitoring turnover, synthesis, and splicing of *AR-V* and *AR-FL* mRNAs. First, in actinomycin-D time-course experiments in CWR22Rv1 cells, we found that while steady-state levels of *AR-V7* and *AR-FL* transcripts were downregulated in response to RBMX knockdown (Supplemental Figure 25B), turnover of these transcripts was not enhanced (Supplemental Figure 25C), implying that loss of *AR* mRNAs by RBMX depletion is not a consequence of elevated degradation. We next monitored *AR* pre-mRNA transcript levels by qRT-PCR, using primers complementary to CE3 and the preceding intron, and found no effect of RBMX knockdown on precursor unspliced *AR* mRNAs, suggesting that de novo transcription of the *AR* gene was not affected by RBMX depletion (Figure 7F). In addition, we found that RBMX selectively interacted with *AR* pre-mRNA, but not mature *AR-V7* transcripts (Supplemental Figure 26), further supporting the concept that RBMX is involved at a stage proceeding transcript synthesis. Furthermore, our finding that depletion of RBMX (Supplemental Figure 27A) or DNA-PKcs blockade (Supplemental Figure 27B) in CWR22Rv1 cells failed to affect production of ectopically expressed AR-V7, derived from postspliced cDNA, suggests that RBMX facilitates maturation of *AR* transcripts by regulating splicing of pre-mRNAs. To support this, we analyzed differential gene expression and exon composition of *AR* transcripts in CWR22Rv1 cells depleted of RBMX by RNA-Seq at a 100 M read depth. Validation of our samples confirmed clear overlaps among the biological replicates, separation of the control and RBMX knockdown arms (Supplemental Figure 28, A and B), and robust depletion of RBMX (Supplemental Figure 28C). 3,185 statistically significant DEGs were identified upon loss of RBMX, 38% of which overlapped with those observed in response to DNA-PKcs knockdown (Supplemental Figure 28D; see Supplemental Table 20 for full DEG list). GSEA of the RBMX knockdown gene list showed negative enrichment of “hallmark androgen response” (Supplemental Figure 28, E and F), consistent with DNA-PKcs depletion, with approximately 20% of shared RBMX- and DNA-PKcs–regulated genes overlapping with our AR-V transcriptome (Supplemental Figure 28G), suggesting that DNA-PKcs controls AR signaling, in part, by modulating expression of *RBMX*.

Evaluating global splicing changes upon RBMX knockdown, we detected over 15,000 splicing events (<–0.2 and >0.2 dPSI), 1,167 of which were statistically significant, with alternative first exon and exon skipping being the most predominant changes (Figure 7G and Supplemental Figure 29A). Crucially, using both DEXSeq and SUPPA splicing annotation tools, we observed a statistically significant increase in exon 2-cryptic exon 4–containing (CE4-containing) mRNAs at the expense of transcripts containing exon 2–exon 3 spliced junctions, which encode AR-V1, AR-V6, AR-V7, AR-V9, and AR-FL, in response to RBMX depletion (Figure 7H). A marked reduction in *AR-V7*–encoding exon 3-CE3 transcripts was also detected (Figure 7I). Although we were unable to detect an increase in exon 2–CE4–containing transcripts by qRT-PCR in cells depleted of RBMX (Supplemental Figure 29B), reassuringly the levels were not markedly reduced as observed for *AR-V1*, *AR-V6*, *AR-V7*, *AR-V9*, and *AR-FL* (Figure 7, A–C, and Supplemental Figure 24A). It is therefore evident that RBMX controls splicing decisions pertinent to the generation of *AR* mRNAs composed of appropriately spliced exons 2 and 3, which pertain to expression of AR-FL and the clinically relevant AR-Vs (Figure 7J).

**Figure 8:**
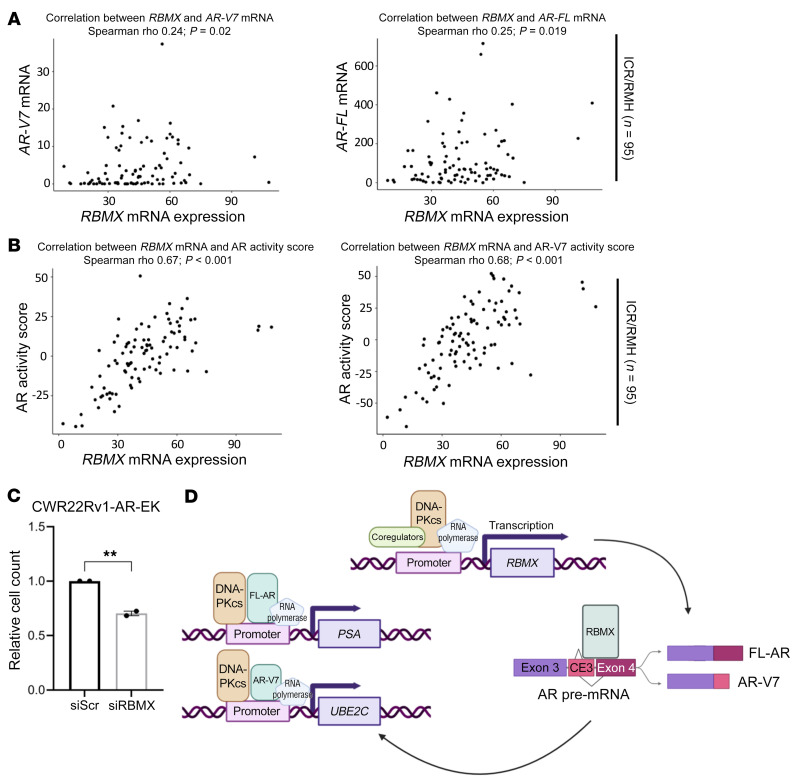
Expression of RBMX correlates with AR isoforms and activity in CRPC. (**A**) Association of *RBMX* mRNA with *AR-V7* and *AR-FL* mRNA levels and (**B**) activity scores in the ICR/RMH (*n* = 95) CRPC transcriptomes. *r* and *P* values are shown and were calculated using Spearman’s correlation. (**C**) CWR22Rv1-AR-EK cells cultured in serum-containing media were transfected with either siRBMX or siScr for 72 hours prior to cell counts analysis. Data are representative of 2 independent repeats ± SEM. An unpaired 2-tailed *t* test was used to determine the statistical significance. **P < 0.01. (**D**) Proposed mechanism of DNA-PKcs modulated AR/AR-V splicing and transcriptional activity. In normal conditions, DNA-PKcs is involved in the transcription of the RNA binding protein RBMX, which is directly involved in splicing *FL-AR* and multiple *AR-V* mRNA transcripts, including *AR-V7*. Resultant FL-AR and AR-V7 protein interacts with and is subsequently coactivated by DNA-PKcs to facilitate expression of canonical AR target genes.

To further investigate the clinical relevance of these findings, we utilized the 2 independent CRPC patient transcriptome cohorts previously described (Supplemental Figure 20A). These analyses identified that *RBMX* mRNA expression significantly positively associated with *AR* mRNA expression in the ICR/RMH cohort (*r* = 0.25, *P* = 0.019), but not the SU2C/PCF cohort (*r* = 0.01, *P* = 0.87), and with *AR-V7* mRNA expression in the ICR/RMH cohort (*r* = 0.24, *P* = 0.02), but not the SU2C/PCF cohort (r = –0.06, *P* = 0.439) (Figure 8A and Supplemental Figure 30A). Interestingly, as observed for *PRKDC*, *RBMX* mRNA expression demonstrated a stronger, and more significant, positive association with AR (SU2C/PCF, *r* = 0.24, *P* = 0.002; ICR/RMH, *r* = 0.67, *P* < 0.001) and AR-V7 (SU2C/PCF, *r* = 0.27, *P* < 0.001; ICR/RMH, *r* = 0.68, *P* < 0.001) activity scores in both cohorts (Figure 8B and Supplemental Figure 30B), supporting the concept that interplay between DNA-PKcs and RBMX contributes to *AR*/*AR-V* synthesis. Finally, we found that growth of AR-V only (FL-AR absent) CWR22Rv1-AR-EK cells was reduced upon RBMX knockdown, which, we speculate is, in part, a consequence of reduced AR isoform expression (Figure 8C). Together, our data provide evidence of a potentially new role for DNA-PKcs in controlling cellular splicing driven by a DNA-PKcs-RBMX regulatory loop that controls *AR* transcript maturation and downstream AR isoform transcriptional activity (Figure 8D), deregulation of which may lead to altered AR signaling and disease progression.

## Discussion

Although the repertoire of therapeutics to treat hormone-sensitive and CRPC has greatly expanded over the past decade, with the emergence of second-generation AR-targeting agents, such as abiraterone and enzalutamide (5, 6), their limited durability across the entire patient cohort remains a critical clinical challenge. While PC harboring *AR* gene mutations and amplification remains largely sensitive to these therapies (57), patients expressing AR-Vs are refractory to treatment (15). This represents a major problem when considering that approximately 80% of patients, whose primary treatment involves androgen deprivation therapy, progress with detectable expression of AR-Vs (20). It is imperative therefore that new tractable routes for AR-V blockade are sought to offer new treatment paradigms for this large disease cohort. To that end, considerable progress has been made over the past number of years to improve our understanding of transcriptomic and cistromic control of AR-Vs and their involvement in key cellular processes, including the DDR (16–18). Indeed, there is surmounting published evidence that AR-Vs are important for DNA repair both at the transcriptional level (16, 58) and in situ at the damaged DNA locus (39). Importantly, such endeavors have identified key vulnerabilities in AR-V signaling that could be exploited for future PC treatments, including sensitivity to PARP and HSP90 blockade (16, 59). There remain, however, key knowledge gaps in our understanding of how AR-Vs function in steady-state and during the DDR; resolving these would provide new, more effective therapeutic options for CRPC.

To that end, we undertook what we believe to be the first of its kind AR-V7 interactome study utilizing a novel APEX2 peroxidase-mediated biotinylation pipeline to provide unique protein interacting signatures of AR-V7 in the presence and absence of DNA damage. We identified 436 AR-V7–interacting proteins in normal steady-state conditions, which considerably outnumbered the 75 ARv567es binding partners detected by RIME in the R1-D567 cell line (41). Whether this is a genuine reflection of the distinct interactomes of AR-V7 and ARv567es or simply a consequence of alternative methodological approaches is presently unknown, but given identical protein composition of the 2 AR isoforms up to the inclusion of CE3 or exon 4, respectively, it is unlikely that such a wide discrepancy could be exclusively accounted for by the contrasting protein C-termini. Importantly, we identified 422 AR-V7–interacting proteins common between –IR and +IR treatment suggesting that the composition of AR-V7 complexes in cells is reasonably stable and does not markedly change upon DNA damage. That said, a number of transcription-associated proteins were found to be less abundant in response to IR, including SP1, TOP1, SMARCC1, SMARCB1, and FOXA1, which could indicate a subtle shift in AR-V7 transcriptional function during the DDR. Subsequent functional annotation clustering identified *splicing* activities as highly enriched for AR-V7 binding proteins in both –IR and +IR treatment arms. This is consistent with functional classifiers of the ARv567es interactome defining *RNA binding* as a key cellular process (41), with several of the shared AR-V7 and ARv567es binding proteins being involved in RNA interaction and splicing. A key consideration, however, is whether these findings are simply an artifact of detecting proteins involved in the coupled processes of transcription and splicing concomitant with AR-V7–mediated gene expression. Critically, this remains to be clarified, but given the breadth of splicing-associated functions identified within the AR-V7 interactome, such as spliceosome (SF3B3) and subsequent polyadenylation activities (CPSF7), it is intriguing to speculate that AR-Vs have additional roles in RNA metabolism outside of transcription.

Confirming the finding that DNA-PKcs interacted with AR-V7 (38), and providing evidence of a novel interaction with the other DNA-PK holoenzyme components Ku70 and Ku80, helped to validate our APEX2-mediated biotinylation pipeline. Furthermore, these findings supported the notion that DNA-PKcs controls AR-V activity, as has been observed for AR-FL (60), and could form the basis of a tractable opportunity to therapeutically target AR-Vs. To that end, we showed that DNA-PKcs is recruited to *cis*-regulatory elements of AR-V target genes and facilitated their expression. As such, diminished canonical AR-V signaling by either DNA-PKcs knockdown or pharmacological blockade using multiple DNA-PKcs kinase inhibitors was found to compromise AR-V–positive PC cell proliferation implicating an important role for DNA-PKcs in regulating AR-V function. Our global transcriptomics analyses of CWR22Rv1-AR-EK cells subjected to DNA-PKcs depletion or inhibition, which demonstrated a robust negative enrichment of the “hallmark androgen response” gene set, further supports the concept that DNA-PKcs is a key coregulator of AR-V–mediated transcription. Interestingly, we found a greater degree of overlap of our AR-V transcriptome (16) with DEGs from DNA-PKcs knockdown compared with DNA-PKcs blockade suggesting that a kinase-independent function of DNA-PKcs predominates regulation of AR-Vs. Although mechanistic insight into this phenomenon is currently lacking, evidence from numerous other kinases, such as PAK1, indicate a noncatalytic scaffolding function of these proteins plays a key role in signal transmission and pathway regulation (61, 62). Crucially, interrogation of a recently published list of DNA-PKcs–interacting proteins identified a 37% overlap with our AR-V7 interactome (34). This finding indicated a considerable commonality between DNA-PKcs and AR-V7 complexes that may provide evidence that DNA-PKcs assembles a transcription-ready complex recruited to target genes by chromatin-bound AR-Vs. Interestingly, a considerable number of common DNA-PKcs and AR-V interactors were splicing-associated proteins, such as DDX5 and numerous HNRNPs, providing evidence that DNA-PKcs, in conjunction with AR-Vs, may regulate mRNA processing. Additionally, we observed that (a) the “spliceosome” gene set was downregulated in cells depleted of DNA-PKcs or treated with DNA-PKIs NU5455 and NU7441, which is supported by NU7441 treatment of C4-2 cells (Supplemental Figure 31 and ref. 47), and (b) numerous splicing alterations were evident upon loss of DNA-PKcs activity, supporting the concept that DNA-PKcs is a key node for cellular splicing, both de novo and transcriptionally. Ultimately, our data further expand the pleiotropic functionality of the kinase in PC into RNA metabolism beyond simply transcription.

One consequence of these splicing alterations in response to compromised DNA-PKcs activity was the detection of markedly reduced expression of multiple *AR-Vs* in PC cell lines. We subsequently identified RBMX as a suitable candidate for regulating pathogenic splicing in advanced PC. By qRT-PCR and ChIP analyses, we showed that *RBMX* is a direct target gene of DNA-PKcs and detected a significant correlation of *DNA-PKcs* and *RBMX* transcript levels in several large PC patient data sets. Partial rescue of AR-V transcript levels by RBMX overexpression in cells treated with DNA-PK inhibitors supports DNA-PKcs-RBMX interplay in controlling AR-V expression. Importantly, RBMX knockdown reduced transcripts of multiple *AR-Vs*, and *AR-FL*, without affecting *AR* pre-mRNA abundance, suggesting that RBMX controls *AR* mRNA processing downstream of transcription. This notion is supported by the demonstration that (a) RBMX interacts with the phosphorylated C-terminal domain of RNA polymerase II to cotranscriptionally regulate splicing (63); (b) RBMX selectively interacts with *AR* pre-mRNA but not mature *AR-V7*; (c) RBMX depletion does not affect *AR* isoform turnover or ectopic expression of prespliced *AR-V7* cDNA; and (d) *RBMX* and *AR-V7* transcript abundance correlate in PC. Mechanistically, we provide evidence that RBMX enhances inclusion of spliced exon 2–exon 3–containing mRNAs, which encode many clinically relevant *AR-Vs*, including *AR-V1*, *AR*-*V6*, *AR*-*V7*, and *AR*-*V9* and *AR-FL*, explaining the observed reduction in these transcripts when RBMX is depleted. All together, we have uncovered a regulatory pathway involving DNA-PKcs and RBMX that controls generation of *AR-Vs*.

When considering the clinical relevance of these findings we have used two independent CRPC patient transcriptome cohorts to demonstrate that both *PRKDC* and *RBMX* mRNA expression associate with *AR/AR-V7* mRNA expression and AR/AR-V7 activity scores in one or more cohort studied, suggesting a role in *AR* RNA processing. Despite these interesting findings, it is important to consider the limitations of these studies, which include potential discordance between mRNA and protein expression or activity, and heterogenous patient cohorts in terms of both treatments received and timing of the CRPC biopsies. Consistent with this, these interesting findings will need to be further validated using orthogonal methods, in larger patient cohorts with homogenous treatments, preferably in prospective clinical studies with multivariate analyses to confirm both the prognostic and predictive nature of DNA-PK (PRKDC) and RBMX in CRPC.

Our findings provide a rationale for applying DNA-PKIs in AR-V–positive patients with PC by demonstrating they partially inhibit AR-V signaling and decrease AR-V synthesis, which together decrease variant-driven PC cell growth. Of note, however, is that, although each of the tested DNA-PKIs demonstrated comparative phenotypic impact, there was considerable discrepancy in the magnitude of gene expression changes among NU5455, NU7441, and AZD7648, with the former demonstrating considerably more DEGs than the other 2 compounds. NU5455 is more selective than the first-generation DNA-PKI NU7441 (64), which may enable more robust cellular engagement with DNA-PKcs to provide more stable repression of the kinase, which, to that end, could affect a greater repertoire of genes within the tested time frame. Importantly, the transcriptional effect of NU7441 in our study is consistent with what has been observed for the C4-2 cell line (47). Outside of potential variation in drug-protein engagement, the interactome of DNA-PKcs may also be sensitive to binding of distinct DNA-PKIs. Knowing that proteins involved in RNA processing and glycolytic activities are components of the DNA-PKcs complex (34), dynamic flux to such protein-protein interactions in response to DNA-PKI binding may contribute to the observed differences in the DNA-PKI transcriptomes.

In all, our data have provided robust evidence that DNA-PKcs is a key regulator of AR-Vs at multiple levels and suggest a tractable route for AR-V inactivation using clinically relevant DNA-PKIs.

## Methods

### siRNA screen of spliceosome related genes.

The siRNA screen of 315 genes related to the spliceosome in 22Rv1 cells was performed previously (56). AR, AR-V7, and GAPDH protein expression was quantified by Western blot densitometry. For each of the 315 spliceosome-related genes, AR and AR-V7 protein expression was normalized to both GAPDH protein expression and control siRNA.

### Statistics.

Unless otherwise stated, all graphical data represent the mean of 3 individual experiments, and data are shown as the mean ± SEM. For analysis of DNA-PKcs inhibition on AR-mediated gene expression, ChIP, and cell viability in CWR22Rv1-AR-EK cells, 1-way ANOVA was conducted. For cell viability in CWR22Rv1 and VCaP cells, a 2-way ANOVA was conducted using Prism 8 software. *P* values of less than 0.05 were considered significant.

### Study approval.

Approval for patient involvement in this study was granted by the Royal Marsden Hospital Ethics Review committee (reference 04/Q0801/60), as described in Fenor de la Maza et al (50).

### Data availability.

RNA-Seq data generated in this study are publicly available at GEO (accession GSE242255). Additional information can be found in the Supplemental Methods. Values for all data points in graphs are reported in the Supporting Data Values file. See complete unedited blots in the supplemental material.

## Author contributions

BA performed most of the experiments and, together with NB, developed the biotinylation pipeline. LW, RD, SL, JMJV, AP, JW, and LG performed some experiments. SM and RJSB provided guidance on biotinylation sample preparation and conducted all proteomics. BA, NB, SL, DB, WY, and GS performed all bioinformatics. SRM, CNR, EW, RH, PR, IH, AS, JSDB, and LG devised the project. BA, AS, and LG wrote the paper.

## Supplementary Material

Supplemental data

Supplemental tables 1-21

Supporting data values

## Version 1 09/26/2023

In-Press Preview

## Version 2 11/15/2023

Electronic publication
