# Supplementary material for: The catalytic subunit of DNA-PK regulates transcription and splicing of AR in advanced prostate cancer
Source: J Clin Invest. 2023 Nov 15;133(22):e169200. doi: 10.1172/JCI169200 (PMC10645393; doi:10.1172/JCI169200)

Supplementary Information to accompany Adamson *et al*.

Contents

Supplementary Materials and Methods Pages 2 - 6

Supplementary Figure Legends (S1-S31) Pages 7 – 12

Supplementary References Pages 13-14

Supplementary Figures (S1-S31) Pages 15 - 45

Supplementary Tables (S1-S21) see accompanying Excel file

Unedited blots and gels Pages 46 - 56

Supplementary Materials and Methods

Cell culture and compounds

CWR22Rv1 (ATCC CRL-2505) and VCaP (ATCC CRL-2876) cells lines were purchased from ATCC and maintained at 5% CO_2 and at 37°C. The CWR22Rv1-AR-EK cell line is a CRISPR knock-in derivative of CWR22Rv1 that lacks expression of AR-FL, but retains expression of all AR-Vs (1). CWR22Rv1 and CWR22Rv1-AR-EK cells were maintained in RMPI-1640 (R5886, Sigma-Aldrich), and VCaP cells in Dulbecco's Modified Eagle's Medium (R6171, Sigma-Aldrich); each supplemented with 10% (v/v) foetal bovine serum (Sigma-Aldrich) and 2 mmol/L L-glutamine (Sigma-Aldrich). All cell lines were subject to regular mycoplasma testing and STR profiling. For experiments requiring steroid-depleted conditions, cells were cultured in their appropriate media supplemented with 10% (v/v) dextran-coated charcoal-stripped foetal bovine serum (HyClone) and 2 mmol/L L-glutamine. DNA-PKcs inhibitors NU7441 and NU5455 (Newcastle University) and AZD7648 (Selleckchem) were dissolved in DMSO and stored at -20°C. DNA-PKcs inhibitors were applied to cells at a range of concentrations from 0.1-5.0 µM for assay-specific durations in parallel with vehicle controls.

siRNAs and plasmid transfections

siRNAs (see Supplementary Table S1 for sequences) were transfected into cells using Lipofectamine RNAiMAX transfection reagent (Thermo) to a final concentration of 25 nM for either 72 hours for downstream quantitative RT-PCR (qRT-PCR) analysis or 120 hours for cell proliferation assays. In each instance, scrambled siRNA (siScr) was utilised as a transfection control. For DNA transfections, an empty vector control, pCMV-GFP-RBMX (a kind gift from Prof David Elliott, Newcastle University, UK), pLV-FLAG-APEX2-AR-V7 (see below) and an AR-V7 minigene (2) were transiently transfected using TransIT-LT1 transfection reagent according to manufacturer's recommendations (Mirus) for 72 hours prior to RNA immunoprecipitation, western analysis, proximity biotin labelling and quantitative PCR, respectively. For mRNA stability assays, cells depleted of RBMX for 72 hours were subject to 5 µM actinomycin-D treatment for 0-3 hours prior to qRT-PCR and western analyses. RBMX rescue experiments were conducted in CWR22Rv1 cells transfected with control or pCMV-GFP-RBMX plasmids for 48 hours prior to 1 µM NU7441 treatment for 24 hours prior to qRT-PCR analysis.

Quantitative PCR and western blot analysis

Quantitative PCR (qPCR) was used to assess expression of AR-V target genes, DNA-PKcs and those identified from RNA sequencing experiments (see Supplementary Table S2 for primer sequences) using cDNA generated from Trizol-based RNA extractions according to manufacturer's recommendations (Thermo). Briefly, 1 µg of RNA was reverse transcribed using M-MLV reverse transcriptase (Promega) before qPCR analysis, incorporating Platinum SYBR Green qPCR SuperMix (Invitrogen), on a QuantStudio 7 Flex Real-Time PCR machine (Applied Biosystems) using the following thermal profile: 50°C for 2 minutes and 95°C for 10 minutes followed by 40 cycles of 95°C for 15 seconds and 60°C for 1 minute, before melt curve analysis to assess non-specific amplification. The $\Delta\Delta$CT method was used to quantify the targets of interest using QuantStudio Real-Time PCR software (Applied Biosystems), incorporating *RPL13A* as a housekeeping gene. Data are presented as mean normalized gene expression of at least 3 independent experiments +/- SEM.

Western blotting was performed as described in (3) using antibodies, listed in Supplementary Table S3, applied overnight to membranes in 1% non-fat skimmed milk (Marvel) at 4°C. Secondary HRP-conjugated polyclonal rabbit anti-mouse (P0260) and swine anti-rabbit (P0217) antibodies (Dako) were used at 1:4000 for 1 h at room temperature.

Chromatin immunoprecipitation (ChIP) and RNA immunoprecipitation (RIP)

ChIP assays were performed as described in (4) using AR (BD and Cell Signalling Technology), DNA-PKcs (Abcam) and either mouse or rabbit IgG isotype control antibodies (Diagenode) (Table 3). Cells were seeded at a density of 5 x 10^6 in 15 cm dishes and typically cultured to approximately 80% confluency before harvesting. For ChIP experiments involving DNA-PKcs inhibition, 1 μM NU7441 was added 24 hours prior to harvest. 70 μg of sonicated chromatin was added to Protein-A-conjugated Dynabeads (Life-Sciences) pre-incubated with 2 μg of appropriate antibody. DNA was then reverse-crosslinked and purified using DNA mini-prep column-based purification (Sigma) and subject to qPCR analysis using the primers listed in Table 2. Data shown is presented as the mean normalised percentage input of three independent experiments.

For RIP, CWR22Rv1 cells were seeded in steroid-depleted media at a density of 4 x 10^6 and transfected with 10 µg GFP-RBMX-encoding plasmid for 72 hours before a second transfection of 10 µg pGFP-RBMX for a further 48 hours. Cells were then fixed by adding 0.2% formaldehyde to culture media for 15 minutes before quenching with 125 mM glycine and collection in PBS containing protease inhibitors (Roche) and centrifugation at 400g for 5 minutes. Resultant cell pellets were lysed in 200 µL RNase free RIPA buffer (pH 7.5) on ice for 20 minutes before sonication for 3 cycles of 30 seconds on/30 seconds off using a Bioruptor (Diagenode). Pellets were cleared by centrifugation at 15 000 x g for 15 minutes. 10% inputs were then taken and the remaining supernatant was made up to 500 µL in NT2 (50 mM Tris, 150 mM NaCl, 1 mM $MgCl_2$ and 0.05% IGEPAL) plus 200U RNaseOUT™ (Thermo), 400 μM VRC, 1mM DTT, 15mM EDTA and incubated at 4 °C overnight with Dynabeads conjugated to 5 μg antibody of interest or isotype control. Beads were then washed 7 times in NT2 before eluting immune-complexes in 100 µL NT2 containing 10 µL proteinase K and 200 mM NaCl. Input samples were thawed and supplemented with 95 ml NT2, 200 mM NaCl and 2 μl proteinase K, and together with the IP samples incubated at 42°C for 1 hour then 55°C for 1 hour with regular vortexing. RNA was extracted using Trizol and GlycoBlue (Invitrogen), to increase RNA yield, and resuspended in 15 µL nuclease free water. For analysis of pre-mRNA, samples were further treated with DNase. 1 µg RNA was reverse transcribed and resulting cDNA was analysed by qPCR. Target transcript abundance in each IgG/RBMX RIP was normalised to housekeeping gene *RPL13A*. The same calculation was performed for IgG/RBMX input samples and RIP abundance normalised to its respective input. Relative RIP/input enrichment was then compared between RBMX and IgG.

Cell proliferation and cell cycle analysis

For cell count assays, cells were seeded at a density of 10^5 cells per well of a 6 well plate (Corning) for 24 hours prior to drug treatment or seeded on the same day as siRNA transfection and incubated for 120 hours before manual counting using a haemocytometer. For cell cycle analysis, cells were seeded as above and cultured post-drug treatment/-siRNA transfection for 72-96 hours. Cells were then harvested using trypsin, washed in PBS before resuspension in 100 µL citrate buffer (250 mM sucrose, 40 mM sodium citrate, pH7.6) before adding 400 µL of DNA staining buffer (20 µg/mL propidium iodide (PI), 0.5 mM EDTA, 0.5% NP40, 10 µg/µL RNase A) and then incubated at 4 °C for at least 1 hour before analysis using an Attune Flow Cytometer (Beckman) to acquire data from at least 10,000 events. Cell debris and aggregates were removed from the analysis by gating single cell populations.

Histogram plots were generated using FCS Express (DeNovo Software) and % of cells in each cell cycle phase were quantified and compared to the control.

RNA sequencing and data analysis

CWR22Rv1-AR-EK cells were transiently transfected for 72 hours with either siScr control or DNA-PKcs-targeting siRNAs, with additional siScr samples being treated with 1 µM NU7441, NU5455 or AZD7648 for the final 24 hours (see Supplementary Figure S15A). RNA was then extracted using Trizol and 1 µg RNA of each sample was subject to library preparation using TruSeq Stranded mRNA library prep kit (Illumina) (performed by GeneWiz). Resultant libraries were subject to paired end 2x 150 bp sequencing on an Illumina NovaSeq sequencer; generating an average of 20 million reads per sample. Samples were then quality checked using FastQC (v0.11.9) and a genome index generated with STAR (v2.7.0e)(5) genomeGenerate mode using the GENCODE GRCh38 primary assembly and associated GTF annotation (release v36). SAM files were generated against this index using STAR (v2.7.0e) with default settings. SAMtools (v1.11)(6) was subsequently used for sorting files by read name and conversion to BAM format. A gene counts matrix was created with featureCounts (Subread v1.4.0) (7) using GENCODE primary assembly GTF annotation (release v36). All listed steps were performed using the Rocket High Performance Computing service at Newcastle University and the resulting counts matrix was exported for subsequent analyses. Gene-level counts and associated sample information were read into the RStudio IDE, and DESeq2 (v1.28.0) (8) was used for differential expression analysis. log2 fold change shrinkage was applied using the ashr algorithm (9) within DESeq2 and Annotables (v0.1.91) (was used to match Ensembl gene IDs to HGNC symbols and associated information. Significantly differentially-expressed genes were defined as those with fold change +/- 1.5 and adjusted p-value < 0.05. Heatmap visualisation of sample and gene hierarchical clustering was performed with DESeq2 normalised counts and z-score scaled by gene, using pheatmap(v1.0.12) with default settings. Genes were sorted by log2 fold change for gene set enrichment analysis using GSEA software (v4.0.3)(10), KEGG and Hallmark Broad Institute and MSigDB gene sets. The R Tidyverse suite was used for data processing throughout.

For RBMX transcriptomic and splicing analysis, CWR22Rv1 cells grown in steroid-depleted media and depleted of RBMX by siRNAs for 72 hours were harvested for RNA extraction using Trizol before 2x150bp sequencing on the Illumina® NovaSeq™ platform at a read depth of 100M. Differential gene expression analysis was performed as above using STAR-featurecounts and DESeq2 with adapter trimming using Cutadapt prior to alignment (11). Splicing analyses of RNA-seq data from RBMX-depleted cells were performed using SUPPA2 (v.2.3)(12) and DEXSeq (v.1.42.0)(13). For SUPPA2 analysis, trimmed fastq files were quantified using salmon (v.1.6.0)(14) and differential splicing analysis was performed using the Ensembl annotation GRCh38 (hg38) v.109. For DEXSeq analysis, trimmed fastq files were aligned to the Ensembl GRCh38 genome reference using hisat2 (v.2.2.1)(15), and exon coverage was quantified using htseq (v.2.0.3) (16). All output data were processed using tidyverse (v.2.0.0) (17) and biomaRt (v.2.52.0)(18) in R (v.4.2.2). Statistical analyses of junction quantified by hisat2 were performed using Welch's unpaired 2-tailed t-test on Graphpad Prism (v.10.0.0).

Matched tumour vs normal TCGA-PRAD

RNA-seq gene counts, derived from HTSeq, were downloaded from the TCGA-Prostate Adenocarcinoma (PRAD) cohort using R package TCGAbiolinks (v2.15.3) (19). Data was filtered to only include those patients for which matched tumour/adjacent normal tissue was available (n=51). Matched tumour vs normal differential gene expression analysis was conducted using an additive

model accounting for paired samples with edgeR (v3.32.0) (20). edgeR TMM-normalised log2 counts-per-million were extracted for visualisation and plotting (using Prism).

Analysis of datasets available on Gene Expression Omnibus (GEO)

Microarray gene expression data for mCRPC (n = 27) vs localised treatment-naïve prostate cancer (n=49) was accessed through GEO series GSE35988 microarray platform GPL6480 (21). Differential gene expression analysis between disease types was performed using limma (22) via the GEO2R online tool (23). log2 expression values generated by GEO2R were extracted for visualisation and plotting (Prism).

Cell line AR-variant transcriptome data was accessed through GEO series GSE126306 (1). Raw counts from GSE126306 were read into the RStudio IDE, and DESeq2 (v1.28.0)(8) was used for differential expression analysis between AR-V knockdown (siARex1) versus siScr control CWR22Rv1-AR-EK cells. log2 fold change shrinkage was applied using the ashr algorithm within DESeq2. Significantly differentially-expressed genes were defined as those with fold change +/- 1.5 and adjusted p-value < 0.05.

Darolutamide-treated VCaP transcriptome data was accessed through GEO series GSE148397 (24). Raw counts from GSE148397 were read into the RStudio IDE and filtered for VCaP samples treated with 1 nM R1881 for 22 hours +/- 2 µM darolutamide. DESeq2 (v1.28.0) was used for differential expression analysis between 2 µM darolutamide versus DMSO-treated VCaP. log2 fold change shrinkage was applied using the ashr algorithm within DESeq2 and resulting differential expression results were exported for comparison with NU5455 and siDNA-PKcs treated CWR22Rv1-AR-EK. Heatmap visualisation of sample and gene hierarchical clustering was performed with DESeq2 normalised counts, z-score scaled by gene, using pheatmap (v1.0.12) with default settings. All RNA-sequencing data will be available on GSEA (raw data and gene level counts - STAR count matrix) and R scripts made available on GitHub.

CRPC patient transcriptome analyses

CRPC transcriptomes from the Stand Up To Cancer/Prostate Cancer Foundation (SU2C/PCF) cohort were downloaded and re-analysed (25,26). CRPC transcriptomes from the Institute of Cancer Research/Royal Marsden Hospital (ICR/RMH) cohort were reanalysed (27). Paired-end transcriptome sequencing reads for each of the SU2C/PCF (n = 159) and ICR/RMH (n = 95) cohorts were aligned to the human reference genome (GRCh37/hg19) using Tophat2 (v2.0.7). Gene expression, Fragments Per Kilobase of transcript per Million mapped reads (FPKM), was calculated using Cufflinks. The top expressed genes (n = 15000) were analysed for each cohort respectively. The Spearman correlation coefficient (r value) between each gene's expression (FPKM) and other parameters (AR and AR-V7 activity scores) was calculated. AR and AR-V7 activity scores were derived using the previously described 43-gene and 59-gene scores respectively (28,29).

Study approval

Approval for patient involvement in this study was granted by the Royal Marsden Hospital Ethics Review committee (reference no. 04/Q0801/60) as described in Fenor de la Maza *et al*., 2023 (27).

Proximity labelling and mass spectrometry data acquisition and analysis

The pLV-FLAG-APEX2-AR-V7 plasmid was generated by firstly amplifying the APEX2 cDNA from the plasmid pEJS578_DD-dSpyCas9-mCherry-APEX2 (Addgene #108570)(primers in Table 2; forward primer contains a 5'-FLAG tag in-frame with APEX2) and ligated into *Eco* RV-digested pLV-AR-V7 (2). For proximity labelling experiments, CWR22RV1-AR-EK cells were seeded at a cell density of 5 x 10^6 in

15 cm dishes and transfected both on the day of seeding and 48 hours later with 10 μg pLV-FLAG-APEX2-AR-V7 plasmid. After a further 24 hours incubation, cells were subject to -/+ labelling as described in (30); with control representing APEX2-AR-V7-expressing cells not subject to biotin labelling reaction. Briefly, culture media was changed to RPMI media containing 500 μm biotin phenol (Iris Biotech LS-3500) before 4 Gy cell irradiation and incubation for 2 hours at 37°C. Biotin labelling was activated by addition of 1 mM H_2O_2 (Sigma-Aldrich) to culture media for 2 minutes at room temperature with constant agitation. Control experimental arms did not undergo H_2O_2 treatment. Cells were washed 4 times in quenching buffer (100 mM sodium ascorbate, 10 mM TROLOX and 10 mM sodium azide in PBS) and then 4 times in PBS before collection by trypsin and centrifugation at 400 x g for 10 minutes. Resultant cell pellets were resuspended in 1 mL of PBS and centrifuged at 400 x g for 5 minutes before nuclear fractionation, using the NE-PER™ Nuclear and Cytoplasmic Extraction kit (Thermo), and protein quantification. Input samples were taken to determine the labelling efficiency by western blot incorporating an anti-biotin primary antibody (Table 3). 110-150 µg nuclear lysate was then incubated with 30 µg Streptavidin-conjugated magnetic beads (Pierce) overnight at 4°C before 7 sequential washes in 1 mL RIPA buffer and 7 in 1 ml PBS before resuspension in 1 ml PBS and stored at – 80°C until analysis by mass spectrometry (Glasgow Polyomics, University of Glasgow).

Protein identification was assigned using the Mascot search engine (v2.6.2, Matrix Science) to interrogate protein sequences in the Swissprot database using Homo sapiens taxonomy. A mass tolerance of 10 ppm was allowed for the precursor and 0.3 Da for MS/MS matching. Protein identifications were also assigned using MaxQuant. Thermo RAW files were analysed using MaxQuant (v2.0.3.0)(31) against a UniProt human proteome database FASTA (downloaded January 14th, 2022). Default settings for peptide and protein identification with an FDR of 0.01 were used throughout and Instrument type - Orbitrap was selected. A match-between-runs algorithm was utilised, and calculation of intensity Based Absolute Quantification (iBAQ) values was enabled. The resulting proteinGroups file was exported to the RStudio IDE for processing and data analysis. Potential contaminants, reverse proteins, and proteins only identified by sites were removed and only proteins identified with two or more unique peptides in two out of three biological replicates were considered for further analysis. Corrected iBAQ values were calculated by subtracting the control (unlabelled) iBAQ values from each experimental arm, on a replicate-by-replicate basis. Relative iBAQ (riBAQ) values were calculated by dividing the corrected iBAQ value for that sample/gene by the sum of the iBAQ values for that sample and then log10 transformed. Average riBAQs were calculated of the three (or two if the protein was identified in two out of three replicates) riBAQ values for the corresponding protein and the mean difference was calculated between the minus and plus irradiation experimental arms. Lists in supplementary files include lists of proteins that were found in 2 or more replicates for both minus and plus IR, sorted by mean difference in riBAQ values as well as list of all proteins that were found in 2 or more replicates in each independent treatment arms. Common streptavidin contaminants ACACA and PC were omitted from final protein lists. Cellular component analysis was done using STRING (Szklarczyk et al. Nucleic acids research 47.D1 (2018): D607-D613.2).

Supplementary Figure Legends

Supplementary Figure S1. APEX2-AR-V7 is recruited to AR-V target genes. CWR22Rv1-AR-EK **(A)** and CWR22Rv1 **(B)** were transiently transfected for 48 hours with a FLAG-tagged APEX2-AR-V7 construct prior to chromatin immunoprecipitation (ChIP) using anti-FLAG or isotype control (IgG) antibodies. Enrichment of FLAG-APEX2-AR-V7 at *cis*-regulatory elements of canonical AR-V target genes *PSA/KLK3*, *KLK2*, *CCNA2* and *UBE2C* was assessed by qRT-PCR. Data is representative of a single experiment.

Supplementary Figure S2. Outline of AR-V7 proximal protein biotinylation pipeline. A. Diagrammatic representation of proximal protein biotinylation in cells. CWR22Rv1-AR-EK cells ectopically-expressing APEX2-AR-V7 were treated with biotin-phenol for 2 hours post +/- IR prior to hydrogen peroxide (H_2O_2) treatment for 2 minutes to activate APEX2-mediated proximal protein biotinylation (indicated by red circles attached to AR-V7-interacting proteins). **B.** Flow diagram illustrating the experimental pipeline for detecting the AR-V7 interactome. After completion of the biotin labelling reaction in cells treated with and without ionising-radiation (IR), nuclear isolates were subject to streptavidin-based immunoprecipitation (pull-down) prior to mass spectrometry to identify biotinylated proteins. **C.** 5 x 10^6 HEK293T cells were transfected with 5 µg pLV-FLAG-APEX2-AR-V7 for 72 hours prior to treatment with biotin-phenol and +/- IR (4 Gy) for either 1 or 2 hours. In the minus and plus IR arms, H_2O_2 was added to cells to induce the labelling reaction. Cells were then quenched, harvested and the cytoplasmic and nuclear fractions were isolated and quantified. 10 µg of resultant nuclear lysate was analysed by western blotting using an HRP-linked anti-biotin antibody. **D.** CWR22Rv1-AR-EK cells were transfected with 10 µg pLV-FLAG-APEX2-AR-V7 on the day of seeding and again 48 hours later prior to treatment with biotin-phenol and +/- IR (4 Gy) for 2 hours. In the minus and plus IR arms, H_2O_2 was added to cells to induce the labelling reaction. Cells were then quenched, harvested and the cytoplasmic and nuclear fractions were isolated and quantified. 10 µg of resultant cytoplasmic lysate was analysed by western blotting using an HRP-linked anti-streptavidin antibody. **E.** CWR22Rv1-AR-EK and CWR22Rv1 cells were seeded onto glass coverslips for 24 hours. Cells were then irradiated with 2 Gy ionising radiation and harvested for immunofluorescence analysis of γH2AX foci immediately after irradiation (0h) then 1, 2, 4, 8 and 24 hours post irradiation. γH2AX foci quantification was performed using ImageJ software. Data represents three independent experiments and the mean ±SEM is presented.

Supplementary Figure S3. Analysis of raw proteomics data from individual replicate samples. A. Table showing numbers of proteins identified in each experimental arm (control (-H_2O_2); -IR/+IR (+H_2O_2)) across the three independent experimental repeats. **B.** Heatmap of identified proteins in each experimental arm across three replicates. Control samples represent those lacking H_2O_2 treatment; while +IR and -IR represent samples treated with H_2O_2 in the presence and absence of 4 Gy ionising radiation, respectively. **C.** Principle component analysis of the three experimental arms across the three independent experiments (N=1-3). **D.** Venn diagram indicating overlap between filtered AR-V7 interacting proteins between the minus- and plus-IR experimental arms.

Supplementary Figure S4. Examining AR-V7 interactomes from several studies identified common protein interactors. A. APEX2-AR-V7 interacting proteins were compared to the AR-FL Bio-ID interactome that was generated in LNCaP cells (Velot *et al.*, 2021). **B.** APEX2-AR-V7 interacting proteins were compared to the AR-V^{576es} RIME interactome that was generated using R1-D567 cells (Paltoglou *et al.*, 2017). Final lists of AR-V^{567es} interacting proteins were generated by only including proteins that were identified in 3 out of 3 replicates and only if they were not present in 2 or more IgG control samples. The list used to compare with the APEX2-AR-V7 list was the combined proteins identified in either minus or plus DHT experimental arms. **C.** The APEX2-AR-V7 interactome was compared to the DNA-PKcs RIME interactome that was generated using C4-2 PCa cells (Dylgjeri *et al.*, 2022).

Supplementary Figure S5. DNA-PKcs inhibitors reduce CWR22Rv1-AR-EK and CWR22Rv1 proliferation. A. CWR22Rv1-AR-EK and CWR22Rv1 confluency over 7 days treatment with NU7441 was assessed using Incucyte live cell imaging. Figures are representative of 3 repeats +/- SEM and each data point from each inter-experimental repeat are an average of three intra-experimental repeats. One-way ANOVA using Bonferroni Post Hoc analysis was used to determine the statistical significance for CWR22Rv1-AR-EK and 2-way ANOVA was used for CWR22Rv1 cells (*=p<0.05, **=p<0.01, ***=p<0.001). **B.** CWR22Rv1-AR-EK cells were grown in full media with increasing concentrations of AZD7648 for 5 days before a manual cell count. Cell counts were performed in quadruplicate. Data is representative of three independent repeats ± SEM. One-way ANOVA using Bonferroni Post Hoc analysis was used to determine the statistical significance (* = p < 0.05, ** = p < 0.01, **** = p < 0.0001). **C.** Calculated GI_{50} values for each of the three DNA-PKcs inhibitors calculated from data shown in Figure 2.

Supplementary Figure S6. DNA-PKcs blockade using NU7441 and NU5455 selectively impacts cell cycle. CWR22Rv1-AR-EK cells were cultured in serum-containing media containing either DMSO, 1 or 5 µM NU7441 or NU5455 for 48 hours. Cell cycle analysis was performed using PI flow cytometry. Data represents mean number of cells in each cell cycle phase ± SEM. Two-way ANOVA with Tukeys correction was used to determine statistical significance (** = p < 0.01).

Supplementary Figure S7. DNA-PKcs inhibition reduces AR-V-driven gene expression in CWR22Rv1-AR-EK cells. A.. CWR22Rv1-AR-EK cells cultured in serum-containing media were treated with increasing concentrations of NU7441 for 24 hours before AR-target gene expression analysis using qRT-PCR. **B.** CWR22Rv1-AR-EK cells were cultured in serum-containing media and treated with 1 µM AZD7648 for 24 hours before AR-target gene expression analysis using qRT-PCR. Data was normalised to the DMSO treatment arm for each target gene. Data is representative of three independent repeats ± SEM. One-way ANOVA using Bonferroni Post Hoc analysis was used to determine the statistical significance (* = p < 0.05, ** = p < 0.01, **** = p < 0.0001).

Supplementary Figure S8. DNA-PKcs inhibition with AZD7648 selectively diminishes AR-V-target gene expression. A. CWR22Rv1-AR-EK cells cultured in serum-containing media were treated with increasing concentrations of NU7441 for 24 hours before AR-target gene expression analysis using qRT-PCR. VCaP cells grown in steroid-depleted media were treated with either a dose range of NU7441 **(B)** or 1 µM NU5455 **(C)** for 24 hours prior to AR-target gene expression analysis using qRT-PCR. Data was normalised to the DMSO treatment arm for each target gene. Data is representative of three independent repeats ± SEM. One-way ANOVA using Bonferroni Post Hoc analysis was used to determine the statistical significance (* = p < 0.05, ** = p < 0.01)

Supplementary Figure S9. DNA-PKcs depletion using a commercially-available siRNA pool shows effects on AR-V activity largely inconsistent with DNA-PKcs blockade. CWR22Rv1-AR-EK **(A)** and CWR22Rv1 **(B)** cells were transfected with either a DNA-PKcs siRNA pool or scrambled (siScr) control before harvesting at 48 and 72 hours for respective qRT-PCR analysis of AR-target genes and western blot analysis of DNA-PKcs, AR and a-tubulin levels. qRT-PCR data is normalised to the siScr arm. Data is representative of three independent repeats +/- SEM. One-way ANOVA using Bonferroni Post Hoc analysis was used to determine the statistical significance (* = p <0.05, ** = p <0.01, *** = p<0.001).

Supplementary Figure S10. DNA-PKcs depletion using a commercially-available siRNA pool has no effect on CWR22Rv1-AR-EK and CWR22Rv1 cell growth. CWR22Rv1-AR-EK and CWR22Rv1 cells grown in respective serum-containing and steroid-depleted media were transfected with either a DNA-PKcs-targeting siRNA pool (siDNA-PKcs-SP) or scrambled control (siScr) siRNA for 24 hours before Incucyte-based live-cell imaging over 7 days to assess confluency. Data is representative of three independent experiments.

Supplementary Figure S11. Deconvoluted DNA-PKcs-targeting siRNA pool show differential effects on PC cell growth. CWR22Rv1-AR-EK and CWR22Rv1 cells grown in respective serum-containing and steroid-depleted media were transfected with the four individual oligonucleotides from the DNA-PKcs siRNA pool (siDNA-PK1-4) or a scrambled control (siScr) siRNA for 96 hours prior to cell counts. Data is representative of 3 repeats and data is normalised to the siScr control and represents mean ± SEM (n=3). One-way ANOVA using Bonferroni Post Hoc analysis was used to determine the statistical significance (* = $p < 0.05$, ** = $p < 0.01$, *** = $p < 0.001$, **** = $p < 0.0001$).

Supplementary Figure S12. Deconvolution of the DNA-PKcs-targeting siRNA pool shows differential effects on AR-V transcriptional activity. CWR22Rv1-AR-EK **(A)** and CWR22Rv1 **(B)** cells grown in respective serum-containing and steroid-depleted media were reverse transfected with the four individual oligonucleotides from the DNA-PKcs siRNA pool (siDNA-PK1-4) or a scrambled control (siScr) siRNA for 72 hours. Cells were then subject to AR-target gene expression analysis using qRT-PCR. Data was normalised to the siScr treatment arm for each target gene and is representative of three independent repeats +/- SEM. One-way ANOVA using Bonferroni Post-Hoc analysis was used to determine the statistical significance (* = $p < 0.05$, ** = $p < 0.01$).

Supplementary Figure S13. DNA-PKcs knockdown reduces AR target gene expression. Expression of canonical AR target genes in CWR22Rv1 cells is diminished upon 72 hour DNA-PKcs knockdown using siDNA-PKcs 2-4 pool. Data represents the mean normalised expression of three independent experiments +/- SEM (***=$p<0.001$; ****=$p<0.0001$ as determined using an unpaired 2-tailed t-test).

Supplementary Figure S14. Cell cycle analysis reveals DNA-PKcs manipulation in CWR22Rv1-AR-EK cells increase G1 at the expense of S phase, but does not impact steady-state DNA damage. A. CWR22Rv1-AR-EK cells grown in serum-containing media were transfected with siScr or siDNA-PK for 72 hours prior to cell cycle analysis using propidium iodide-based flow cytometry. Data represents mean number of cells in each cell cycle phase ± SEM. Two-way ANOVA with Sidak's correction was used to determine statistical significance (**=$p<0.01$). **B.** CWR22Rv1-AR-EK cells pre-treated with either DMSO or 1 µM NU7441 for 1 hour were subject to -/+ 2 Gy ionising radiation (IR) treatment and **γ**H2AX levels monitored up to 96 hours post IR. Representative **γ**H2AX immunofluorescence is shown in the upper panel and foci quantification using ImageJ is graphically represented. Lower left panel represents positive control IR-treated cells while right-hand panel represents **γ**H2AX foci in response to 1 µM NU7441 treatment for the duration of the experiment. Data is the average of three independent experiments +/- SEM (**=$p<0.01$ as determined using an unpaired 2-tailed t-test).

Supplementary Figure S15. AR recruitment to *cis*-regulatory elements of canonical AR-target genes is refractory to DNA-PKcs inhibition. VCaP cells were seeded in steroid-depleted media and transfected with DNA-PKcs (siDNA-PKcs) and scrambled (siScr) siRNA for 72 hours prior to ChIP analysis. AR enrichment at *PSA*, *KLK2* and *UBE2C* cis-regulatory elements over an IgG isotype control was determined by qPCR. Data is displayed as fold enrichment normalised to the DMSO control. Data is representative of three independent repeats +/- SEM. One-way ANOVA using Bonferroni Post Hoc analysis was used to determine the statistical significance (*=$p<0.05$, **=$p<0.01$, ***=$p<0.001$). **B.** CWR22Rv1 cells were depleted of topoisomerase 1 (TOPO1) by siRNA (siTOPO1) for 48 hours prior to quantitative RT-PCR to assess TOPO1 mRNA levels normalised to scrambled siRNA (siScr) control**.** Data represents the mean of three independent repeats +/- SD. **C.** Cells depleted of TOPO1 as in (B) were subject to chromatin immunoprecipitation using either anti-DNA-PKcs or isotype control (IgG) antibodies prior to quantitative PCR to assess normalised fold enrichment of DNA-PKcs in response to TOPO1 knockdown compared to scrambled (siScr) control. Data represents the mean of three independent experiments +/- SEM (* $p<0.05$; ** $p<0.01$).

Supplementary Figure S16. Quality control of RNA-sequencing samples. A. RNA sequencing experimental set-up to assess global transcriptomic effect of DNA-PKcs inhibition with 1 **µ**M NU7441, NU5455 and AZD7648 and DNA-PKcs knockdown in CWR22Rv1-AR-EK cells. **B.** CWR22Rv1-AR-EK cells were transfected with either scrambled control (siScr) or DNA-PKcs-targeting (siDNA-PK) siRNAs and

incubated for 48 hours prior to 24 hour +/- 1 mM DNA-PK inhibitor treatment as outlined in (A) prior to qRT-PCR analysis of DNA-PKcs transcript levels. Graphs show an average of three repeats +/- SEM (* = p < 0.05, ** = p < 0.01, *** = p < 0.001, **** = p < 0.0001). **C.** RNA-sequencing data was input into R studio after genome alignment and quantification. The gene count matrix was input to a principal component analysis to reveal clustering of each experimental arm. A key indicating the different experimental arms is provided to the right of the plot.

Supplementary Figure S17. DNA-PKcs inhibitors have broadly distinct effects on global gene expression. A. Table of differentially-expressed (DE) and significant DE genes in response to DNA-PKcs inhibition and knockdown in CWR22Rv1-AR-EK cells. **B.** MA plots showing the number of up- and down-regulated genes in response to DNA-PKcs inhibition with NU7441 and AZD7648. Events in blue represent statistically-significant differentially-expressed genes (DEGs) (>1.5-fold change; p adjusted < 0.05). **C.** Comparison of overlapping significantly DE genes in response to DNA-PKcs inhibition shows TWIST1 and PDK4 are commonly down- and up-regulated, across each of the three data-sets, respectively. **D.** Number of significantly differentially- expressed KEGG pathways in response to DNA-PKcs inhibition with 1 µM NU5455, NU7441 and AZD7648 and depletion by siDNA-PKcs.

Supplementary Figure S18. Comparing DEGs between DNA-PKI-treated PC cells and between different DNA-PKIs shows limited overlap. A. Significantly differentially-expressed genes (DEGs) in response to NU7441 treatment in CWR22v1-AR-EK and C4-2 cells (Fold change > 1.5 and p value < 0.05). GSE codes Raw counts) were downloaded from GEO and analysed for differential gene expression using DESeq2 for RNA-Seq (Dylgjeri *et al*., 2019) and Limma for microarray (Goodwin et al., 2015) data. **B.** Significantly differentially-expressed genes (DEGs) in response to AR-V knockdown in CWR22Rv1-AR-EK cells (Fold change > 1.5 and p value < 0.05) compared to NU7441-, NU5455- and AZD7648-treated CWR22Rv1-AR-EK cells. DEGs were determined using DESeq2 and the significantly DEGs (p < 0.05, FC > ±1.5) were compared using molecularbiologytools.com.

Supplementary Figure S19. Comparisons between commonly downregulated and upregulated genes between siAR-V and siDNA-PKcs show a considerable number of genes shared between the two transcriptomes. Differentially expressed genes (DEGs) were determined using DESeq2 and the significantly DEGs (p < 0.05, FC > ±1.5) were separated into up-and downregulated lists and compared using Venny 2.0.

Supplementary Figure S20. *PRKDC* (DNA-PKcs) expression correlates with AR-FL and AR-V7 expression and activity in CRPC patient transcriptomes. A. Diagrammatic representation of patient numbers across the Stand Up 2 Cancer/Prostate Cancer Foundation (SU2C/PCF) and Institute of Cancer Research/Royal Marsden Hospital cohorts included in the DNA-PKcs (*PRKDC*) and RBMX expression profiling. Association of *PRKDC* mRNA levels with **(A)** *AR* and *AR-V7* mRNA levels; and **(B)** AR and AR-V7 activity scores, in the ICR/RMH (n=95) CRPC transcriptomes. r values and p values are shown and were calculated using Spearman's correlation.

Supplementary Figure S21. DNA-PKcs depletion and inhibition causes a significant downregulation of spliceosome-associated genes in CWR22Rv1-AR-EK cells. **A.** Unfiltered DEG lists from siDNA-PKcs treatment in CWR22Rv1-AR-EK cells were compared to Hallmark gene lists using GSEA; negatively-enriched pathways are plotted as a bar-chart. The spliceosome pathway is highlighted in green and representative GSEA plot is shown in the lower panel. **B.** Analysis of NU5455-treated CWR22Rv1 expression data as performed in (A).

Supplementary Figure S22. RBMX is identified as a DNA-PKcs-regulated gene up-regulated in prostate cancer. A. RNA-sequencing data derived from CWR22Rv1-AR-EK cells depleted of DNA-PKcs was analysed for differential splicing activity using SUPPA2. A. Events that passed a ΔPSI ± 0.2 were plotted in the pie-chart. **B.** Volcano plot of differential splicing events with events that passed the cut offs of false discovery rate < 0.05 and ΔPSI of 0.6 annotated with their gene ID. **C.** The 34 spliceosome genes that are downregulated in response to DNA-PKcs inhibition and depletion and upregulated in

response to darolutamide are presented in a volcano plot with RBMX highlighted. **D.** Expression of *PRPF4* and *LSM5* was examined in CWR22Rv1-AR-EK cells subject to DNA-PKcs blockade (upper panel) and depletion (lower panel) for 24 and 48 hours, respectively using QRT-PCR. **E.** The genes that are upregulated by 10% in matched tumour vs normal samples from the list of 34 genes are presented; *RBMX* is highlighted in green. *RBMX* expression was plotted against matched normal vs tumour samples **(F)** or Gleason score **(G)** using values from ULACAN (* = $p < 0.05$, ** = $p < 0.01$).

Supplementary Figure S23. Expression of *RBMX* and DNA-PKcs-encoding gene *PRKDC* correlate in prostate cancer. cBioPortal was utilised to interrogate correlation in expression of *RBMX* and the DNA-PKcs encoding gene *PRKDC* in two independent datasets: MSKCC (Taylor *et al.*, 2010) and TCGA (*Cell* 2015).

Supplementary Figure S24. RBMX depletion down-regulates AR-V levels and target gene expression. VCaP cells grown in steroid-depleted media were transfected with either RBMX (siRBMX) or scrambled control (siScr) for 72 hours prior to qRT-PCR analysis of *AR-V1*, *-V6*, *-V9* and *FL-AR* transcripts. Data is representative of three independent repeats ± SEM. An unpaired 2-tailed t-test was used to determine the statistical significance (* = $p<0.05$, **=$p<0.01$). **B.** All 315 genes in the spliceosome related gene set were individually depleted by siRNA in CWR22Rv1 cells. AR, AR-V7 and GAPDH protein levels were determined by western blot densitometry. AR and AR-V7 protein levels (normalised to GAPDH protein levels) for each of the 315 genes investigated normalised to control siRNA (set as 1.0) are shown. RBMX is highlighted (red dot). Dotted lines demonstrate a reduction in AR and AR-V7 expression of > 50% compared to control siRNA.

Supplementary Figure S25. RBMX is an important splicing regulator downstream of DNA-PKI that does not impact AR isoform mRNA turnover. A. CWR22Rv1 cells were subject to transient transfection with either control or RBMX expression vectors for 24 hours prior to DNA-PKcs blockade with 1 µM NU7441 for a further 24 hours prior to either *AR-V1*, *-V7* and *-V9* expression analysis by qRT-PCR or immunoblotting incorporating RBMX and α-tubulin antibodies. Data is the average of four independent experiments +/- SEM (*=$p<0.05$; **=$p<0.01$; as calculated using one-way ANOVA with Dunnet's multiple comparisons test). CWR22Rv1 cells depleted of RBMX (siRBMX) or control (siScr) for 72 hours were subject to 5 µM actinomycin-D treatment for 0-3 hours prior to qRT-PCR analysis of FL-AR and AR-V7. **B.** Quantitative RT-PCR of *FL-AR* and *AR-V7,* and western blotting using anti-AR, -RBMX and -α-tubulin antibodies in cells immediately prior to addition of actinomycin-d (T=0). **C.** Quantitative RT-PCR of *FL-AR* and *AR-V7* in which steady-state transcript levels at T=0 was normalised to 1 in both experimental arms. Data is the average of three independent experiments +/- SEM (***=$p<0.001$).

Supplementary Figure S26. RNA Immunoprecipitation shows RBMX enrichment at FL-AR mRNA transcripts. A. CWR22Rv1-AR-EK cells were transfected with 5 µg GFP-RBMX plasmid for 48 hours prior to immunoprecipitation (IP) using either anti-GFP or isotype control (IgG) antibodies. Immunoprecipitates and input samples were subject to western analysis using GFP and **α**-tubulin antibodies. RNA immunoprecipitation (RIP) using either anti-GFP or isotype control (IgG) antibodies was then conducted. Resultant RNA was reverse transcribed and subject to qRT-PCR analysis using primers specific for *AR* pre-mRNA and mature *AR-V7* transcripts. Data is representative of three independent repeats +/-SEM using an unpaired 2-tailed t-test to determine statistical significance (**=$p<0.01$).

Supplementary Figure S27. Ectopic AR-V7 expression is not impacted by RBMX knockdown or DNA-PK inhibition. A. CWR22Rv1-AR-EK cells transiently transfected with control or APEX2-AR-V7-expressing plasmids were depleted of RBMX, using either individual RBMX-targeting siRNAs or a commercially available siRNA smartpool, for 48 hours prior to western analysis using anti-AR, -RBMX and -α-tubulin antibodies. Data represents two independent experiments. **B.** CWR22Rv1-AR-EK cells were transiently transfected with APEX2-AR-V7 expression plasmids for 24 hours prior to 24-hour

treatment with 1 µM DNA-PKcs inhibitor NU7441 or DMSO control and western analysis using anti-AR and –α-tubulin antibodies. Data represents two independent experiments.

Supplementary Figure S28. RBMX transcriptome implicates a role in AR regulation and shows considerable overlap with DNA-PKcs and AR-V regulated genes. A. RBMX knockdown RNA-sequencing data was input into R studio after genome alignment and quantification. The gene count matrix was input to a principal component analysis to reveal clustering of each experimental arm. A key indicating the different experimental arms is provided to the right of the plot. **B.** Heatmap of DEGs in each experimental arm across the three replicates for each scrambled control (siScr) and RBMX knockdown (siRBMX). **C.** Volcano plot of differentially expressed genes with statistical significance and fold change (Log_2) colour coded and RBMX transcript highlighted. **D.** Venn diagram indicating overlap between RBMX and DNA-PKcs transcriptomes. **E.** Unfiltered DEG lists from siRBMX transfected CWR22Rv1 cells were compared to Hallmark gene lists using GSEA; negatively enriched pathways are plotted as a bar-chart. The spliceosome pathway is highlighted in green. **F.** Representative GSEA plot of the Hallmark Androgen Response. **G.** Venn diagram indicating overlap between RBMX, DNA-PKcs and AR-V (Kounatidou et al., 2019) transcriptomes.

Supplementary Figure S29. RBMX depletion significantly impacts global splicing in CWR22Rv1 cells, but only modestly affects exon 2 – CE4-spliced AR transcripts. A. Diagrammatic representation and quantification of the statistically significant splicing alterations detected in response to RBMX depletion as determined using SUPPA. **B.** Quantitative RT-PCR analysis of exon 2–CE4-spliced transcripts in CWR22Rv1 cells depleted of RBMX (siRBMX) versus control). Data represents the normalised mean expression from three independent experiments +/- SEM (**=p<0.01).

Supplementary Figure S30. RBMX-AR isoform expression and activity correlations in CRPC. A. Correlation of RBMX mRNA levels with *FL-AR* and *AR-V7* mRNA levels in the ICR/RMH (n=95) CRPC transcriptomes. r values and p values are shown and were calculated using Spearman's correlation. **B.** Correlation of RBMX mRNA levels with FL-AR and AR-V7 activity scores in the ICR/RMH (n=95) CRPC transcriptomes. r values and p values are shown and were calculated using Spearman's correlation.

Supplementary Figure S31. Unfiltered DEG lists from NU7441 treatment of C4-2 cells (Dyljgeri *et al.*, 2019) were compared to Hallmark gene lists using GSEA; negative enrichment of the *Spliceosome* hallmark is shown in the GSEA plot.

Supplementary References

1. Kounatidou, E., Nakjang, S., McCracken, S.R.C., Dehm, S.M., Robson, C.N., Jones, D. and Gaughan, L. (2019) A novel CRISPR-engineered prostate cancer cell line defines the AR-V transcriptome and identifies PARP inhibitor sensitivities. *Nucleic Acids Res*, **47**, 5634-5647.
2. Jones, D., Noble, M., Wedge, S.R., Robson, C.N. and Gaughan, L. (2017) Aurora A regulates expression of AR-V7 in models of castrate resistant prostate cancer. *Sci Rep*, **7**, 40957.
3. O'Neill, D., Jones, D., Wade, M., Grey, J., Nakjang, S., Guo, W., Cork, D., Davies, B.R., Wedge, S.R., Robson, C.N. *et al.* (2015) Development and exploitation of a novel mutant androgen receptor modelling strategy to identify new targets for advanced prostate cancer therapy. *Oncotarget*, **6**, 26029-26040.
4. Chaytor, L., Simcock, M., Nakjang, S., Heath, R., Walker, L., Robson, C., Jones, D. and Gaughan, L. (2019) The Pioneering Role of GATA2 in Androgen Receptor Variant Regulation Is Controlled by Bromodomain and Extraterminal Proteins in Castrate-Resistant Prostate Cancer. *Mol Cancer Res*, **17**, 1264-1278.
5. Dobin, A., Davis, C.A., Schlesinger, F., Drenkow, J., Zaleski, C., Jha, S., Batut, P., Chaisson, M. and Gingeras, T.R. (2013) STAR: ultrafast universal RNA-seq aligner. *Bioinformatics*, **29**, 15-21.
6. Li, H., Handsaker, B., Wysoker, A., Fennell, T., Ruan, J., Homer, N., Marth, G., Abecasis, G., Durbin, R. and Genome Project Data Processing, S. (2009) The Sequence Alignment/Map format and SAMtools. *Bioinformatics*, **25**, 2078-2079.
7. Liao, Y., Smyth, G.K. and Shi, W. (2013) The Subread aligner: fast, accurate and scalable read mapping by seed-and-vote. *Nucleic Acids Res*, **41**, e108.
8. Love, M.I., Huber, W. and Anders, S. (2014) Moderated estimation of fold change and dispersion for RNA-seq data with DESeq2. *Genome Biol*, **15**, 550.
9. Gerard, D. and Stephens, M. (2020) Empirical Bayes shrinkage and false discovery rate estimation, allowing for unwanted variation. *Biostatistics*, **21**, 15-32.
10. Subramanian, A., Tamayo, P., Mootha, V.K., Mukherjee, S., Ebert, B.L., Gillette, M.A., Paulovich, A., Pomeroy, S.L., Golub, T.R., Lander, E.S. *et al.* (2005) Gene set enrichment analysis: a knowledge-based approach for interpreting genome-wide expression profiles. *Proc Natl Acad Sci U S A*, **102**, 15545-15550.
11. Martin, M. (2011) Cutadapt removes adapter sequences from high-throughput sequencing reads. *2011*, **17**, 3.
12. Trincado, J.L., Entizne, J.C., Hysenaj, G., Singh, B., Skalic, M., Elliott, D.J. and Eyras, E. (2018) SUPPA2: fast, accurate, and uncertainty-aware differential splicing analysis across multiple conditions. *Genome Biol*, **19**, 40.
13. Anders, S., Reyes, A. and Huber, W. (2012) Detecting differential usage of exons from RNA-seq data. *Genome Res*, **22**, 2008-2017.
14. Patro, R., Duggal, G., Love, M.I., Irizarry, R.A. and Kingsford, C. (2017) Salmon provides fast and bias-aware quantification of transcript expression. *Nat Methods*, **14**, 417-419.
15. Kim, D., Langmead, B. and Salzberg, S.L. (2015) HISAT: a fast spliced aligner with low memory requirements. *Nat Methods*, **12**, 357-360.
16. Anders, S., Pyl, P.T. and Huber, W. (2015) HTSeq--a Python framework to work with high-throughput sequencing data. *Bioinformatics*, **31**, 166-169.
17. Hadley Wickham, M.A., Jennifer Bryan, Winston Chang, Lucy D'Agostino McGowan, Romain François, Garrett Grolemund, Alex Hayes, Lionel Henry, Jim Hester, Max Kuhn, Thomas Lin Pedersen, Evan Miller, Stephan Milton Bache, Kirill Müller, Jeroen Ooms, David Robinson, Dana Paige Seidel, Vitalie Spinu, Kohske Takahashi, Davis Vaughan, Claus Wilke, Kara Woo, and Hiroaki Yutani. (2019) Welcome to the Tidyverse. *Journal of Open Source Software*, **4(43)**, 1686, .

18. Durinck, S., Moreau, Y., Kasprzyk, A., Davis, S., De Moor, B., Brazma, A. and Huber, W. (2005) BioMart and Bioconductor: a powerful link between biological databases and microarray data analysis. *Bioinformatics*, **21**, 3439-3440.
19. Colaprico, A., Silva, T.C., Olsen, C., Garofano, L., Cava, C., Garolini, D., Sabedot, T.S., Malta, T.M., Pagnotta, S.M., Castiglioni, I. *et al.* (2016) TCGAbiolinks: an R/Bioconductor package for integrative analysis of TCGA data. *Nucleic Acids Res*, **44**, e71.
20. Robinson, M.D., McCarthy, D.J. and Smyth, G.K. (2010) edgeR: a Bioconductor package for differential expression analysis of digital gene expression data. *Bioinformatics*, **26**, 139-140.
21. Grasso, C.S., Wu, Y.M., Robinson, D.R., Cao, X., Dhanasekaran, S.M., Khan, A.P., Quist, M.J., Jing, X., Lonigro, R.J., Brenner, J.C. *et al.* (2012) The mutational landscape of lethal castration-resistant prostate cancer. *Nature*, **487**, 239-243.
22. Ritchie, M.E., Phipson, B., Wu, D., Hu, Y., Law, C.W., Shi, W. and Smyth, G.K. (2015) limma powers differential expression analyses for RNA-sequencing and microarray studies. *Nucleic Acids Res*, **43**, e47.
23. Barrett, T., Wilhite, S.E., Ledoux, P., Evangelista, C., Kim, I.F., Tomashevsky, M., Marshall, K.A., Phillippy, K.H., Sherman, P.M., Holko, M. *et al.* (2013) NCBI GEO: archive for functional genomics data sets--update. *Nucleic Acids Res*, **41**, D991-995.
24. Baumgart, S.J., Nevedomskaya, E., Lesche, R., Newman, R., Mumberg, D. and Haendler, B. (2020) Darolutamide antagonizes androgen signaling by blocking enhancer and super-enhancer activation. *Mol Oncol*, **14**, 2022-2039.
25. Robinson, D., Van Allen, E.M., Wu, Y.M., Schultz, N., Lonigro, R.J., Mosquera, J.M., Montgomery, B., Taplin, M.E., Pritchard, C.C., Attard, G. *et al.* (2015) Integrative Clinical Genomics of Advanced Prostate Cancer. *Cell*, **162**, 454.
26. Abida, W., Cyrta, J., Heller, G., Prandi, D., Armenia, J., Coleman, I., Cieslik, M., Benelli, M., Robinson, D., Van Allen, E.M. *et al.* (2019) Genomic correlates of clinical outcome in advanced prostate cancer. *Proc Natl Acad Sci U S A*, **116**, 11428-11436.
27. Fenor de la Maza, M.D., Chandran, K., Rekowski, J., Shui, I.M., Gurel, B., Cross, E., Carreira, S., Yuan, W., Westaby, D., Miranda, S. *et al.* (2022) Immune Biomarkers in Metastatic Castration-resistant Prostate Cancer. *Eur Urol Oncol*, **5**, 659-667.
28. Welti, J., Sharp, A., Yuan, W., Dolling, D., Nava Rodrigues, D., Figueiredo, I., Gil, V., Neeb, A., Clarke, M., Seed, G. *et al.* (2018) Targeting Bromodomain and Extra-Terminal (BET) Family Proteins in Castration-Resistant Prostate Cancer (CRPC). *Clin Cancer Res*, **24**, 3149-3162.
29. Sharp, A., Coleman, I., Yuan, W., Sprenger, C., Dolling, D., Nava Rodrigues, D., Russo, J.W., Figueiredo, I., Bertan, C., Seed, G. *et al.* (2018) Androgen receptor splice variant-7 expression emerges with castration resistance in prostate cancer. *J Clin Invest*.
30. Han, S., Udeshi, N.D., Deerinck, T.J., Svinkina, T., Ellisman, M.H., Carr, S.A. and Ting, A.Y. (2017) Proximity Biotinylation as a Method for Mapping Proteins Associated with mtDNA in Living Cells. *Cell Chem Biol*, **24**, 404-414.
31. Cox, J. and Mann, M. (2008) MaxQuant enables high peptide identification rates, individualized p.p.b.-range mass accuracies and proteome-wide protein quantification. *Nat Biotechnol*, **26**, 1367-1372.

Supplementary Figure S1

A.

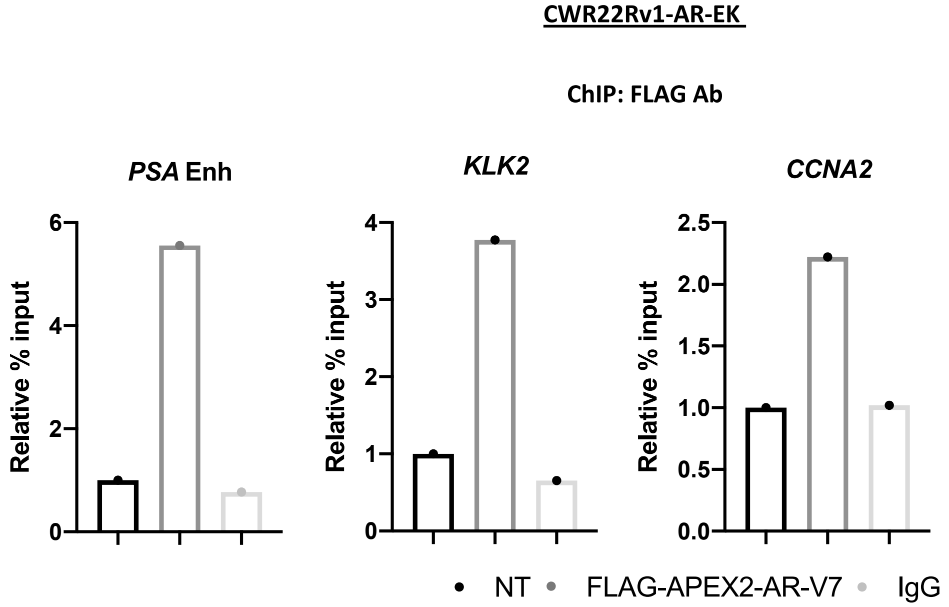

B.

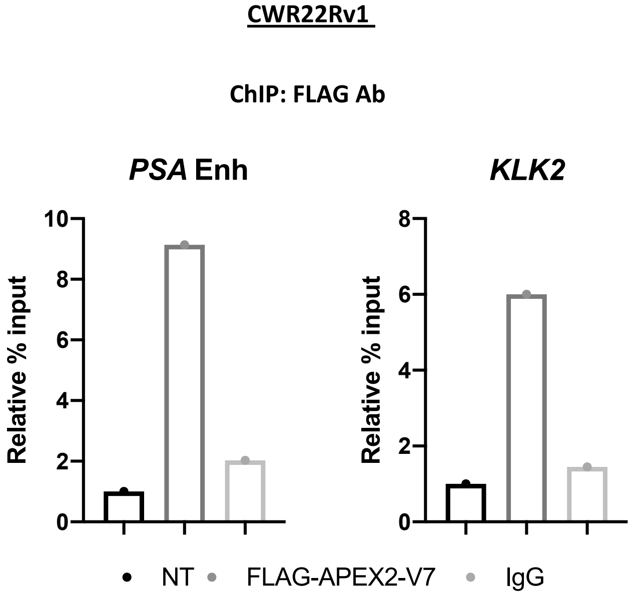

Supplementary Figure S2

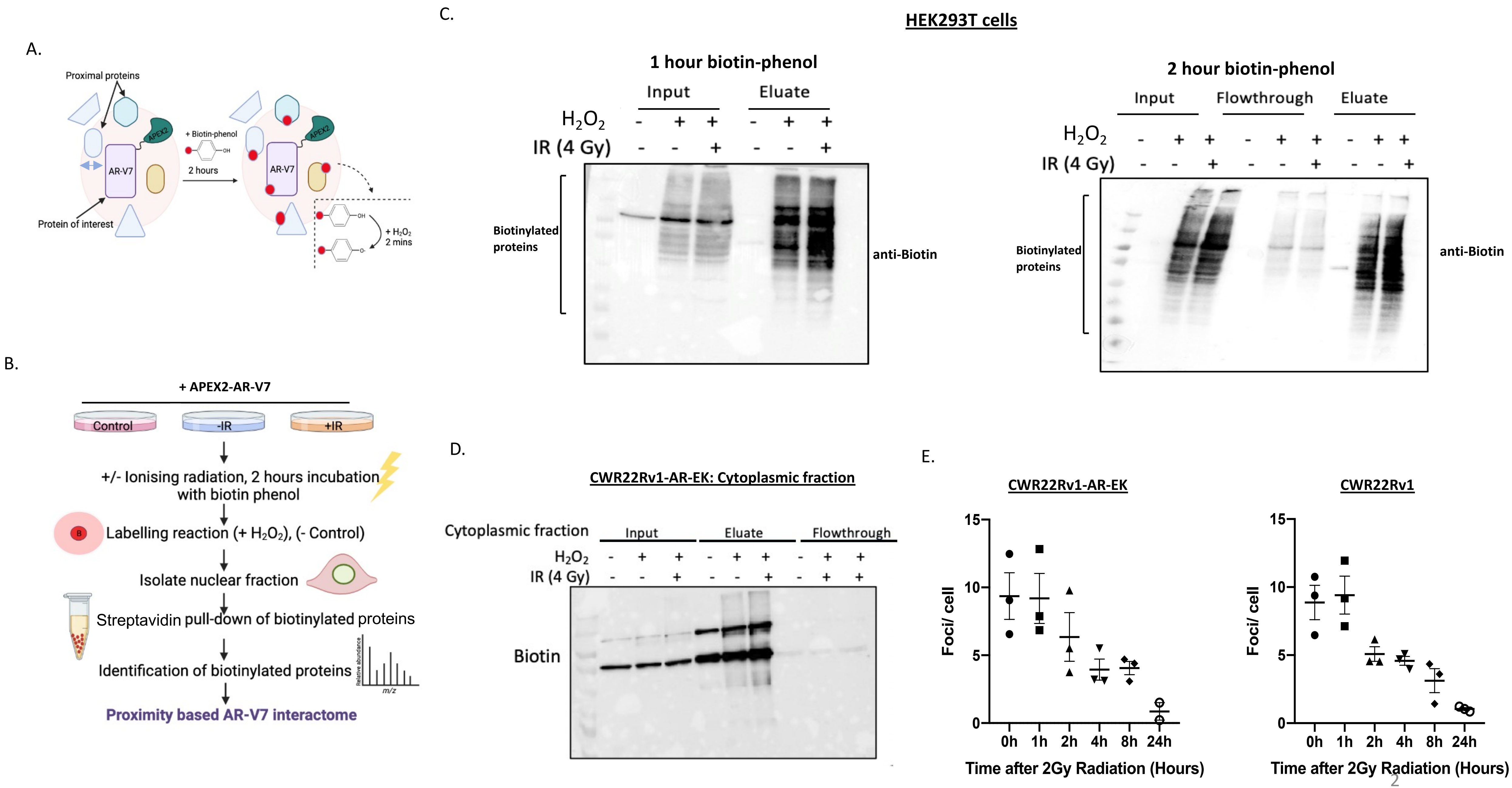

Supplementary Figure S3

A.

Table of number of proteins identified by mass spectrometry for each proximity labelling replicate

CONTROL	No. of proteins	- IR	No. of proteins	+ IR	No. of proteins
iBAQ_n2	33	iBAQ_n2	265	iBAQ_n2	427
iBAQ_n3	64	iBAQ_n3	444	iBAQ_n3	436
iBAQ_n4	166	iBAQ_n4	484	iBAQ_ n4	510

C.

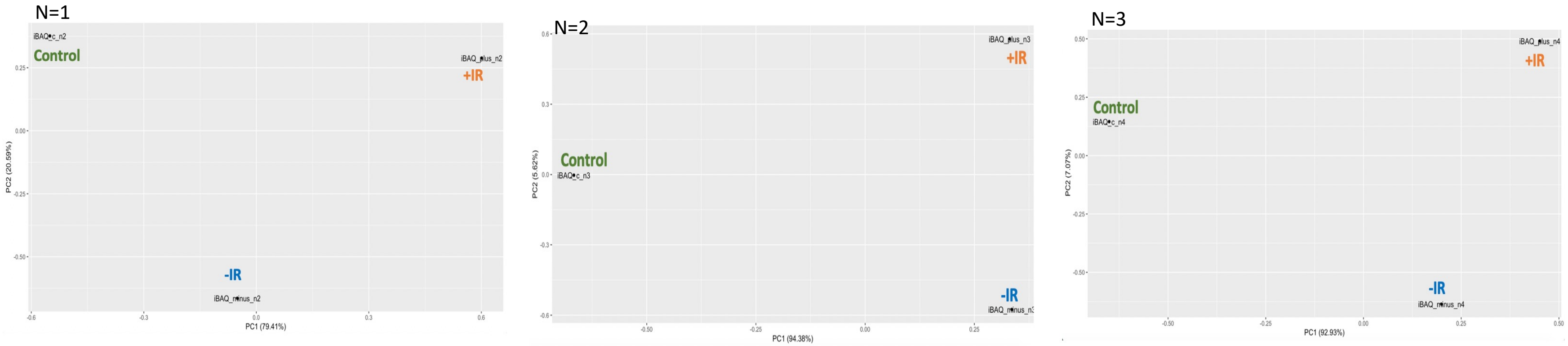

B.

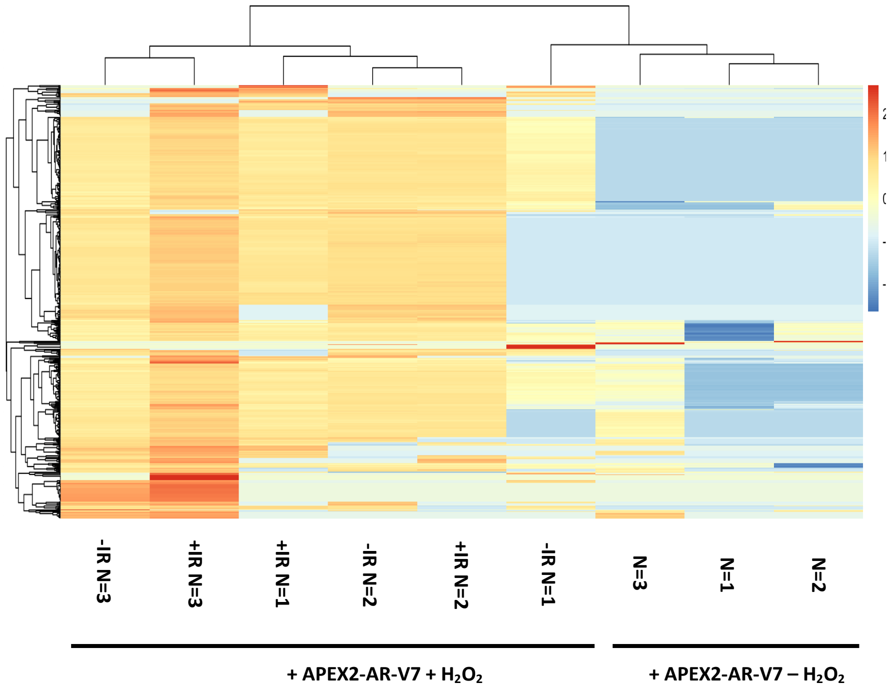

D.

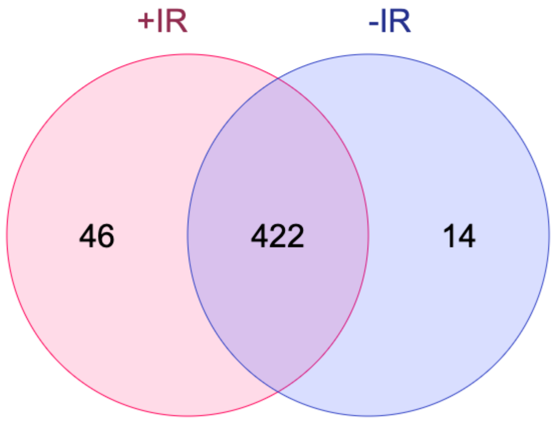

E.

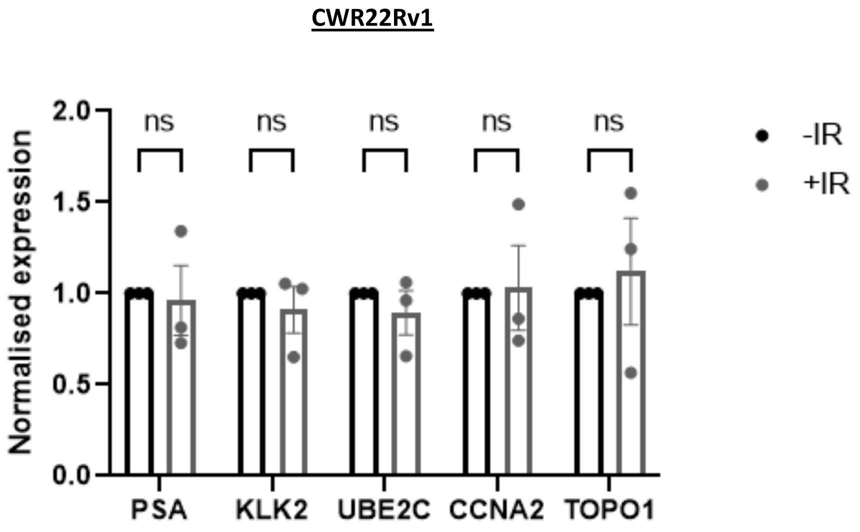

Supplementary Figure S4

A.

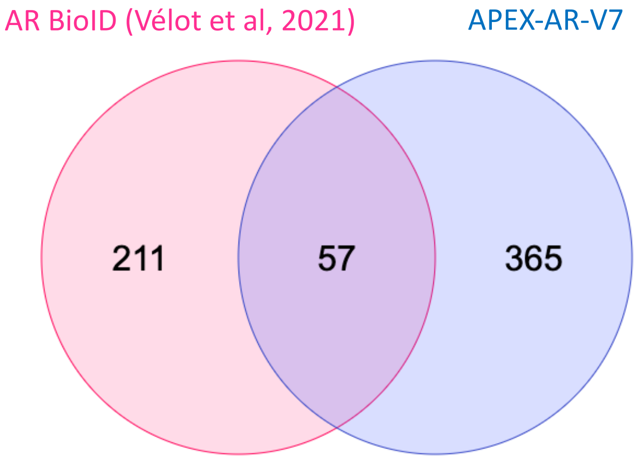

Shared interactome					
ACTL6A	DPF2	MED17	RBM10	SMARCA2	TNRC18
AR	EP400	MED4	RBM17	SMARCA4	U2SURP
BRD8	GTF3C4	MEF2D	RBM27	SMARCB1	UBN2
CDC73	HCFC1	MEPCE	RCOR3	SMARCC1	WDR5
CHD4	IK	NCOR1	RNF2	SMARCC2	YLPM1
CHERP	KMT2A	NFIX	RNF20	SMARCD1	ZC3H4
CPSF6	KMT2D	NUDT21	RNF40	SMARCD2	ZNF217
CPSF7	MBD2	OGT	RPRD2	SMARCE1	
DDX42	MDC1	PBRM1	SART1	SMCHD1	
DMAP1	MED12	RAVER1	SIN3A	TLE3	

B.

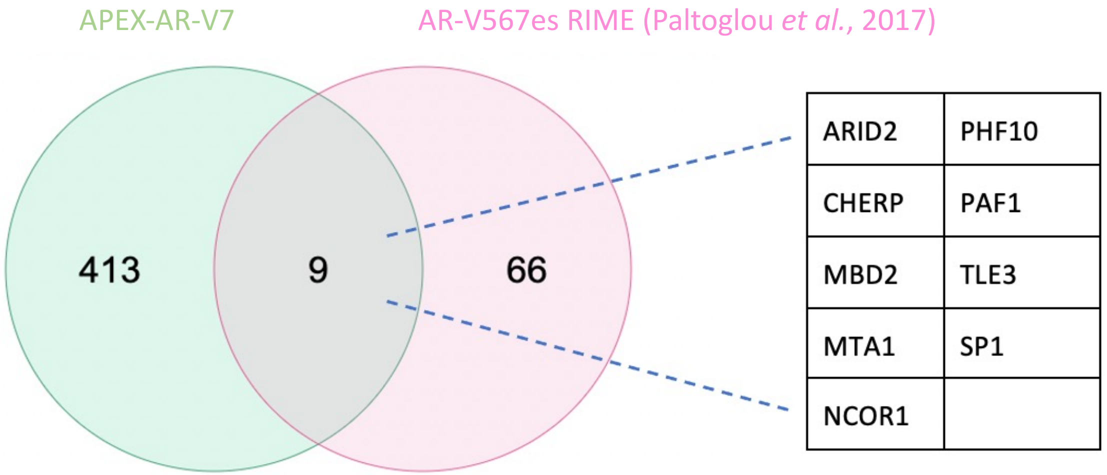

ARID2	PHF10
CHERP	PAF1
MBD2	TLE3
MTA1	SP1
NCOR1	

C.

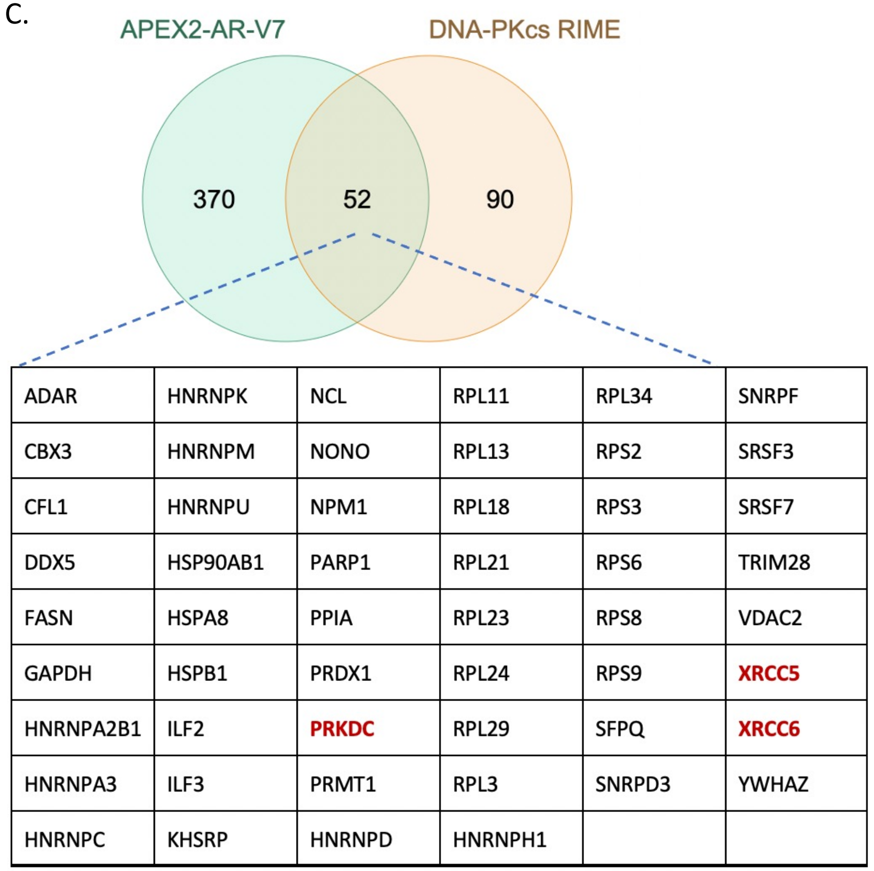

ADAR	HNRNPK	NCL	RPL11	RPL34	SNRPF
CBX3	HNRNPM	NONO	RPL13	RPS2	SRSF3
CFL1	HNRNPU	NPM1	RPL18	RPS3	SRSF7
DDX5	HSP90AB1	PARP1	RPL21	RPS6	TRIM28
FASN	HSPA8	PPIA	RPL23	RPS8	VDAC2
GAPDH	HSPB1	PRDX1	RPL24	RPS9	**XRCC5**
HNRNPA2B1	ILF2	**PRKDC**	RPL29	SFPQ	**XRCC6**
HNRNPA3	ILF3	PRMT1	RPL3	SNRPD3	YWHAZ
HNRNPC	KHSRP	HNRNPD	HNRNPH1		

Supplementary Figure S5

A.

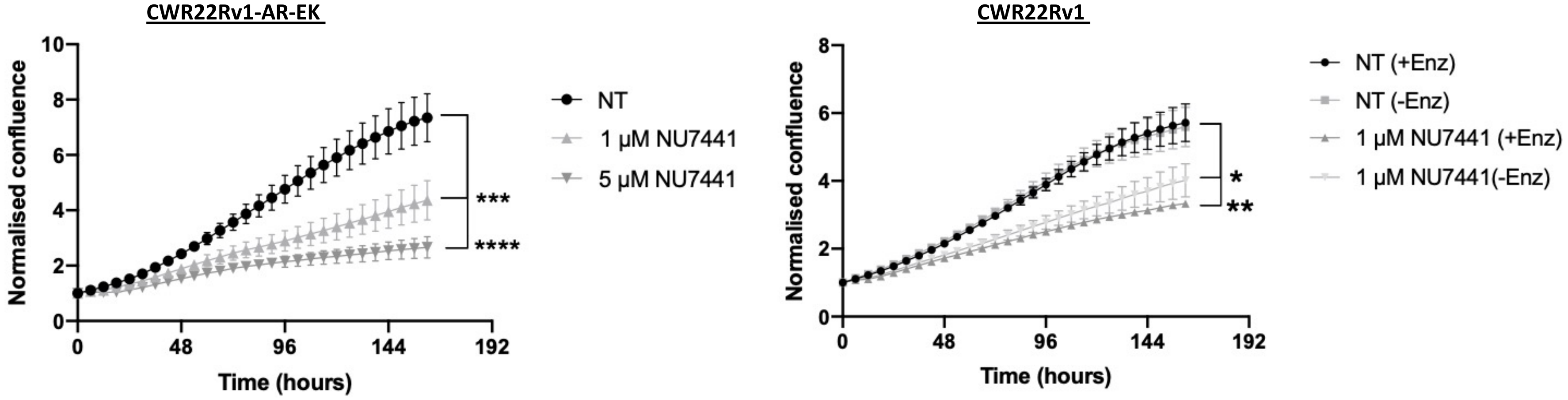

B.

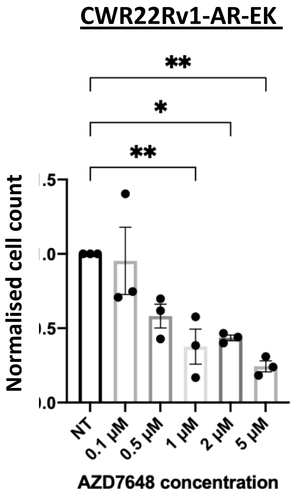

C.

DNA-PK Inhibitor	GI_{50} (μM)
NU7441	1.8
NU5455	1.4
AZD7648	2.8

Supplementary Figure S6

CWR22Rv1-AR-EK

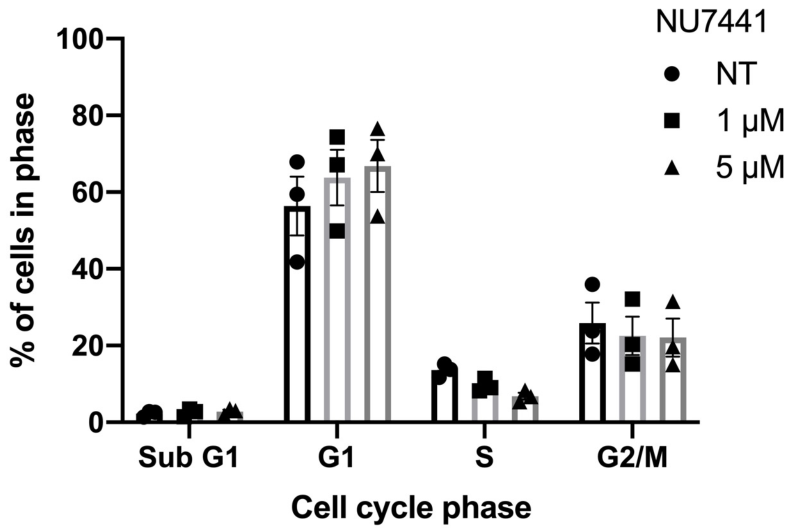

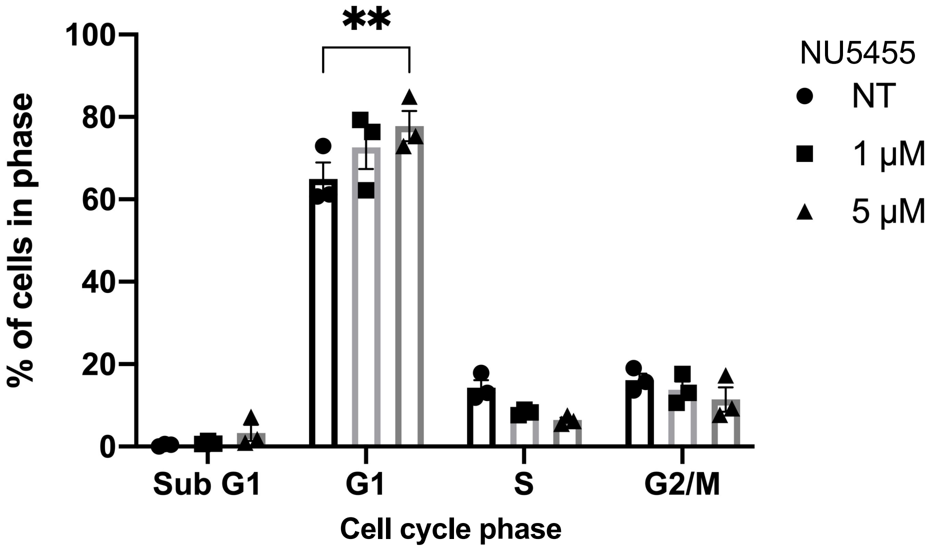

Supplementary Figure S7

A.

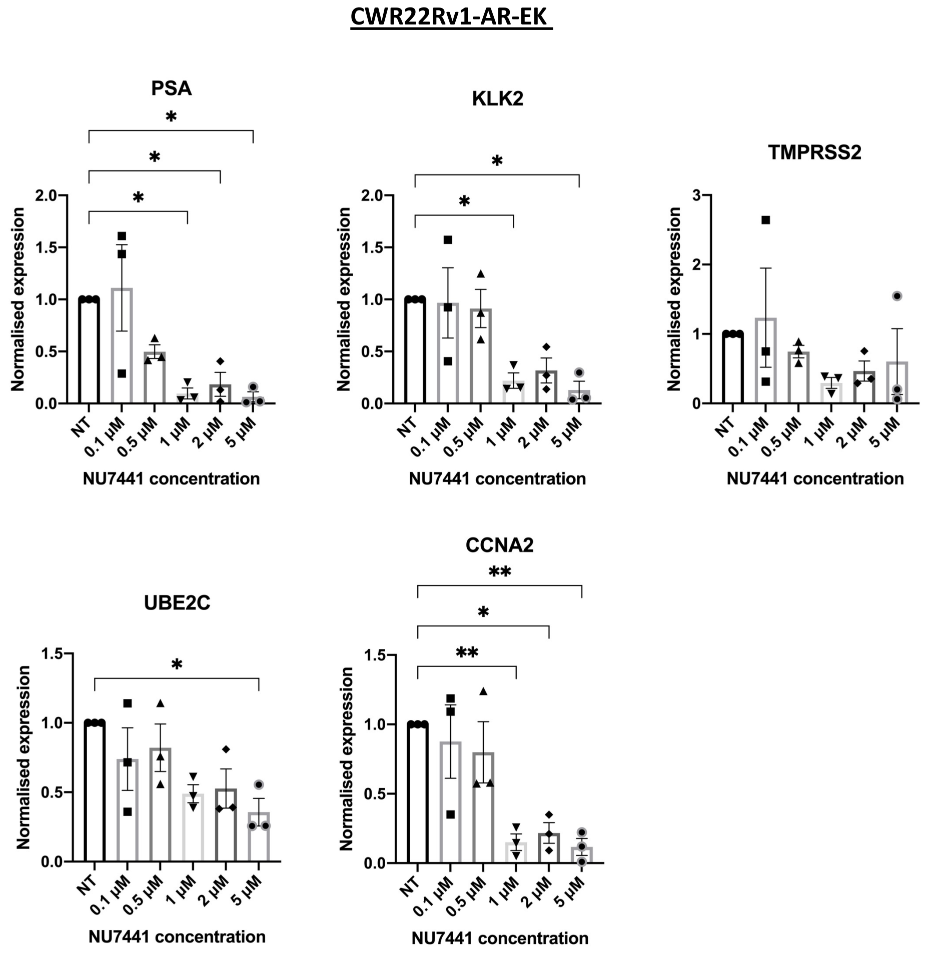

B.

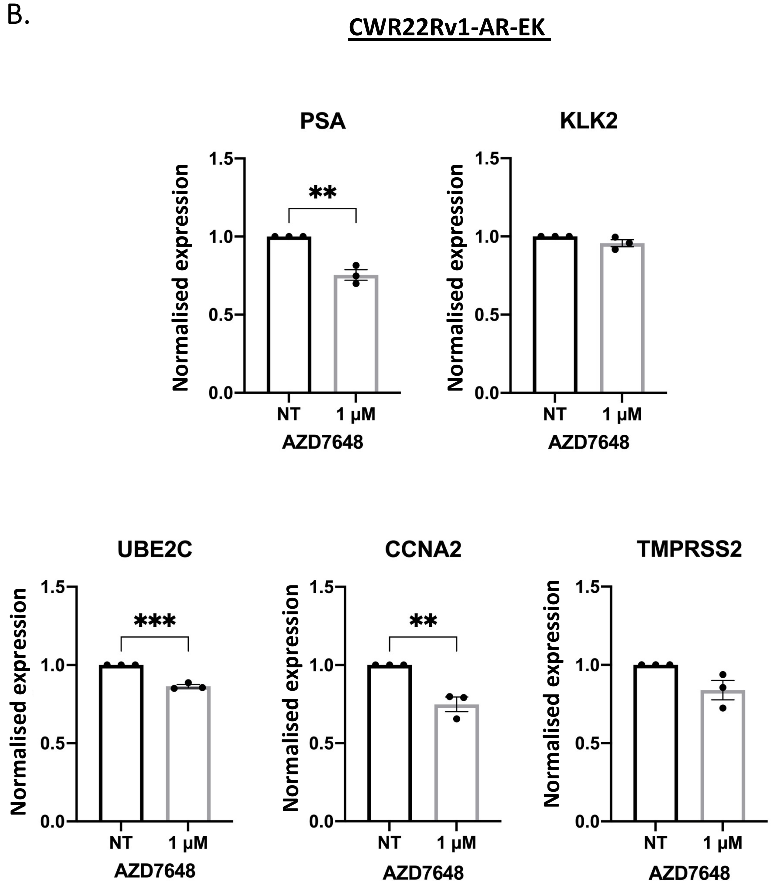

Supplementary Figure S8

A.

CWR22Rv1

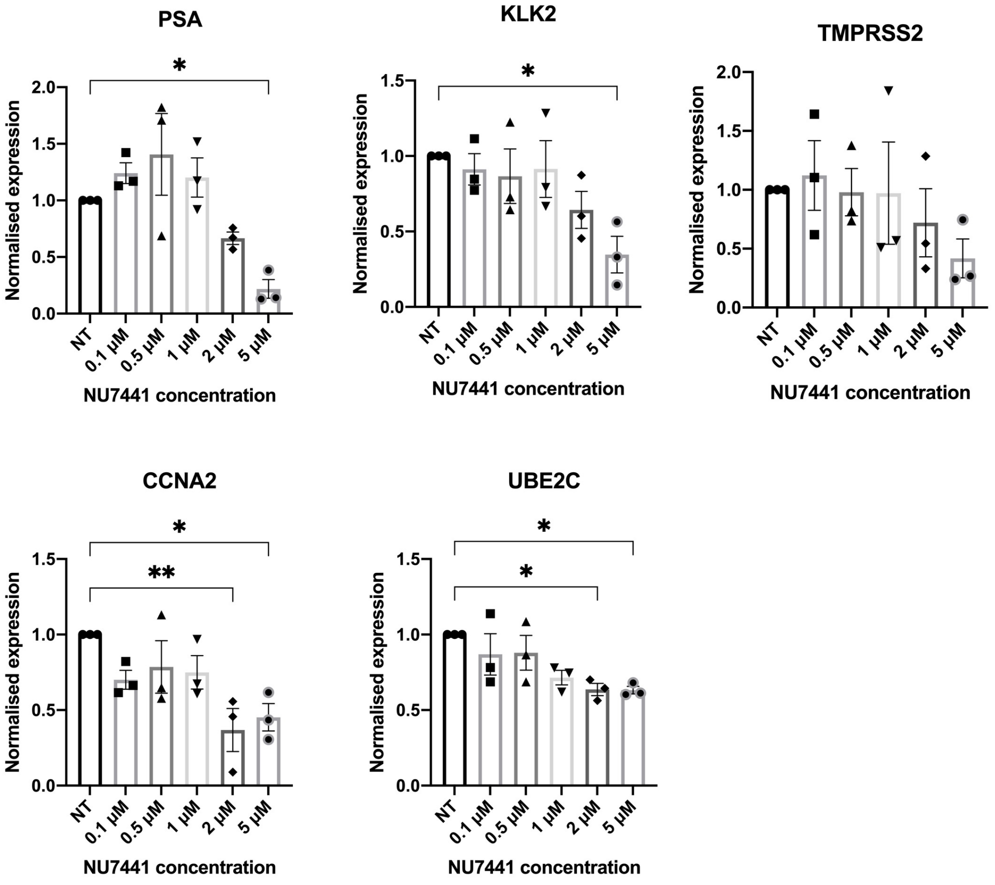

B.

VCaP

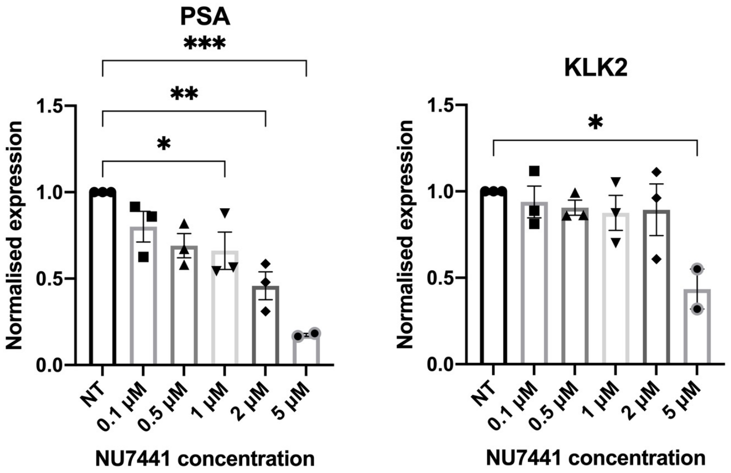

C.

VCaP

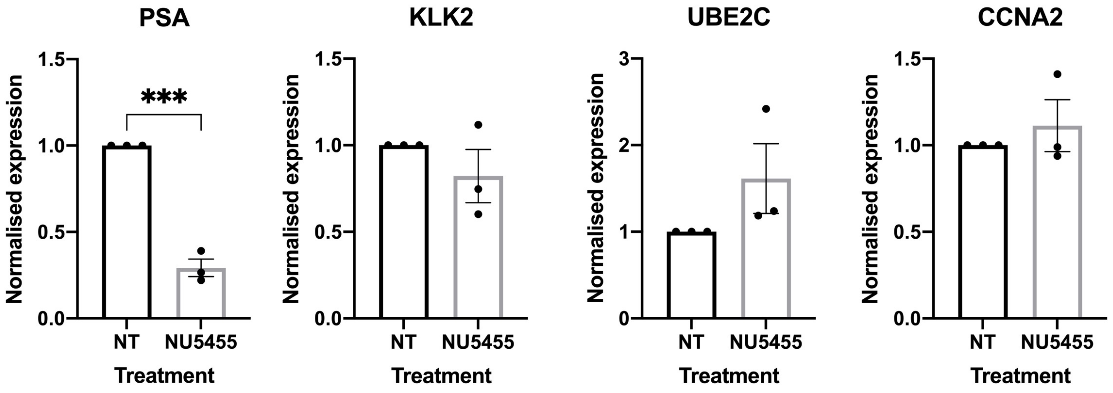

Supplementary Figure S9

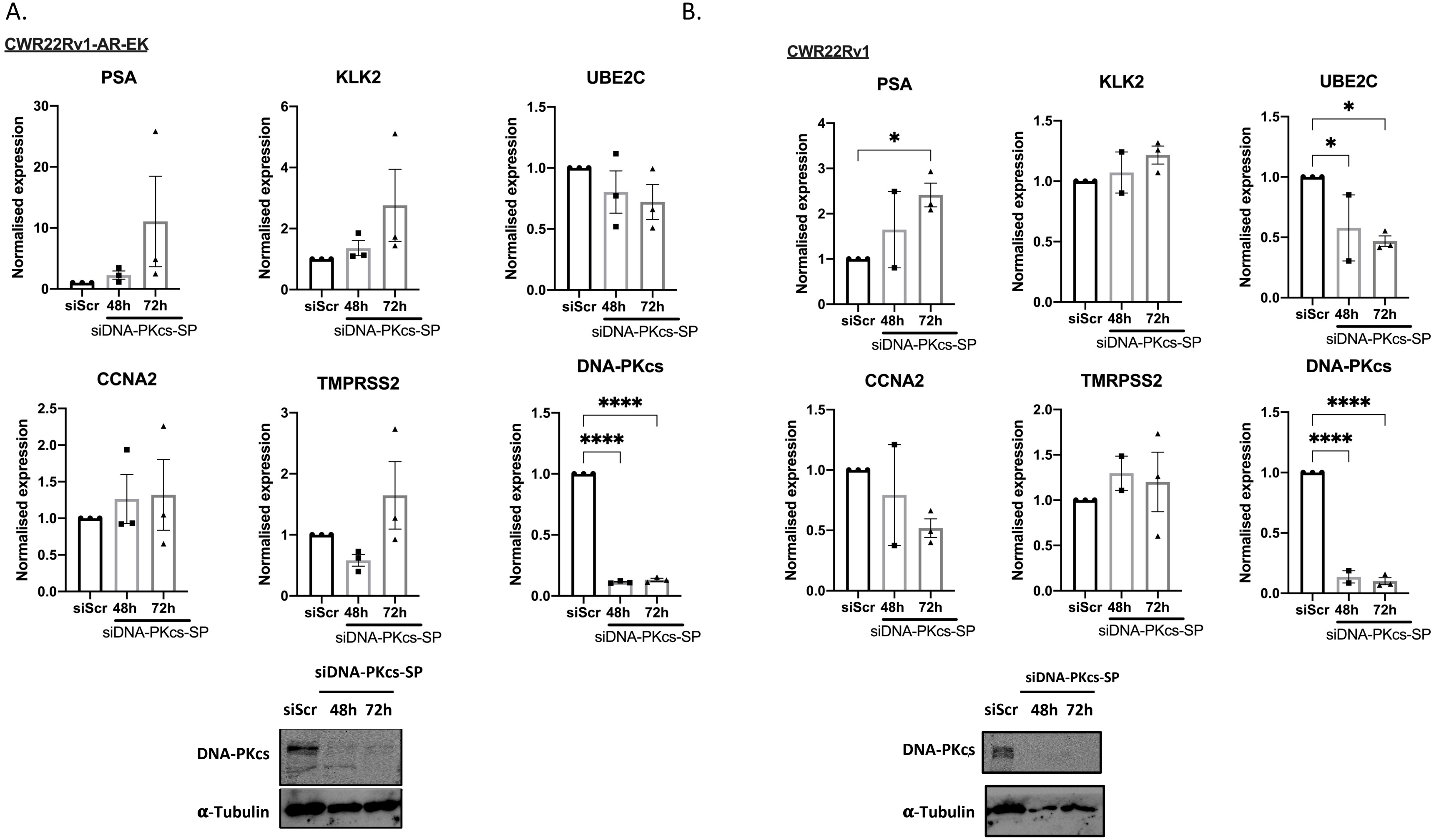

Supplementary Figure S10

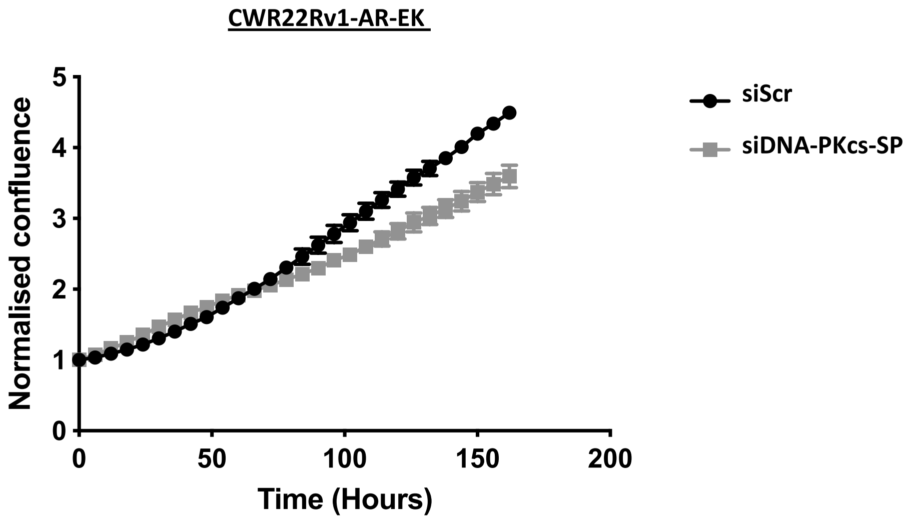

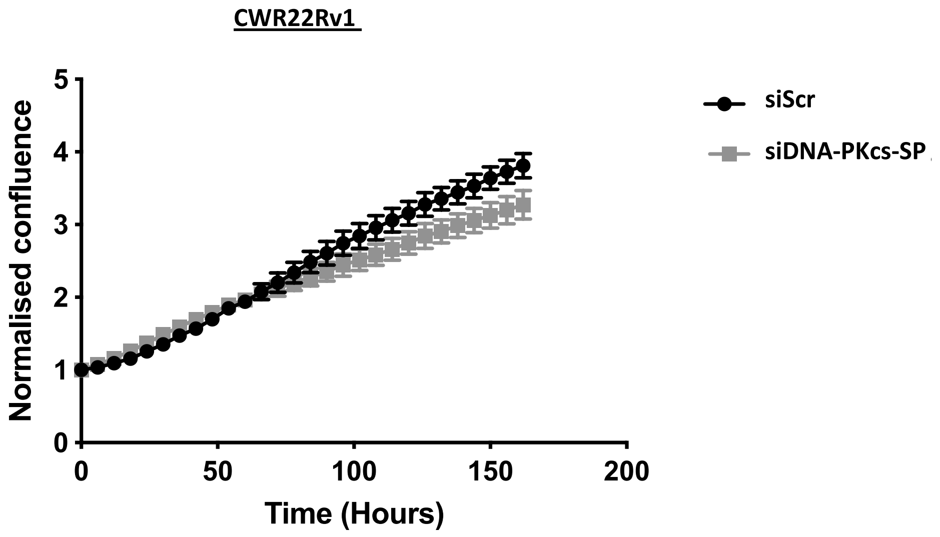

Supplementary Figure S11

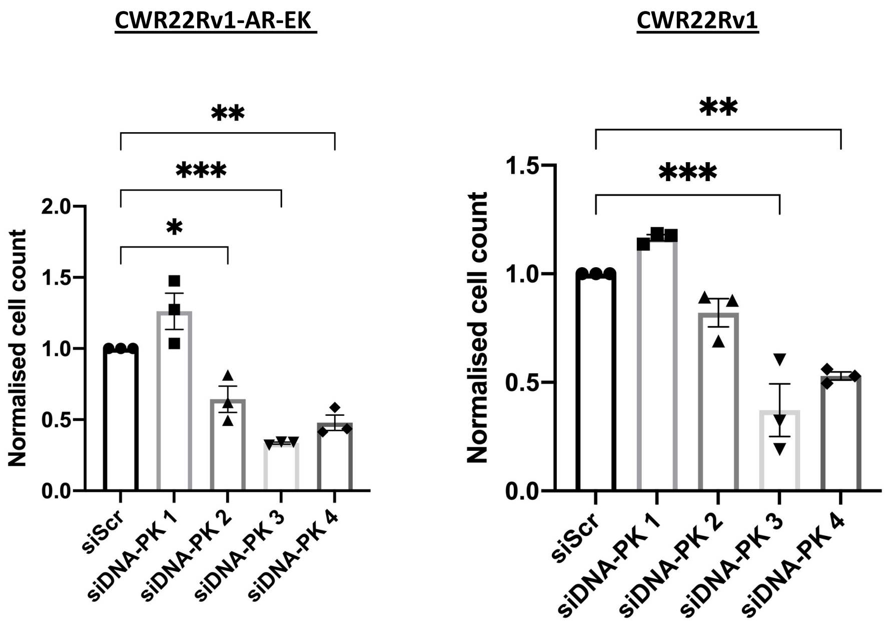

Supplementary Figure S12

A.

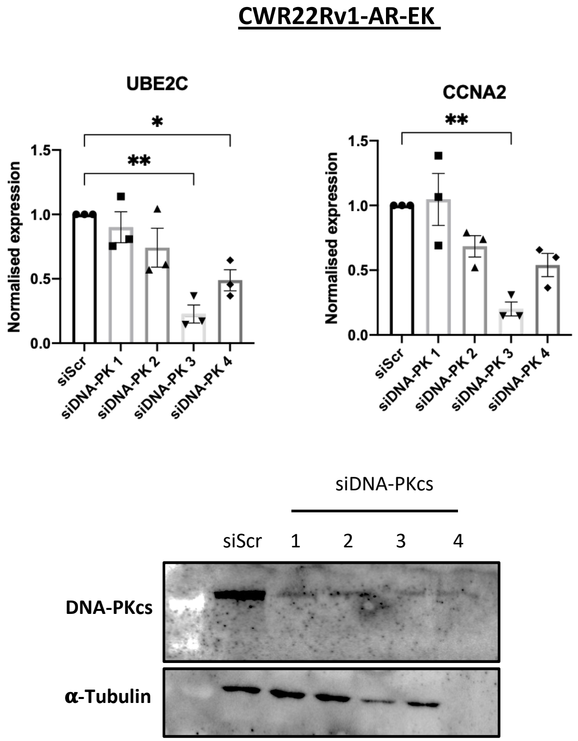

B.

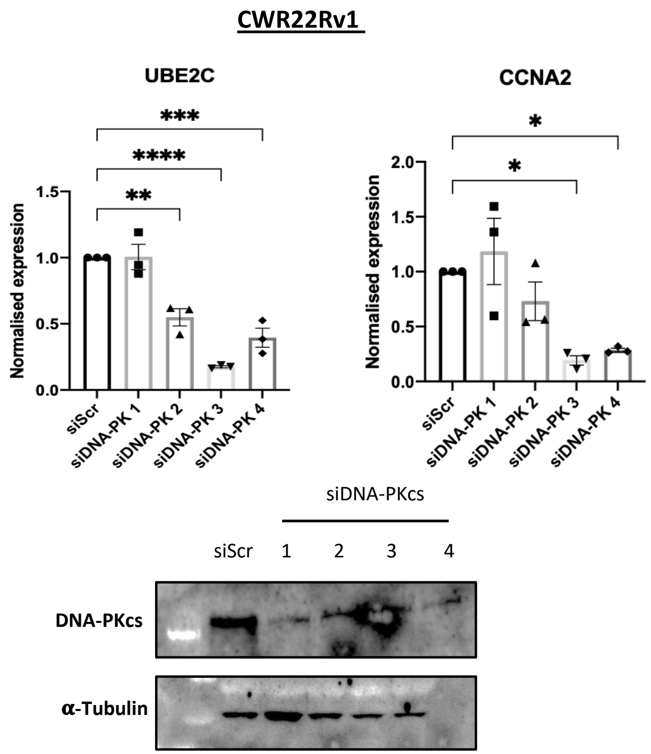

Supplementary Figure S13

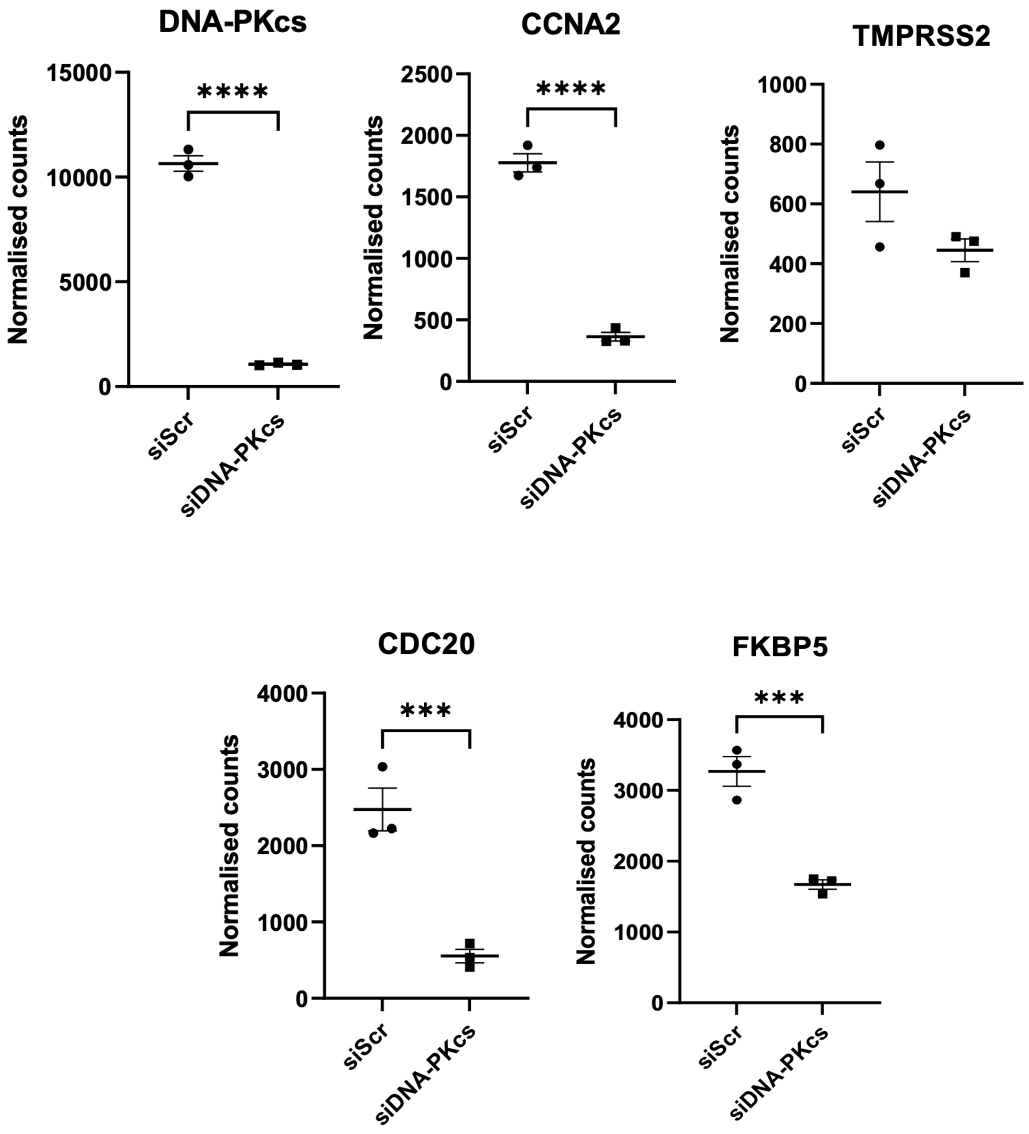

Supplementary Figure S14

A.

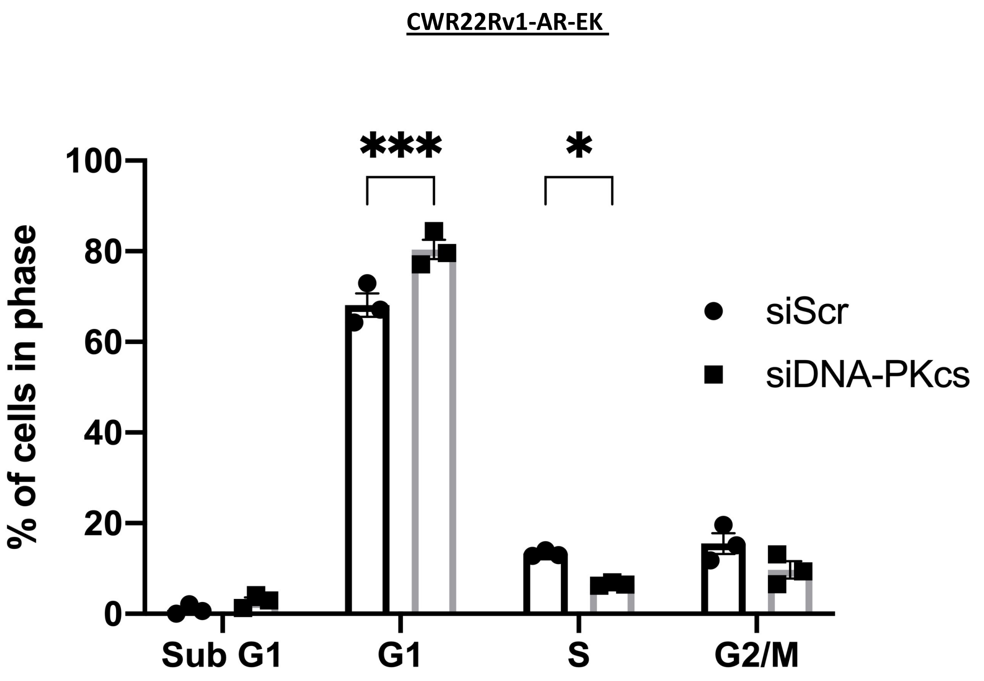

B.

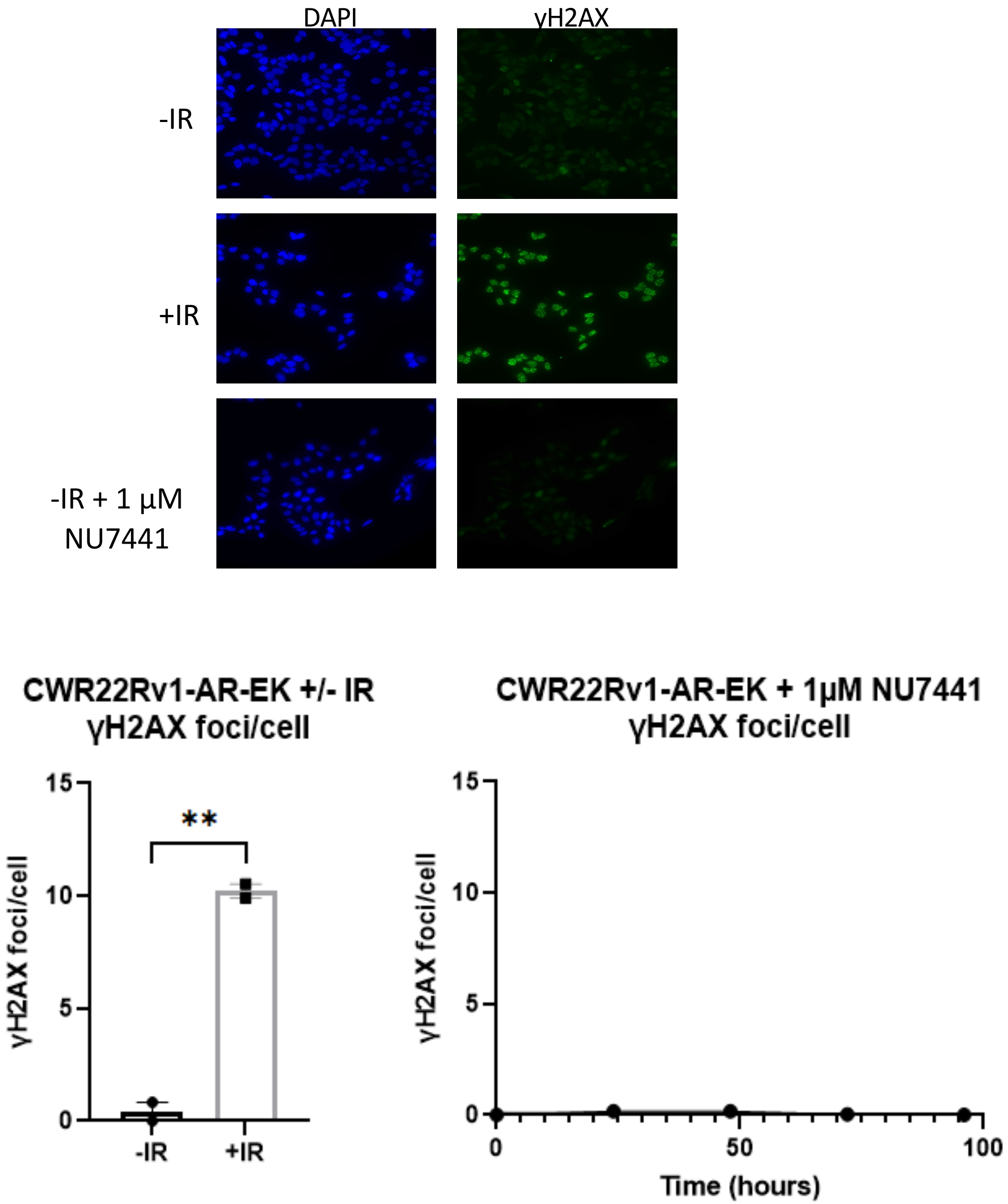

Supplementary Figure S15

B.

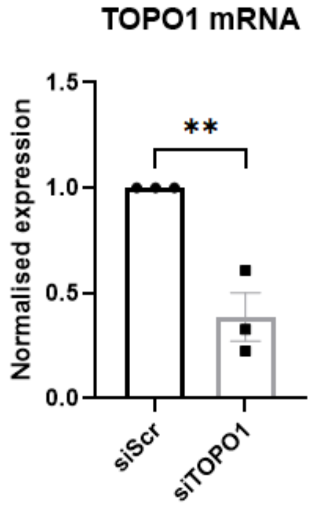

A.

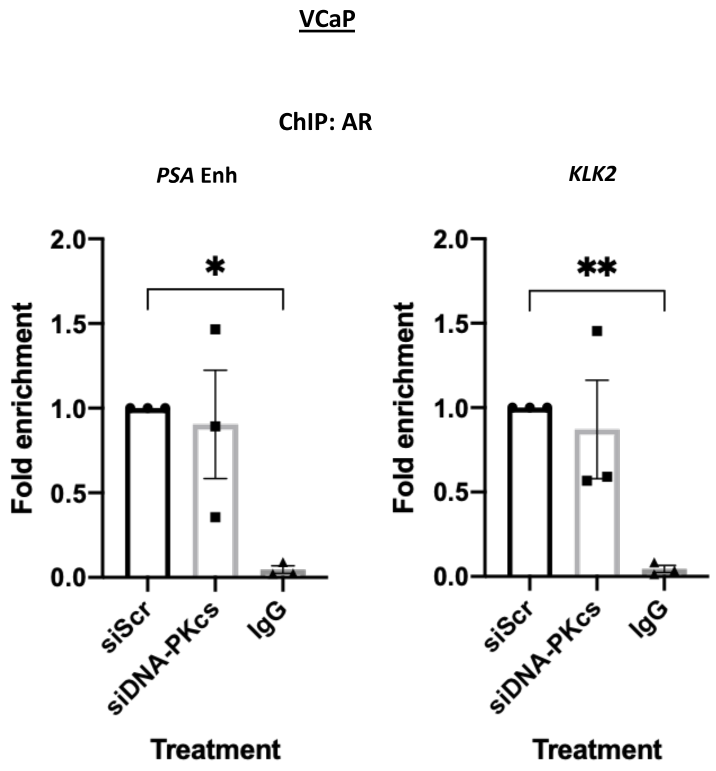

C.

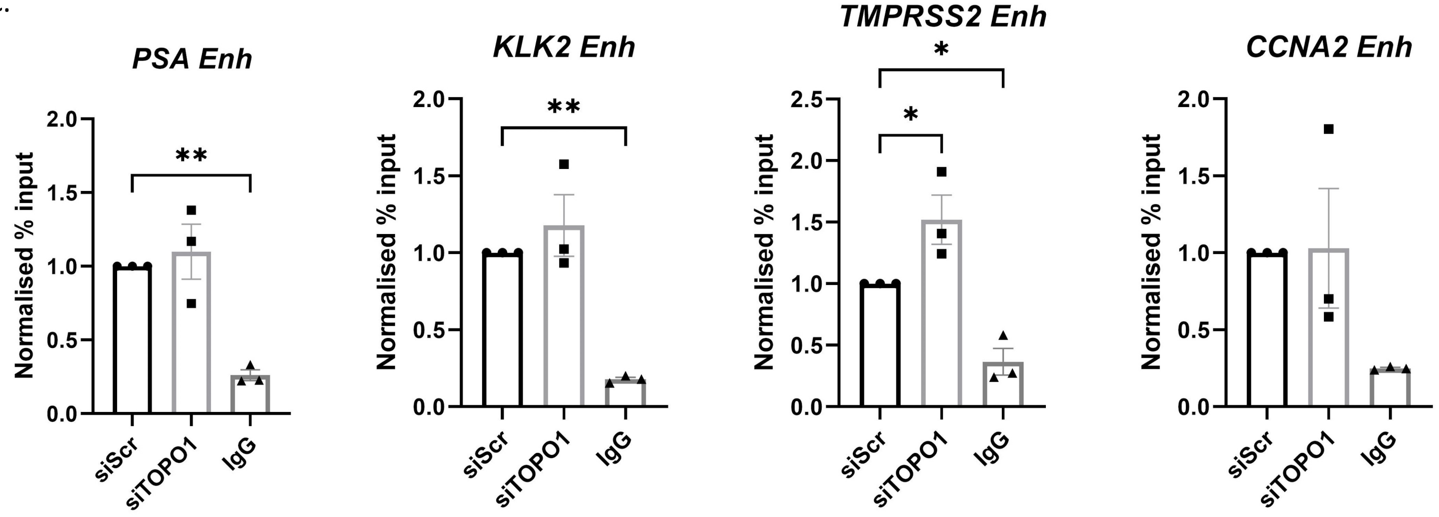

Supplementary Figure S16

A.

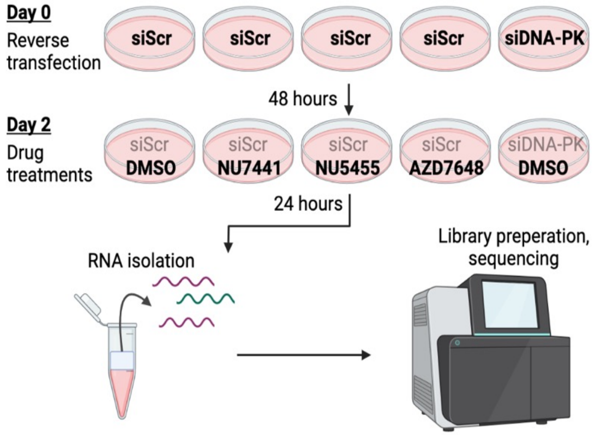

B.

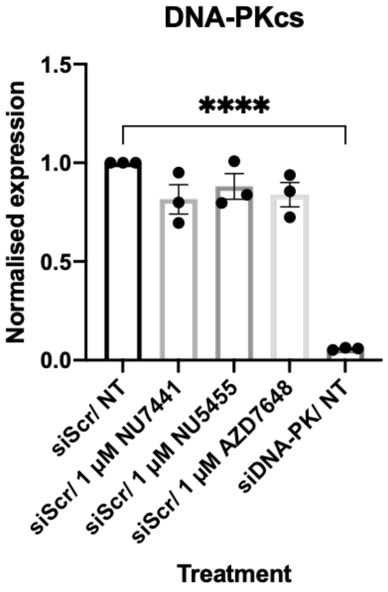

C.

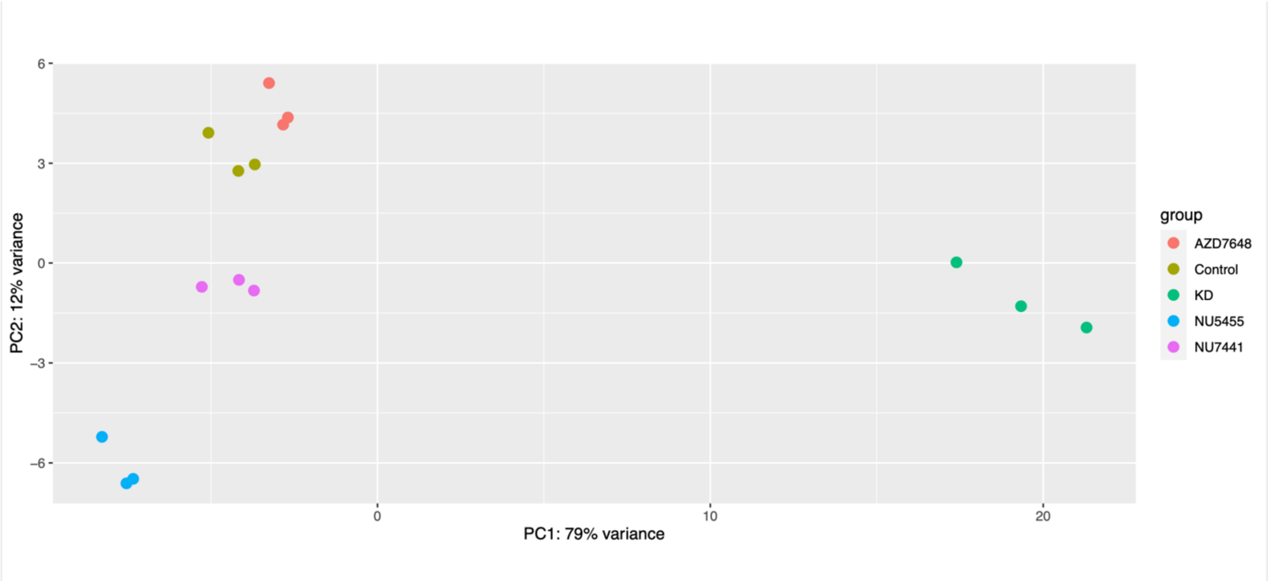

Supplementary Figure S17

A.

Treatment	No. of DEGs	No. of significant DEG (padj < 0.05)	No. of significant DEGS FC >1.5/<-1.5
**AZD7648**	16632	372	44
**NU7441**	14050	691	27
**NU5455**	18569	6154	1195
**siDNA-PKcs**	21792	9210	3827

C.

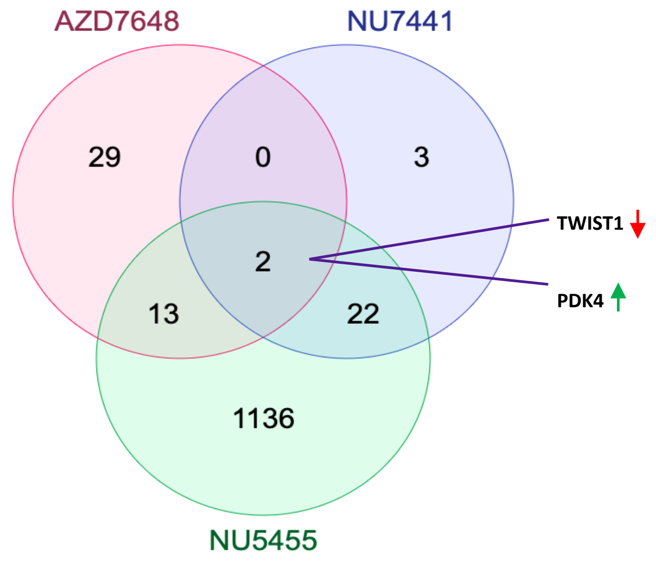

B.

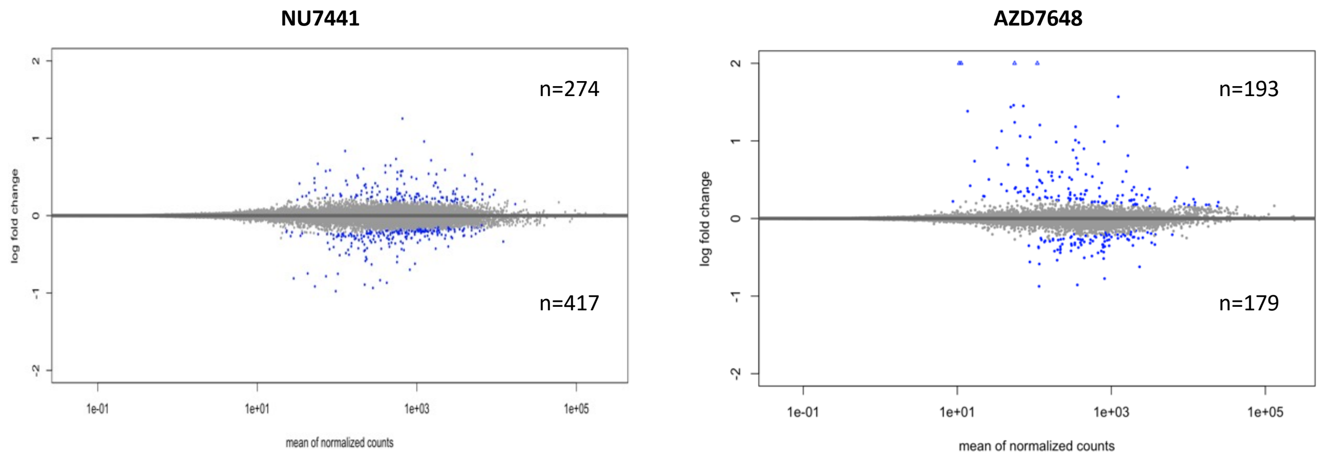

D.

Treatment	Number of downregulated pathways (nominal p value <0.05)	Number of upregulated pathways (nominal p value <0.05)
siDNA-PKcs	34	8
NU5455	48	1
NU7441	30	3
AZD7648	9	7

Common down-regulated:

p53
DNA replication
Cell cycle

Supplementary Figure S18

A.

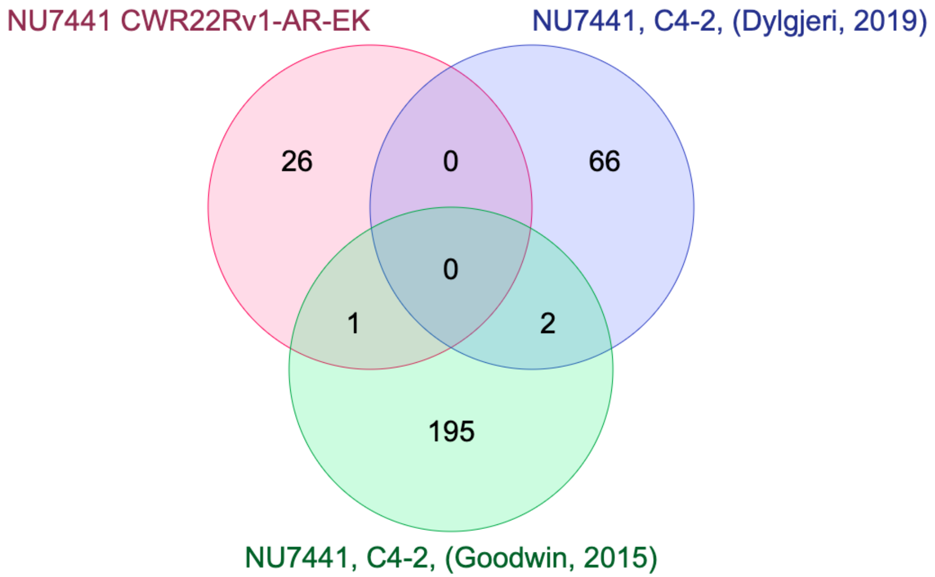

B.

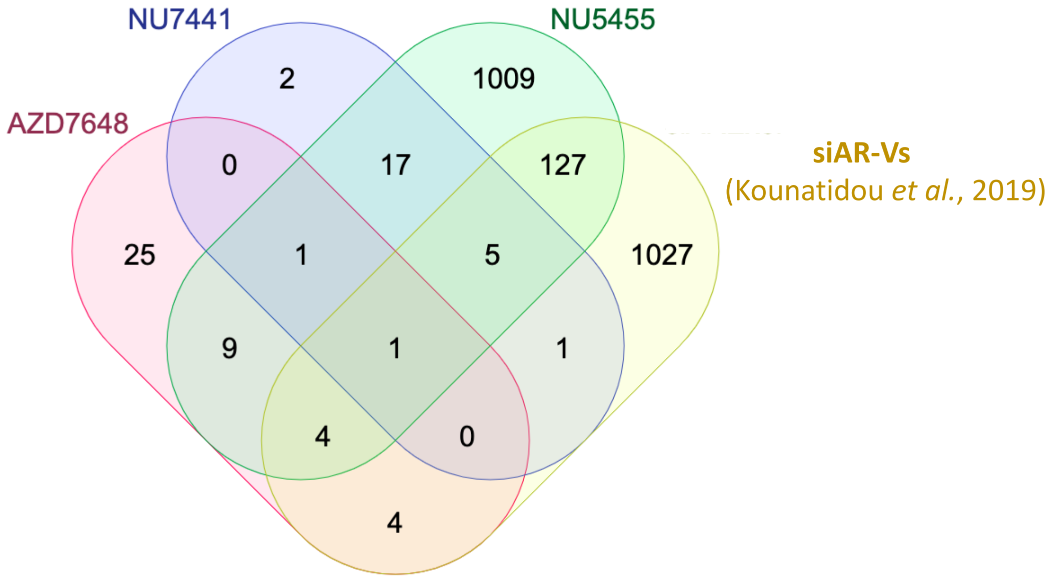

Supplementary Figure S19

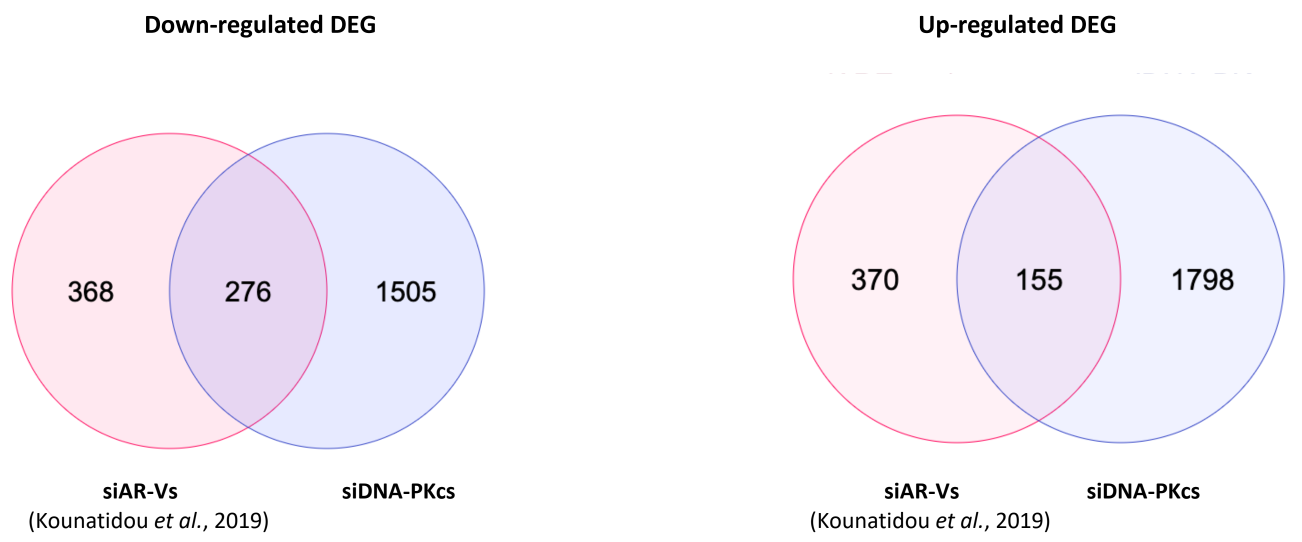

Supplementary Figure S20

A.

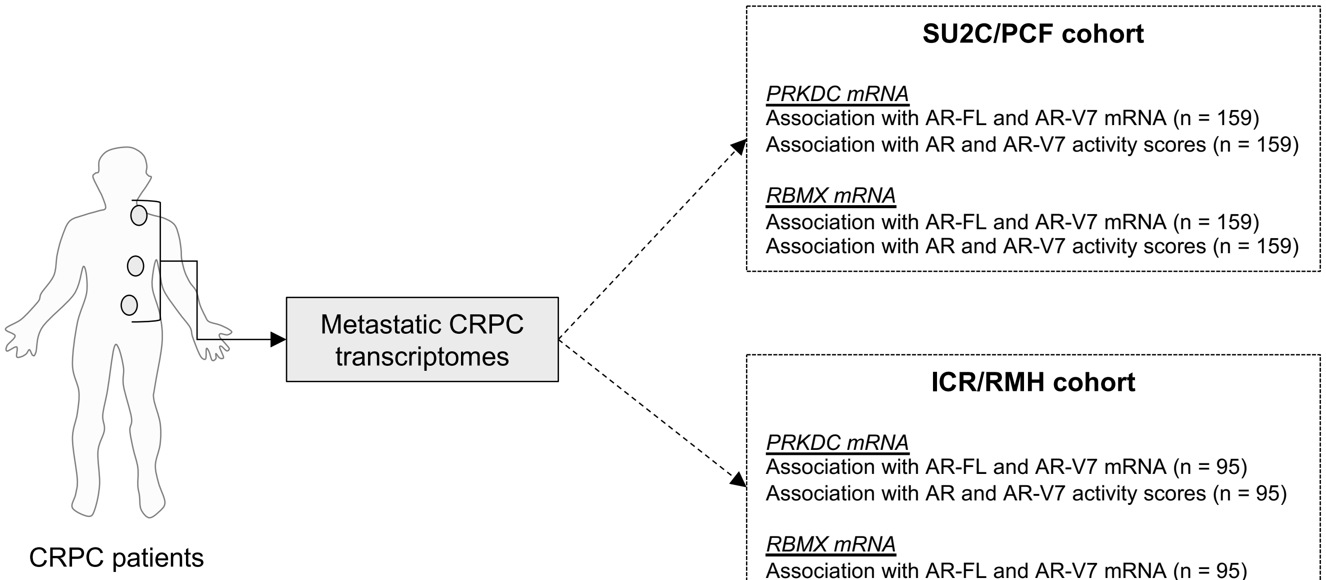

B. C.

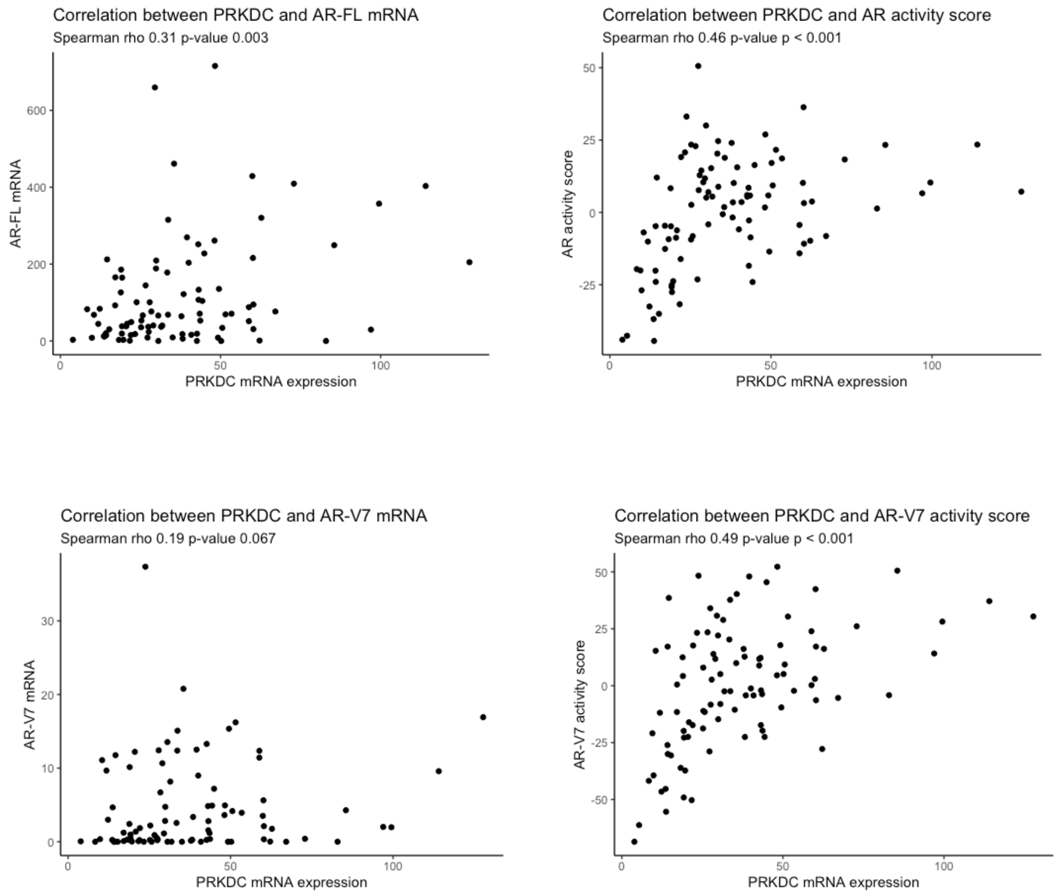

ICR/RMH (n=95)

Supplementary Figure S21

A.

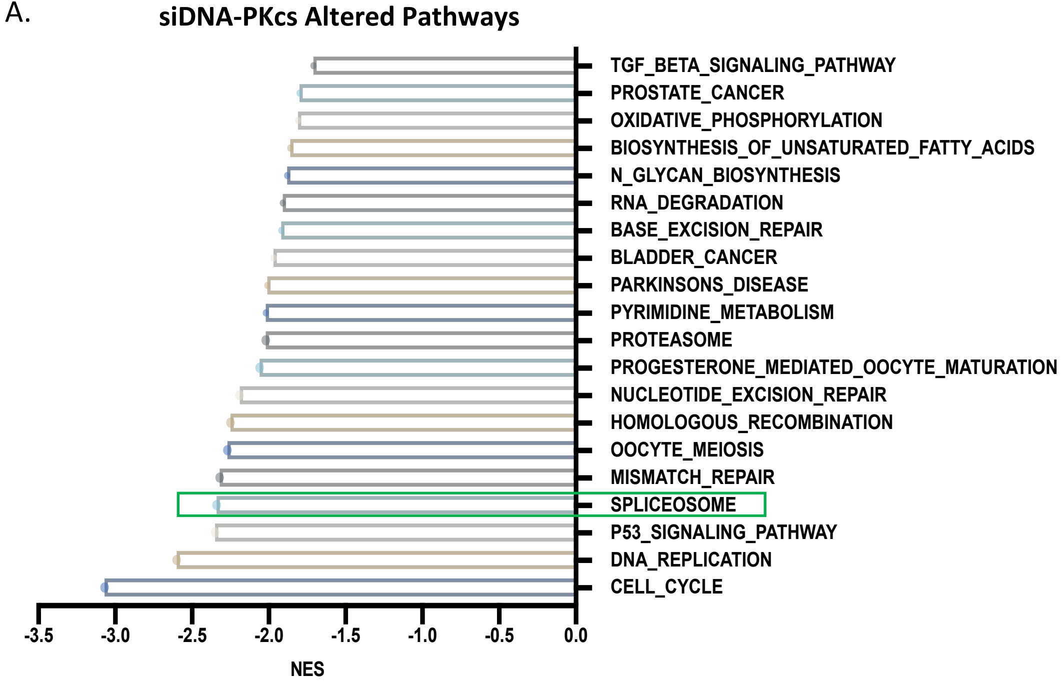

B.

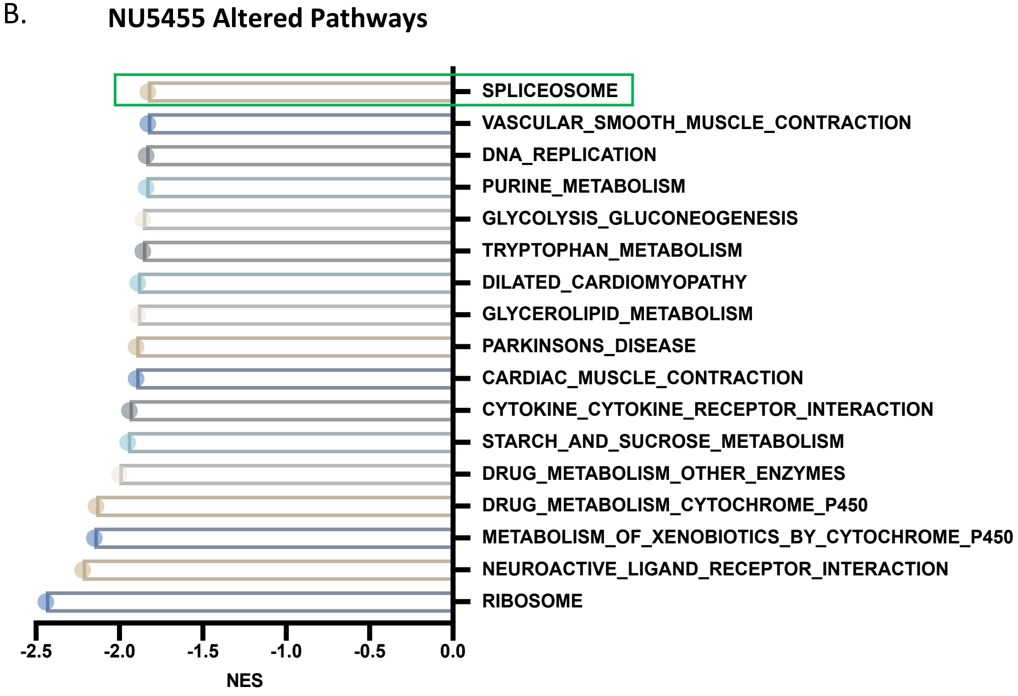

siDNA-PKcs

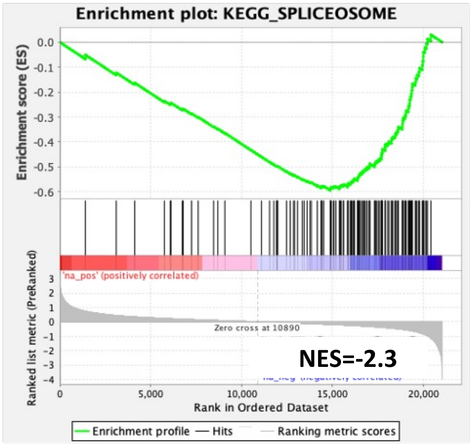

NU5455

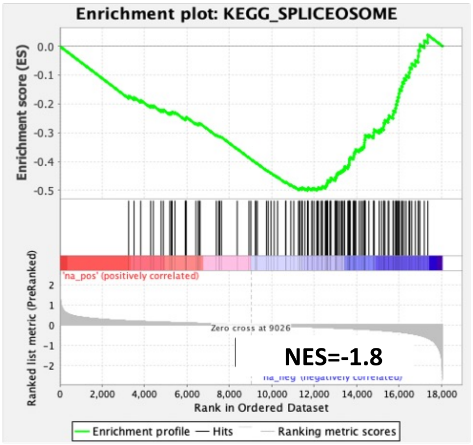

Supplementary Figure S22

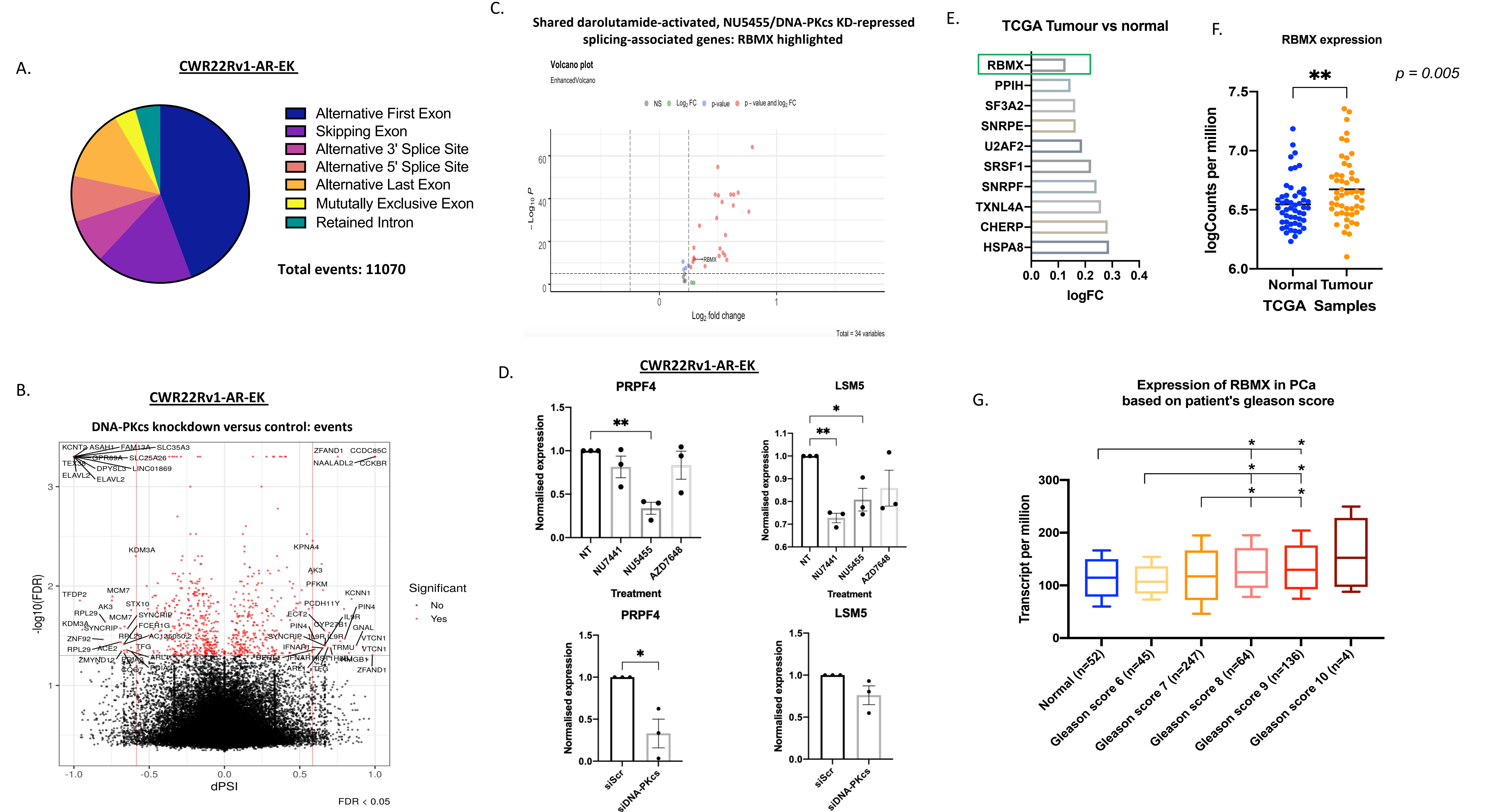

Supplementary Figure S23

A.

MSKCC; Taylor *et al*., 2010

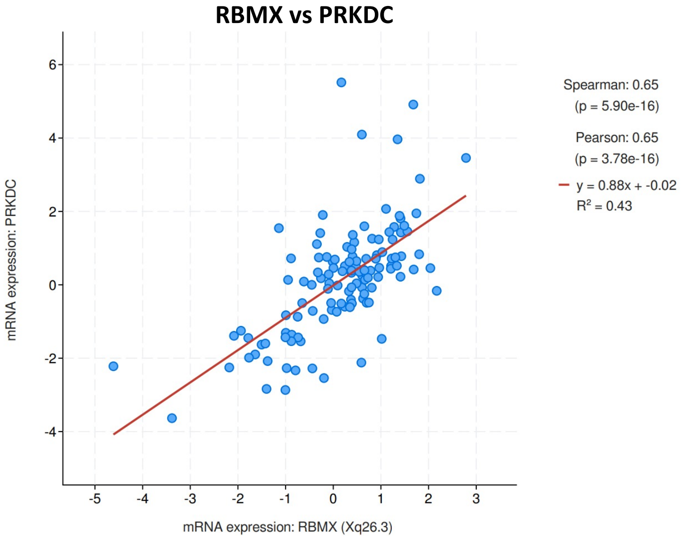

B.

TCGA; *Cell* 2015

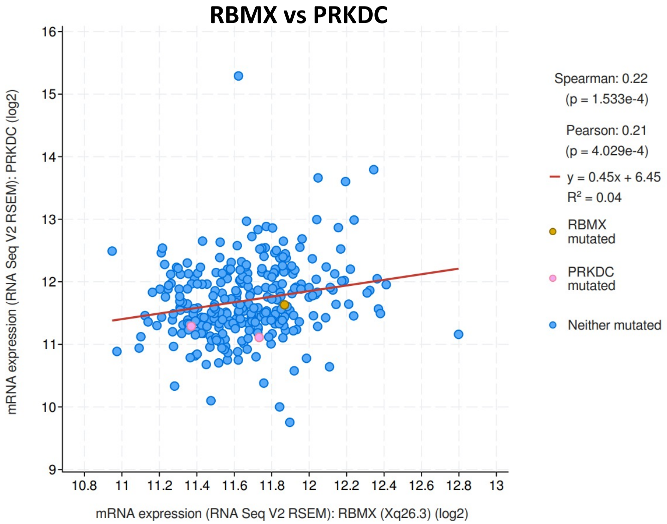

Supplementary Figure S24

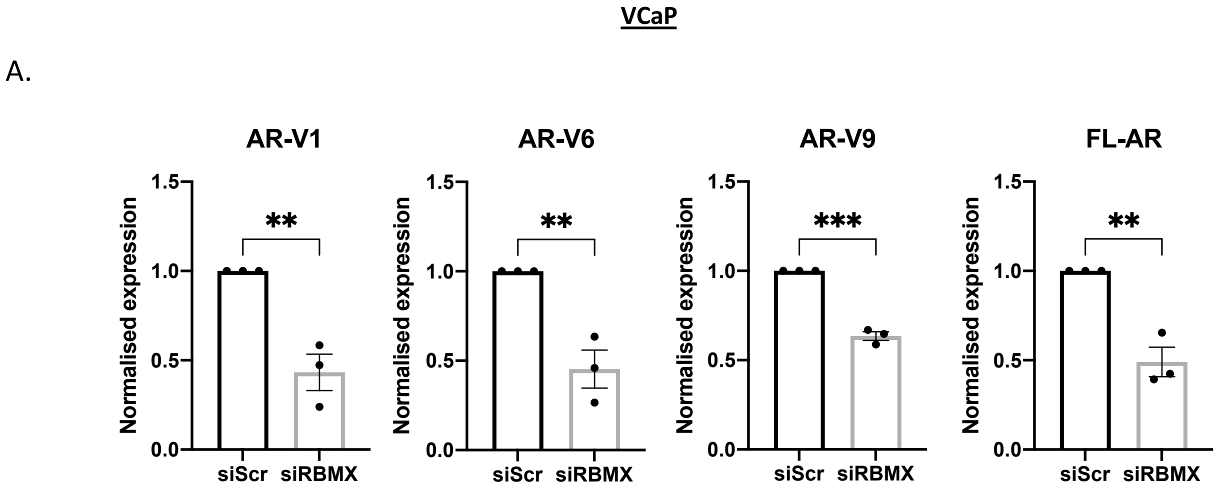

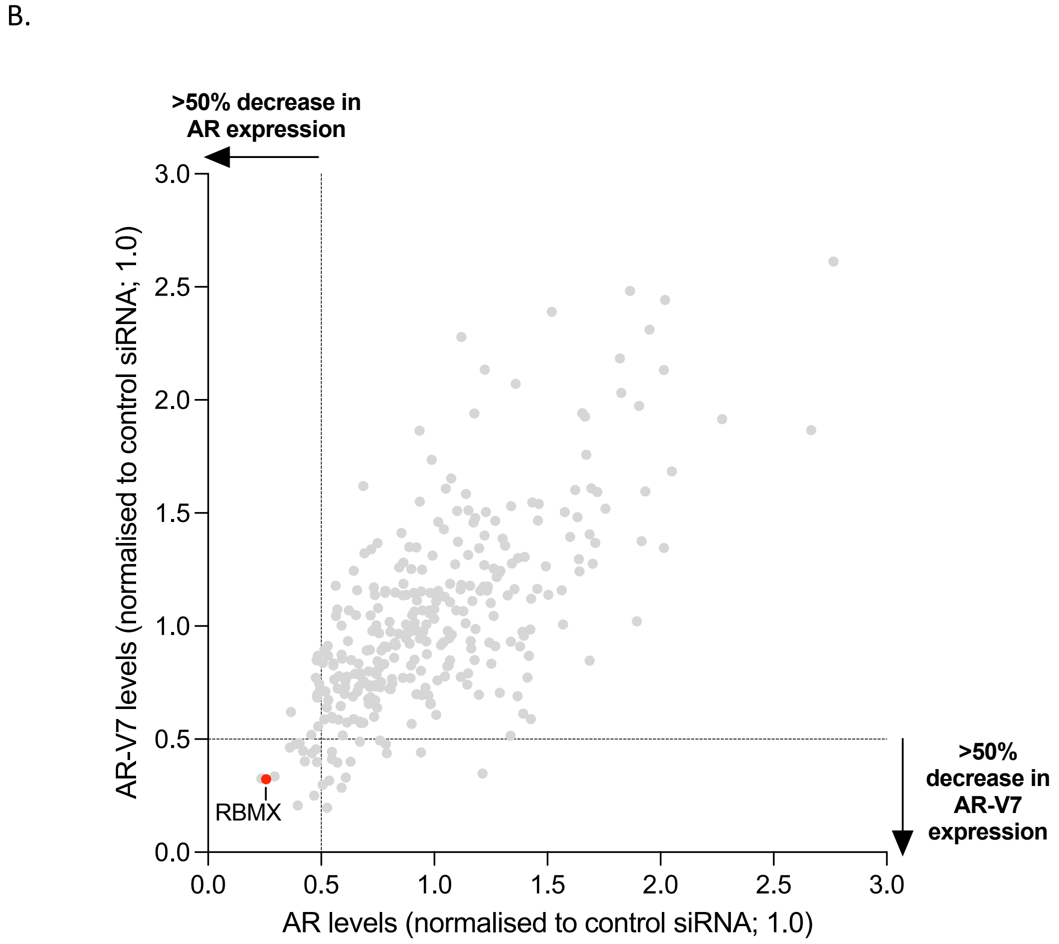

Supplementary Figure S25

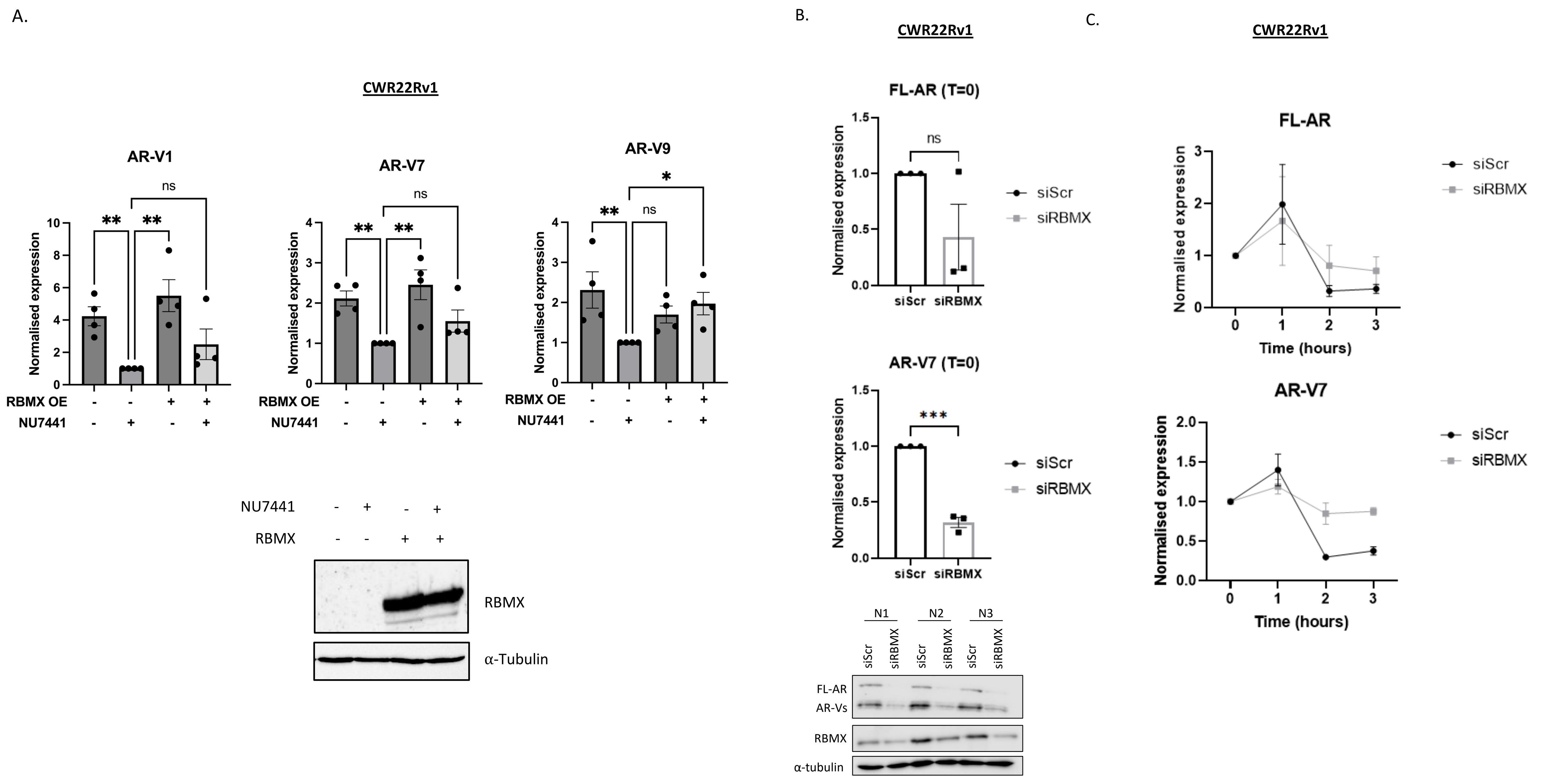

Supplementary Figure S26

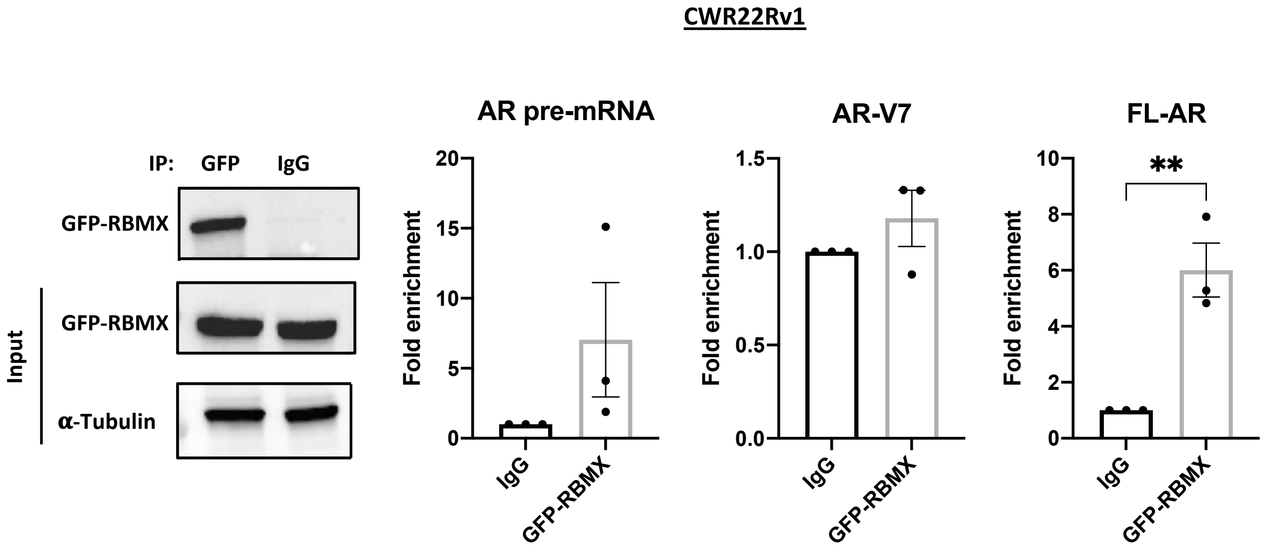

Supplementary Figure S27

A.

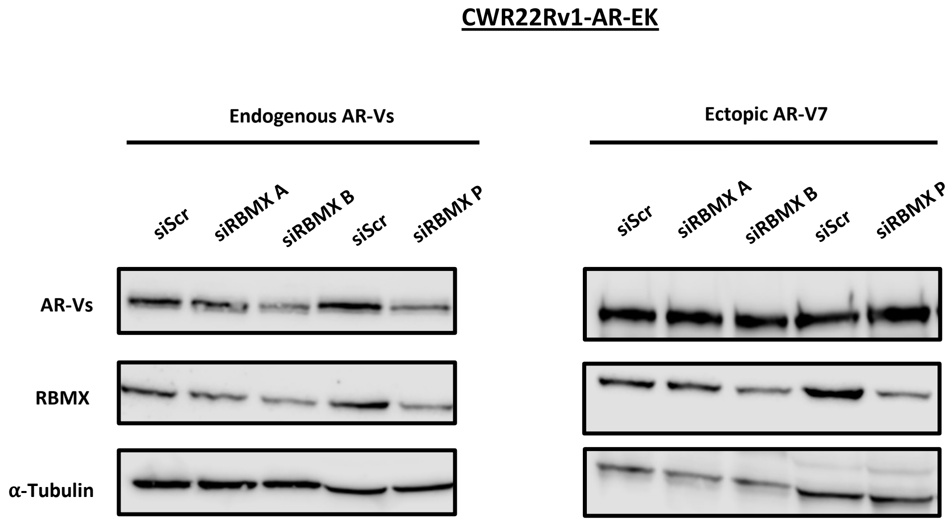

B..

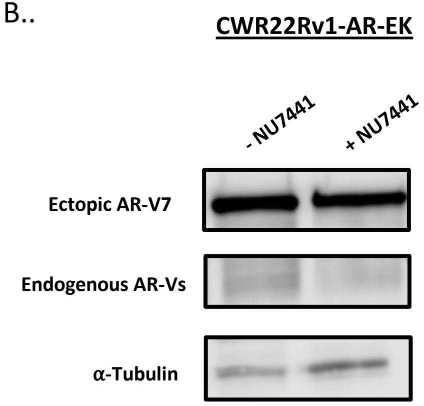

Supplementary Figure S28

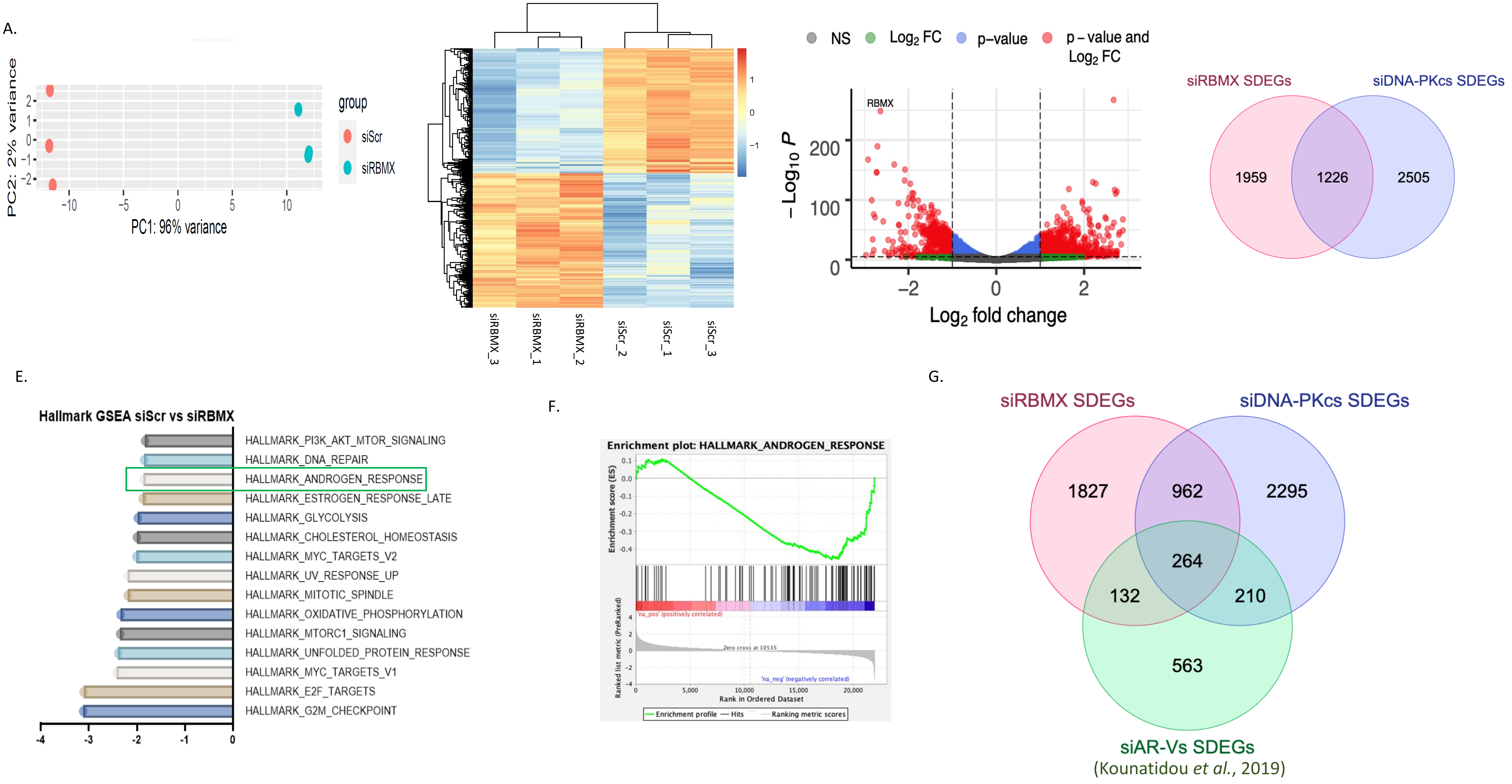

Supplementary Figure S29

A.

siScr vs siRBMX: events

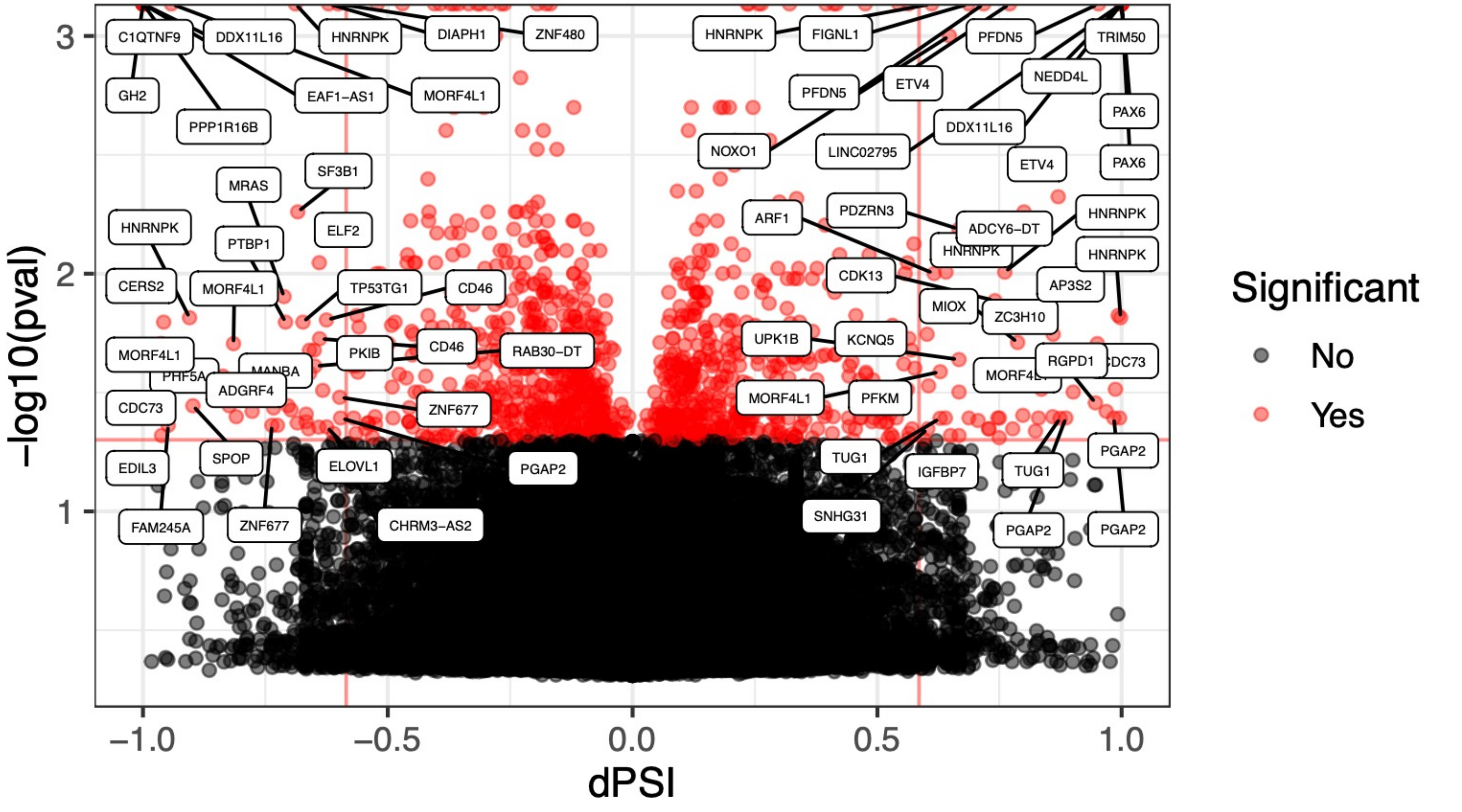

FDR < 0.05

B.

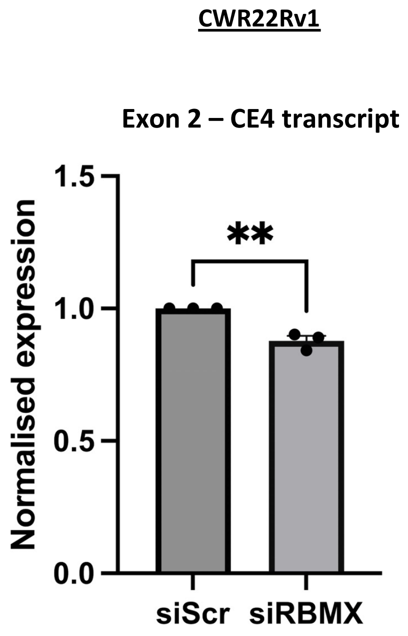

Supplementary Figure S30

A.

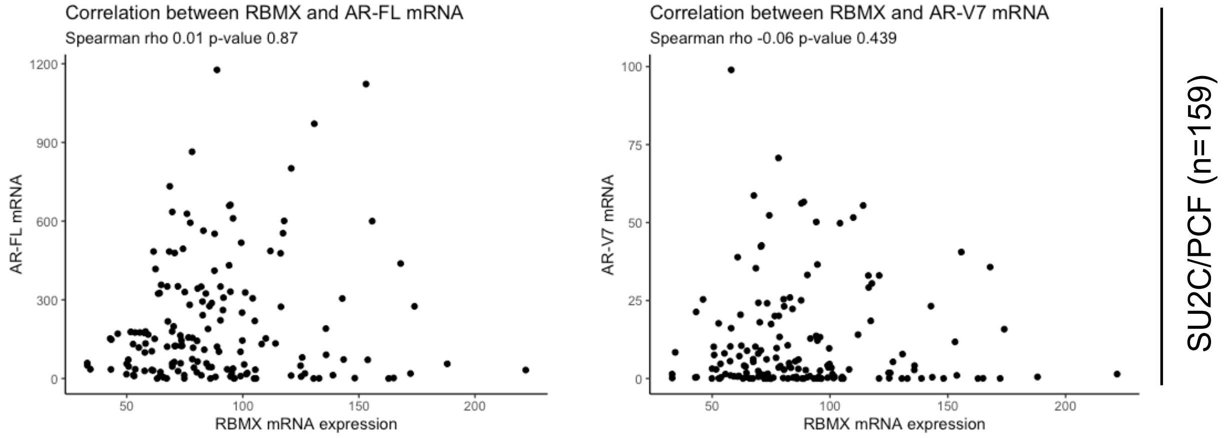

B.

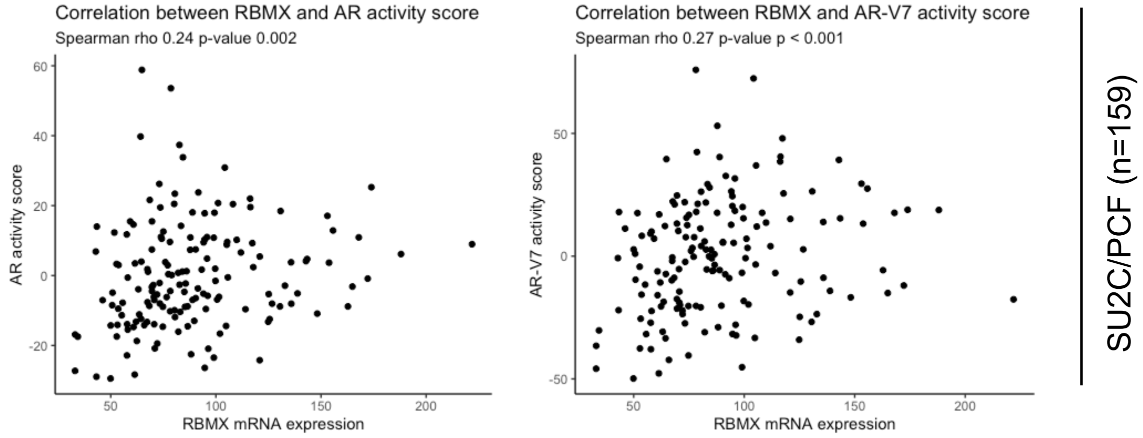

Supplementary Figure S31

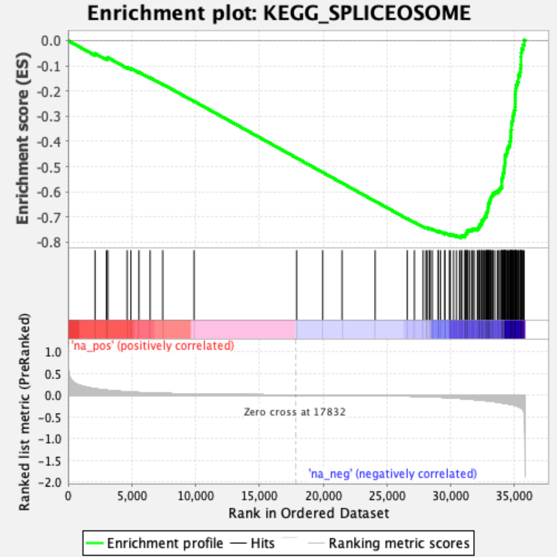

Dataset	Dylgjeri_NU7441_v_control.rnk
Phenotype	NoPhenotypeAvailable
Upregulated in class	na_neg
GeneSet	KEGG_SPLICEOSOME
Enrichment Score (ES)	-0.7810325
Normalized Enrichment Score (NES)	-2.3642318
Nominal p-value	0.0
FDR q-value	0.0
FWER p-Value	0.0

Figure 1

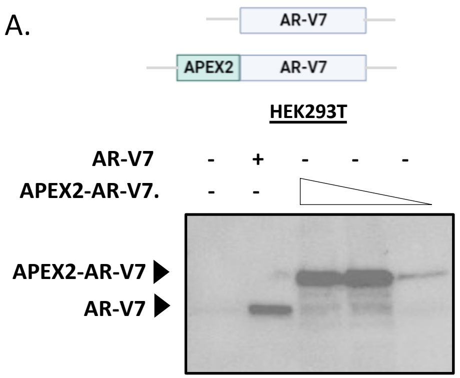

Full unedited gel for Figure 1A

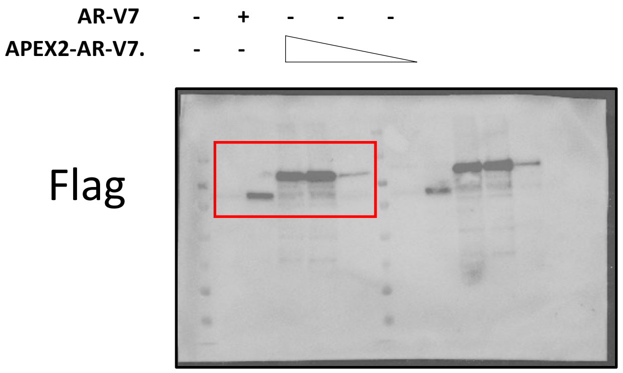

Figure 4

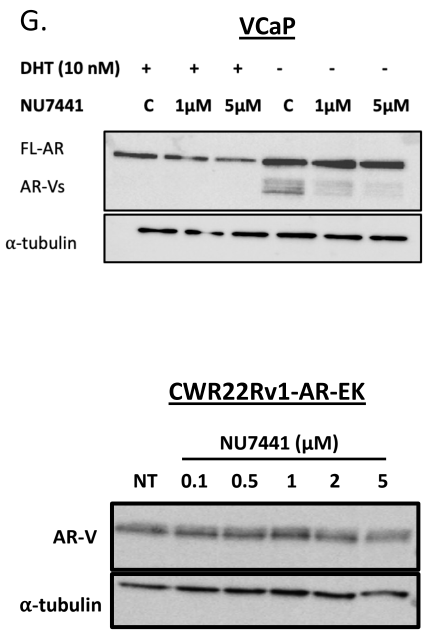

Full unedited gels for Figure 4G

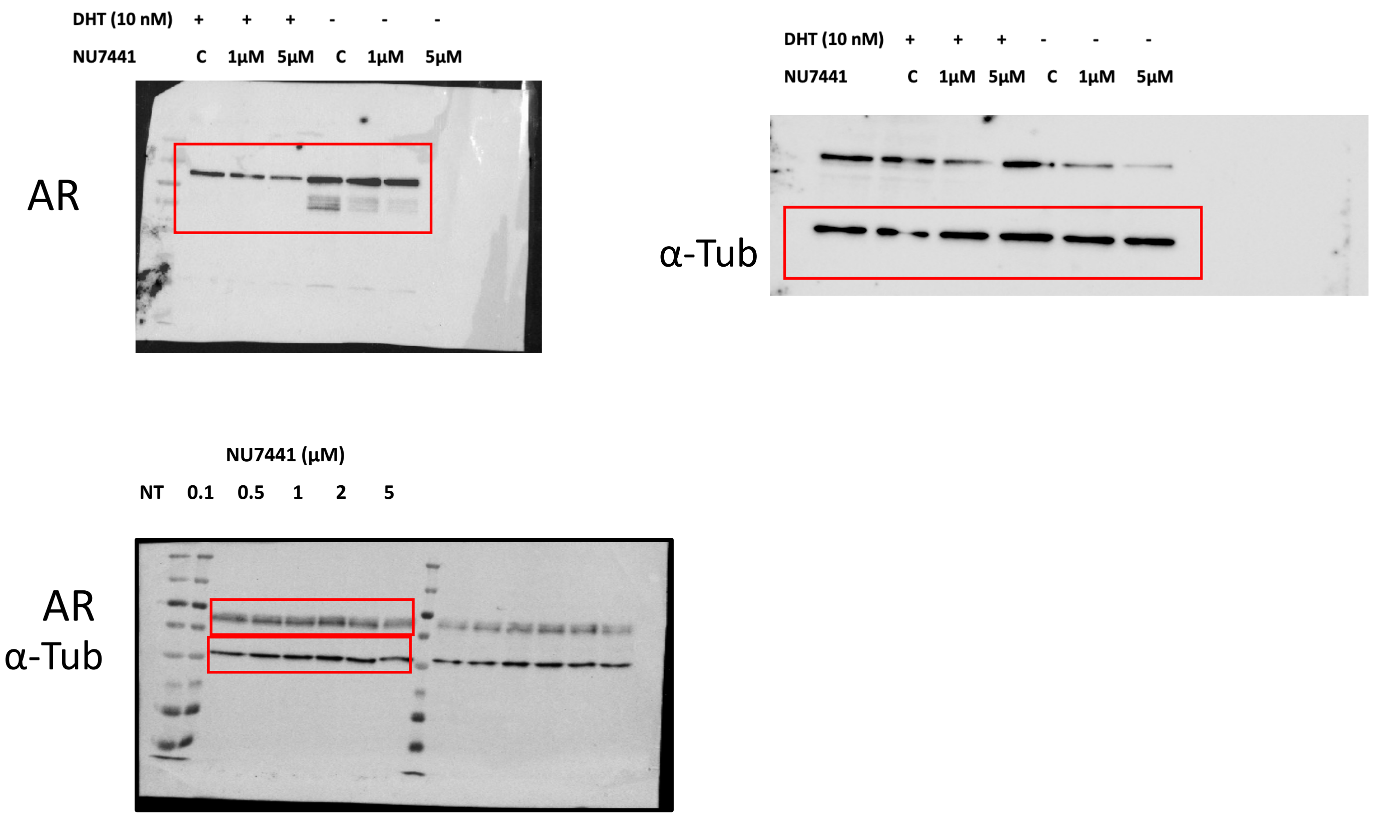

Figure 5

Full unedited gels for Figure 5C

C.

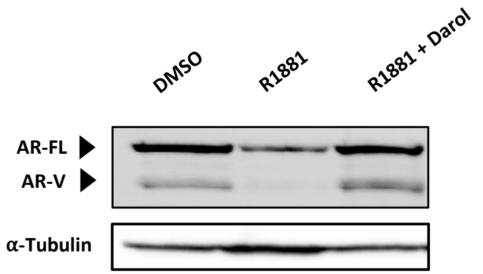

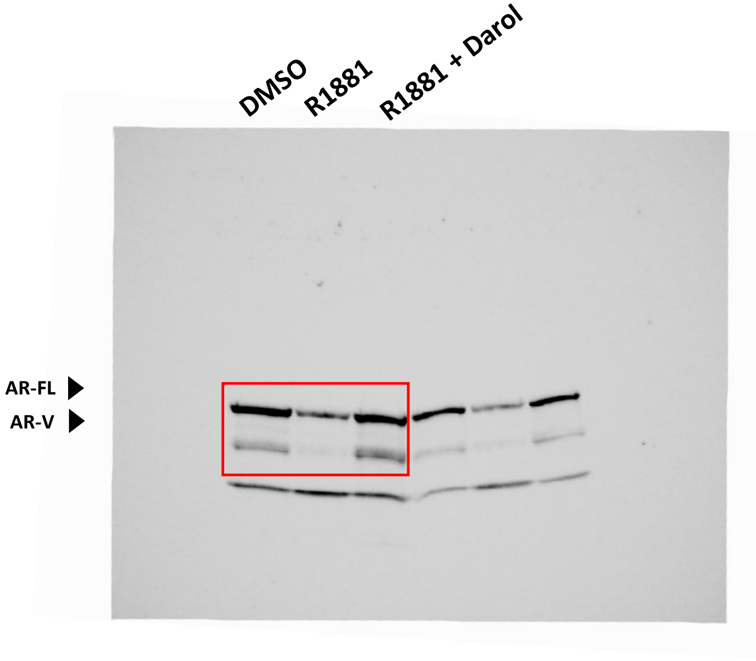

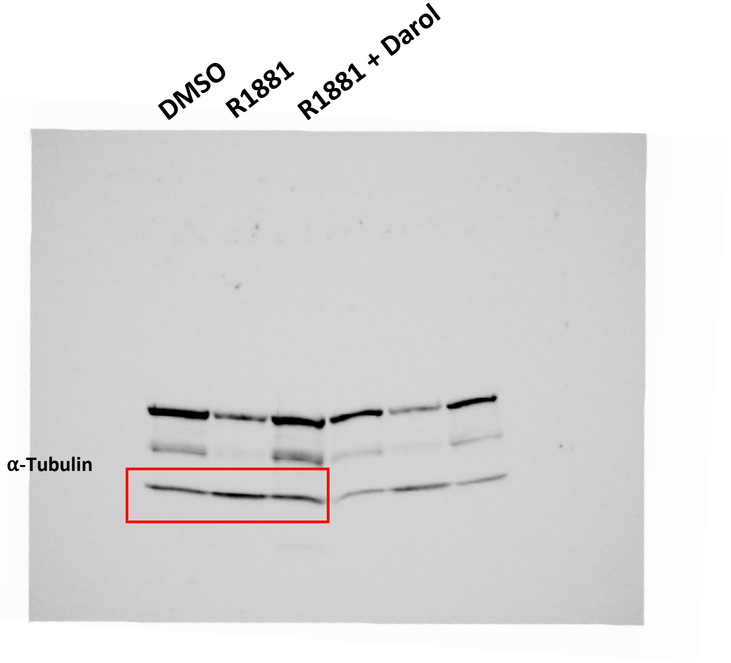

Figure 7

Full unedited gels for Figure 7

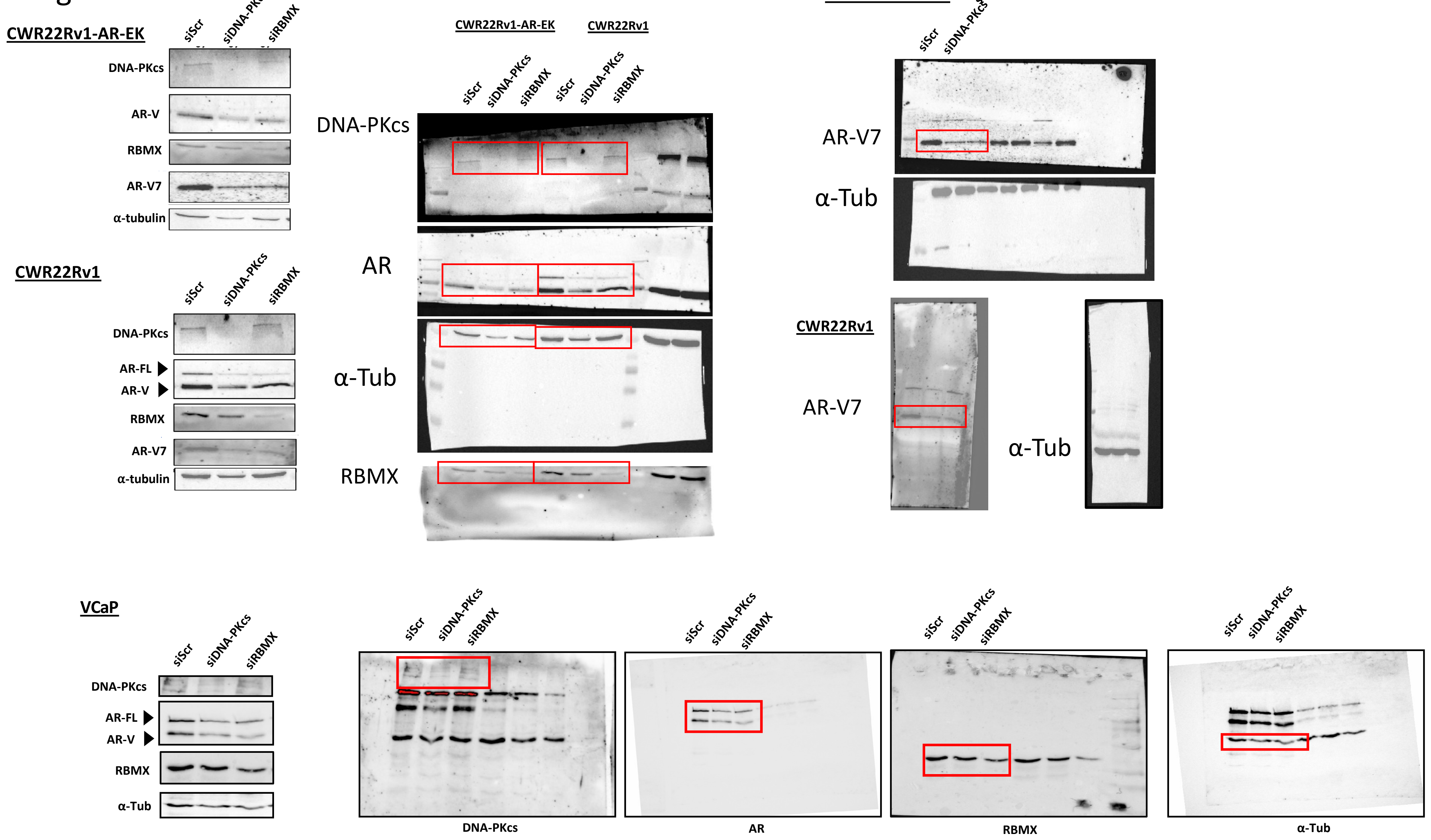

Figure 7 continued

Full unedited gels for Figure 7F

F.

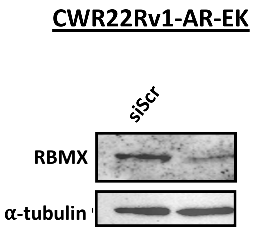

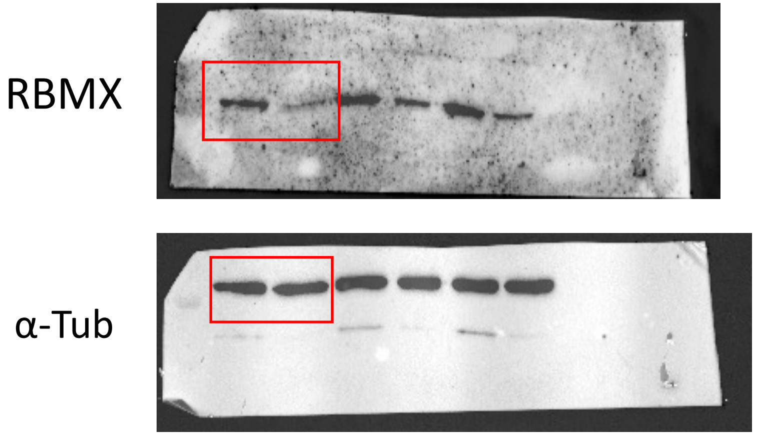

Supplementary figure 9

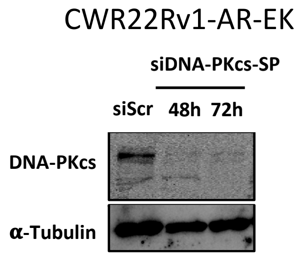

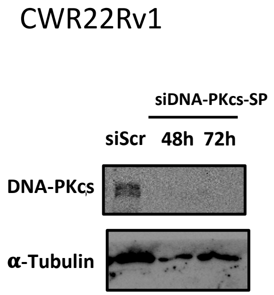

Full unedited gels for Supplementary Figure 9

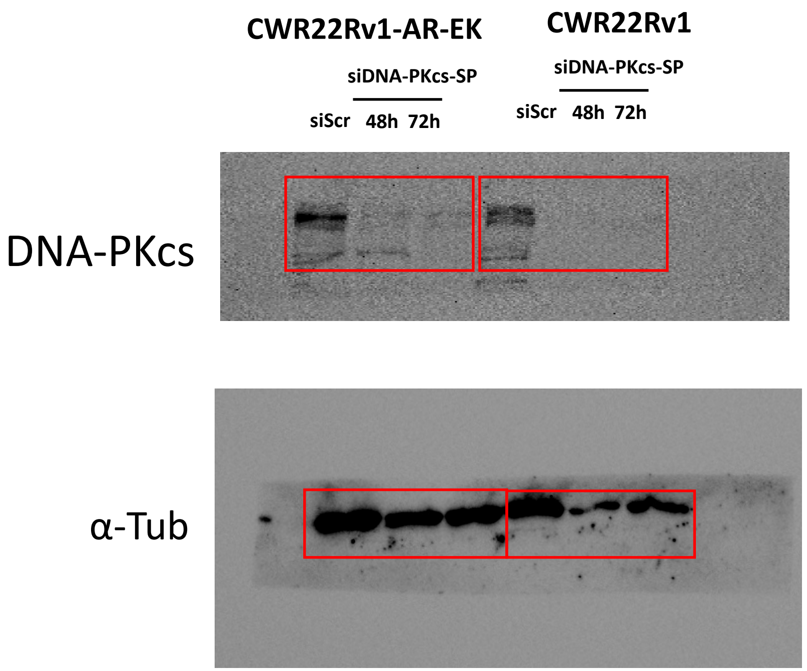

Supplementary Figure S12

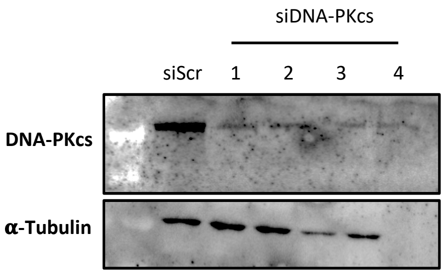

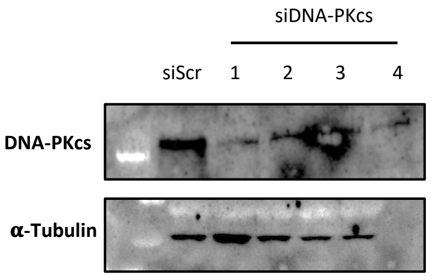

Full unedited gels for Supplementary Figure 12

CWR22Rv1-AR-EK

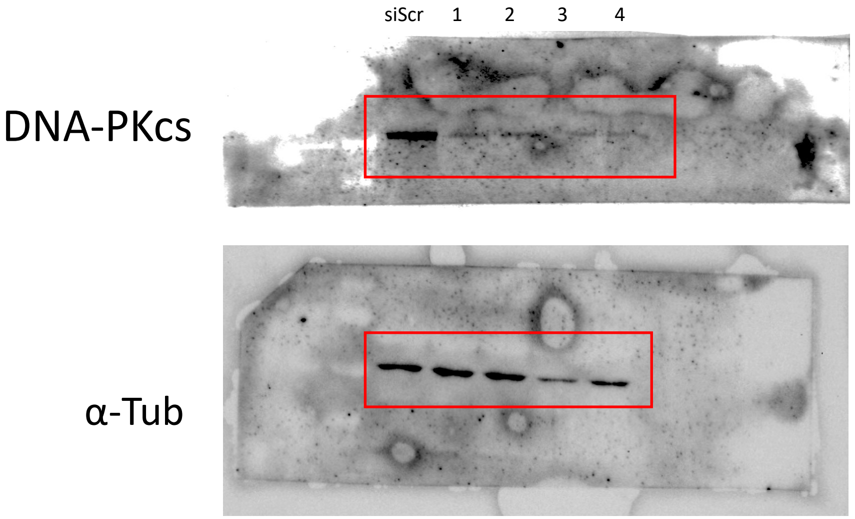

CWR22Rv1

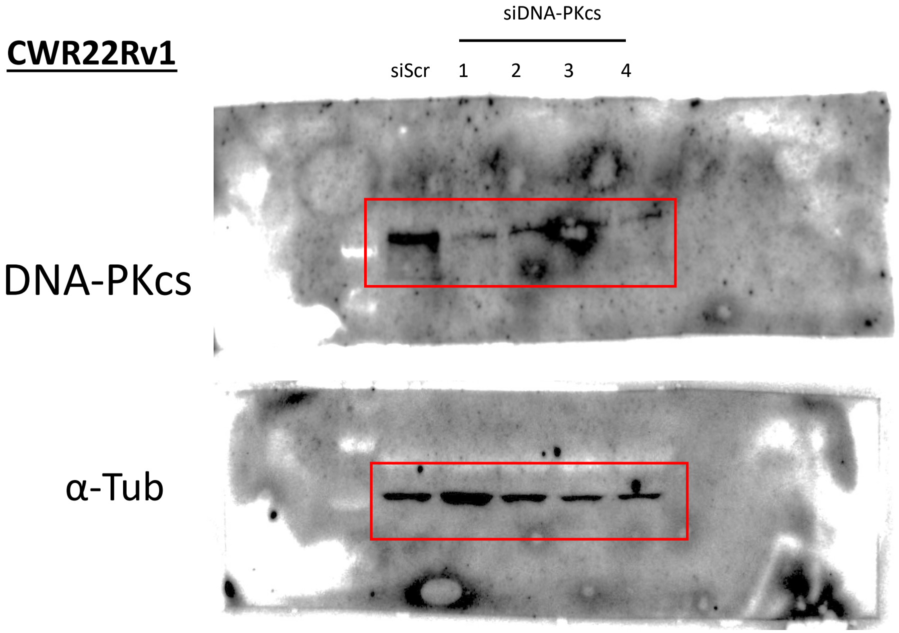

Supplementary Figure S25A

Full unedited gels for Supplementary Figure S25A

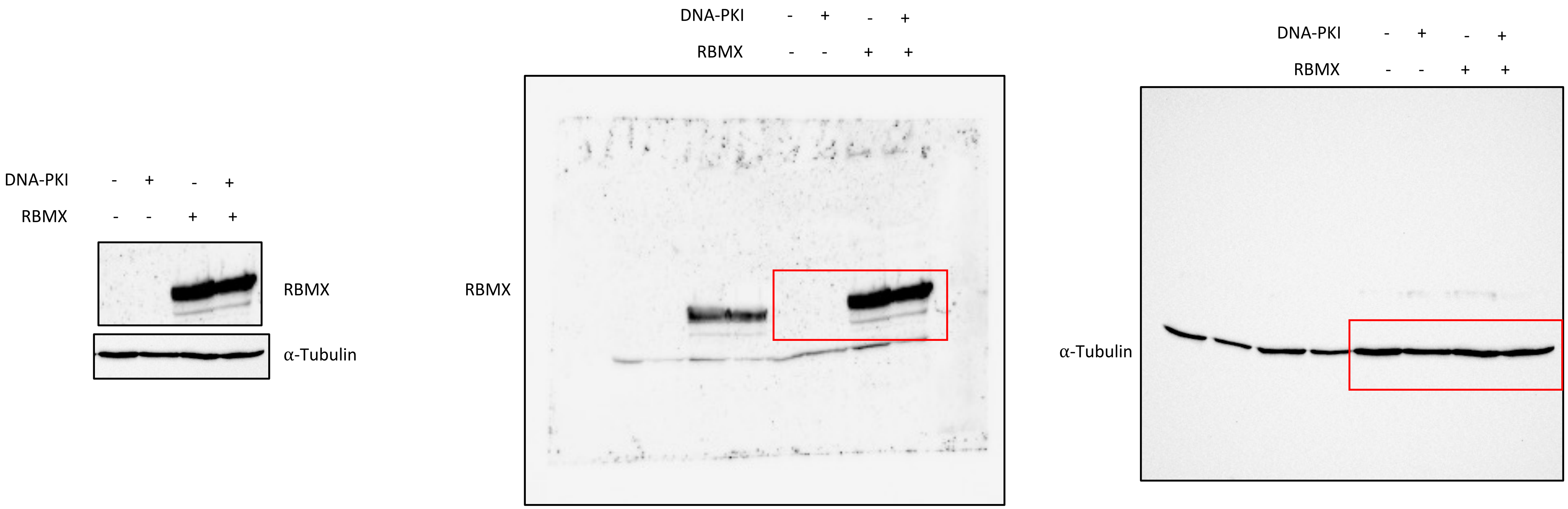

Supplementary Figure S25B

Full unedited gels for Supplementary Figure S25B

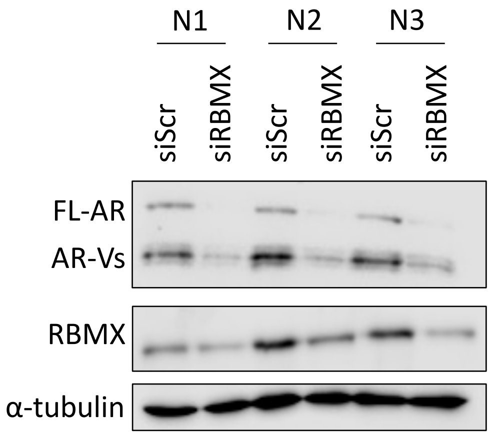

α-tubulin

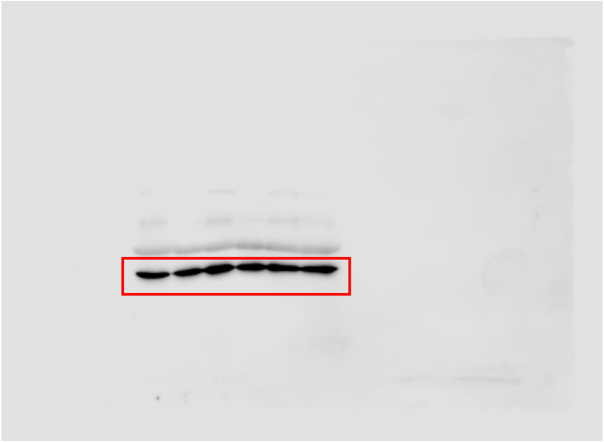

AR

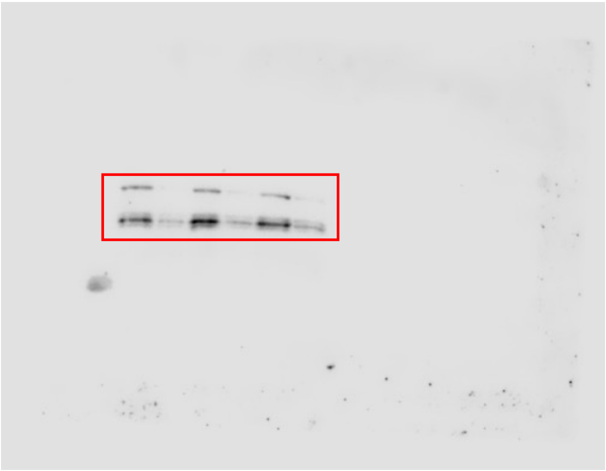

RBMX

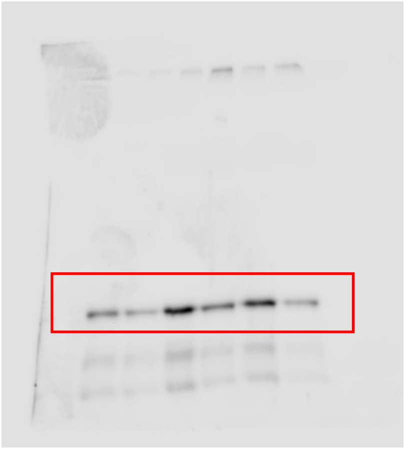

Supplementary Figure S27

Full unedited gels for Supplementary Figure S27A and S27B

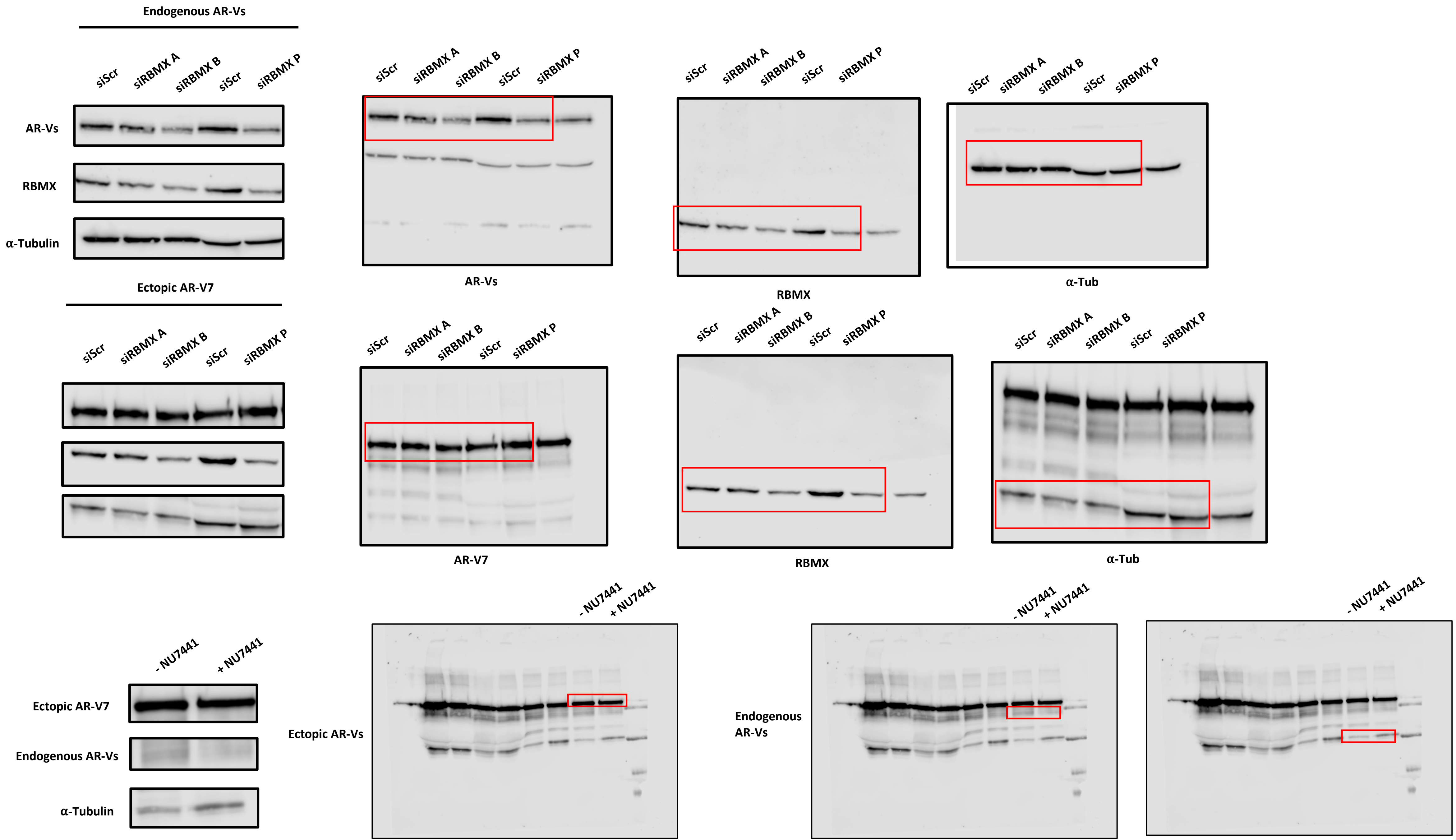

Supplementary Figure S26

Full unedited gels for Supplementary Figure S26

A.

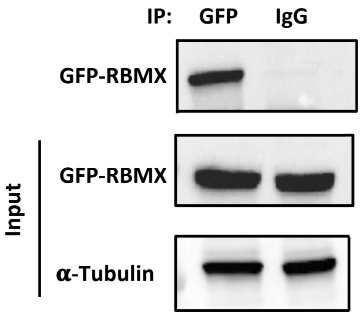

GFP

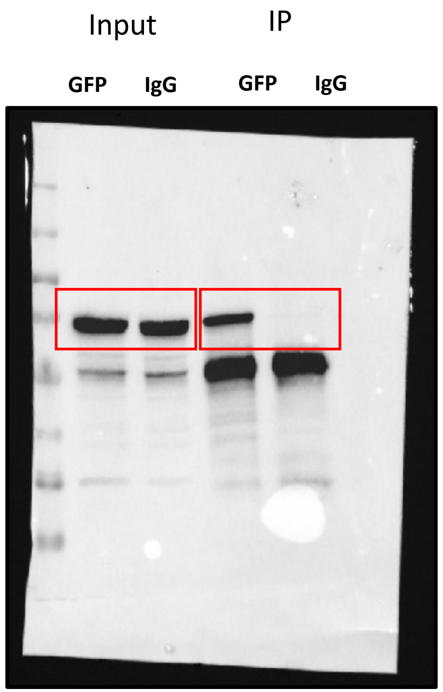

α-Tub

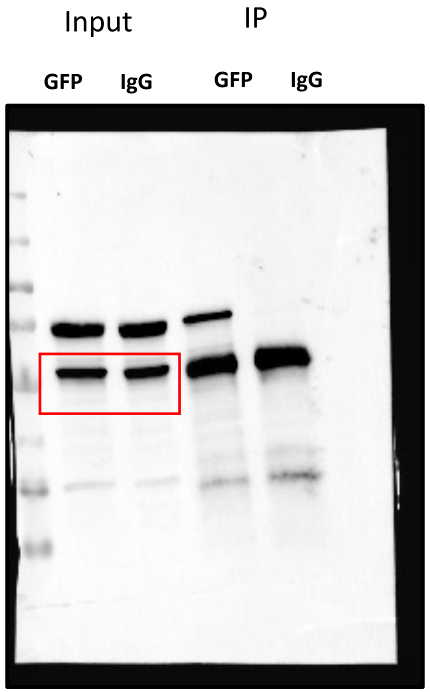

Supplement: Supplemental data [file jci-133-169200-s101.pdf]
